# Biosynthesis of the nitrogenase active-site cofactor precursor NifB-co in *Saccharomyces cerevisiae*

**DOI:** 10.1073/pnas.1904903116

**Published:** 2019-11-25

**Authors:** Stefan Burén, Katelin Pratt, Xi Jiang, Yisong Guo, Emilio Jimenez-Vicente, Carlos Echavarri-Erasun, Dennis R. Dean, Ishtiaq Saaem, D. Benjamin Gordon, Christopher A. Voigt, Luis M. Rubio

**Affiliations:** ^a^Centro de Biotecnología y Genómica de Plantas, Universidad Politécnica de Madrid, Instituto Nacional de Investigación y Tecnología Agraria y Alimentaria, Pozuelo de Alarcón, 28223 Madrid, Spain;; ^b^Broad Institute of MIT and Harvard, Cambridge, MA 02142;; ^c^Department of Chemistry, Carnegie Mellon University, Pittsburgh, PA 15213;; ^d^Department of Biochemistry, Virginia Polytechnic Institute, Blacksburg, VA 24061;; ^e^Departamento de Biotecnología-Biología Vegetal, Escuela Técnica Superior de Ingeniería Agronómica, Alimentaría y de Biosistemas, Universidad Politécnica de Madrid, 28040 Madrid, Spain;; ^f^Synthetic Biology Center, Department of Biological Engineering, Massachusetts Institute of Technology, Cambridge, MA 02139

**Keywords:** nitrogen fixation, synthetic biology, nif genes, combinatorial design, mitochondria

## Abstract

The radical *S*-adenosylmethionine (SAM) enzyme NifB occupies a central and essential position in nitrogenase biogenesis. NifB catalyzes the formation of an [8Fe-9S-C] cluster, called NifB-co, which constitutes the core of the active-site cofactors for all 3 nitrogenase types. Here, we produce functional NifB in aerobically cultured *Saccharomyces cerevisiae*. Combinatorial pathway design was employed to construct 62 strains in which transcription units driving different expression levels of mitochondria-targeted *nif* genes (*nifUSXB* and *fdxN*) were integrated into the chromosome. Two combinatorial libraries totaling 0.7 Mb were constructed: An expression library of 6 partial clusters, including *nifUSX* and *fdxN*, and a library consisting of 28 different *nifB* genes mined from the Structure–Function Linkage Database and expressed at different levels according to a factorial design. We show that coexpression in yeast of the nitrogenase maturation proteins NifU, NifS, and FdxN from *Azotobacter vinelandii* with NifB from the archaea *Methanocaldococcus infernus* or *Methanothermobacter thermautotrophicus* yields NifB proteins equipped with [Fe-S] clusters that, as purified, support in vitro formation of NifB-co. Proof of in vivo NifB-co formation was additionally obtained. NifX as purified from aerobically cultured *S. cerevisiae* coexpressing *M. thermautotrophicus* NifB with *A. vinelandii* NifU, NifS, and FdxN, and engineered yeast SAM synthase supported FeMo-co synthesis, indicative of NifX carrying in vivo-formed NifB-co. This study defines the minimal genetic determinants for the formation of the key precursor in the nitrogenase cofactor biosynthetic pathway in a eukaryotic organism.

## Significance

Nitrogen is a constituent of many essential biomolecules and plentiful on earth as inert N_2_ gas. For its assimilation by eukaryotes, N_2_ must be converted to a metabolically tractable form such as ammonium. Such conversion is catalyzed by nitrogenase, an enzyme produced by a select group of microorganisms called diazotrophs. Crop yields necessary to feed the world's population have critically depended on applying nitrogenous fertilizers. Incorporation of prokaryotic determinates required to produce active nitrogenase into crop plants would have enormous economic and environmental benefits. The active-site cofactors of all nitrogenases have a common metallocluster precursor synthesized by NifB. Here, we identify the genetic determinants for NifB function in mitochondria of *Saccharomyces cerevisiae*, thereby advancing prospects to generate N_2_-fixing crops.

Biological N_2_ fixation, the reduction of inert N_2_ gas to ammonia, is catalyzed by nitrogenases, O_2_-sensitive metalloenzymes having 2 interacting components termed dinitrogenase and dinitrogenase reductase (1, 2). There are 3 structurally and functionally similar but genetically distinct nitrogenases, distinguished, in part, by the metal composition of their corresponding active-site cofactors. For the most abundant “Mo-dependent” enzyme, dinitrogenase reductase (a homodimer) is encoded in *nifH*, dinitrogenase (a hetero-tetramer) is encoded in *nifD* and *nifK*, and the active-site is occupied by the iron-molybdenum cofactor (FeMo-co), a [7Fe-9S-C-Mo-homocitrate] cluster (3, 4). FeMo-co is produced in a pathway independent of NifDK and it is inserted into apo-NifDK to generate active NifDK.

**Fig. 1.**
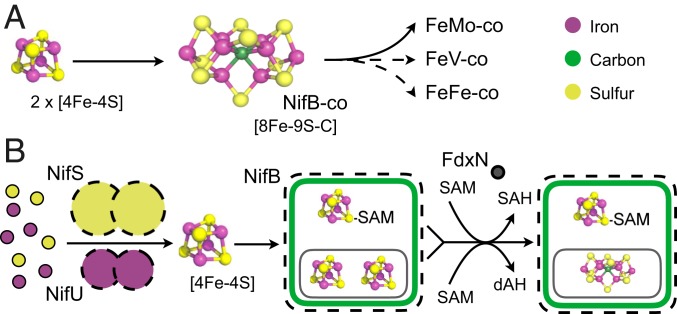
Maturation of the nitrogenase active-site metalloclusters, according to current knowledge about NifB mechanism. (*A*) NifB-co is the intermediate metal cluster common to the biosynthesis of all 3 types of nitrogenase active sites. (*B*) NifS and NifU proteins synthesize the [4Fe-4S] clusters that are delivered to NifH and NifB (and other Nif proteins). NifB harbors 3 distinct clusters, where the [4Fe-4S] SAM-coordinated cluster uses radical SAM chemistry to fuse the 2 [4Fe-4S] precursors (encircled) and inserts a central carbide and an additional sulfur, forming NifB-co [8Fe-9S-C]. The exact function of FdxN is not known but has been proposed to provide electrons required for NifB-co biosynthesis. Proteins are illustrated according to their sizes and multimeric arrangements. Relevant references can be found in the text.

The complete process of FeMo-co biosynthesis can be performed in vitro (5). A key enzyme for this process is NifB (6), which generates an [8Fe-9S-C] cluster called NifB-co (7, 8), which functions as obligate precursor to FeMo-co and also to the active-site cofactors of the other nitrogenase types (5) (Fig. 1*A*). It is therefore essential to the biogenesis of all nitrogenases (9). Current data indicate that NifB harbors a catalytic [4Fe-4S] *S*-adenosylmethionine (SAM)-coordinated cluster and 2 additional [4Fe-4S] accessory clusters. NifB uses radical SAM chemistry to initiate NifB-co formation, which is accomplished by fusing its accessory clusters and inserting one S and one C atom (6, 8, 10–12) (Fig. 1*B*). There is evidence to support that the NifB [4Fe-4S] clusters are initially assembled on the NifU scaffold, acting together with the cysteine desulfurase NifS (13). Although NifB-co can be transferred directly to NifEN in vitro (5), where it is finally converted into FeMo-co, the NifX protein can act as carrier of NifB-co from NifB to NifEN in vivo and in vitro (14). When FeMo-co biosynthesis is interrupted at the level of NifEN, NifX accumulates NifB-co, facilitating its isolation and characterization. No change in spectral or functional properties of NifB-co has been observed upon binding to NifX, indicating that this protein functions solely as carrier (8). Finally, the product of *fdxN*, which is cotranscribed with *nifB* in *Azotobacter* *vinelandii*, was shown to be important for NifB-co biosynthesis, but its exact role is unknown (15).

Recent transfer of nitrogenase genes into *Saccharomyces cerevisiae* showed that mitochondria offer a suitable environment to assemble active NifH during aerobic growth (16). However, expression of *A. vinelandii* NifB in *S. cerevisiae* resulted in accumulation of insoluble and inactive protein (17). Not all NifB proteins have the same domain architecture. Most variants contain either a standalone SAM domain or a SAM domain together with a carboxyl (C)-terminal NifX-like domain (e.g., *A. vinelandii* NifB) (18). A His-tagged single-domain NifB variant from the thermophile archaeon *Methanocaldococcus infernus* produced in yeast could be partly solubilized upon heat treatment and purified using metal-affinity chromatography. This as-isolated form of NifB was loaded with ∼3 Fe atoms per monomer (17), similar to the same form of NifB produced by and purified from *Escherichia coli* cells (12). These experimental results contrast with the 12 Fe atoms expected for a fully loaded NifB protein harboring 3 [4Fe-4S] clusters (11). In this case, yeast-expressed His-tagged NifB was only active following [Fe-S] cluster reconstitution, and, therefore, the exact genetic requirements for producing a functional NifB in yeast could not be determined.

Targeting all required gene products to mitochondria represents a challenge as the various N_2_-fixing systems are very complex from both genetic and biochemical perspectives (19, 20). Recent work has revealed possible ways that separately, or in combination, provide opportunities to simplify this problem. One of these involves functional replacement of certain required prokaryotic components by proteins of plant origin (21, 22), and another involves fusion of certain *nif* genes to produce polyproteins amenable to posttranslational processing to yield individually active components (23, 24). Transfer of the minimal suite of genes required to produce active Mo-dependent nitrogenase in a model prokaryotic system to the chromosome of *S. cerevisiae* has been reported. In this case, expression levels and targeting approaches were balanced by designing combinatorial libraries comprising 9 *nif* genes of the *A. vinelandii* Mo-nitrogenase (*nifHDKUSMBEN*) (25). Ultimately, expression and mitochondria targeting were observed for all 9 gene products, and the NifDK tetramer was correctly assembled. However, biochemical characterization revealed that NifDK produced in this system lacked FeMo-co, resulting in accumulation of inactive and unstable apo-NifDK. Thus, FeMo-co assembly remains a major barrier for the generation of a N_2_-fixing eukaryote.

Here, we apply combinatorial pathway design and assembly to investigate the function of 28 selected *nifB* gene products in aerobically cultured yeast. Through iterative testing involving 62 NifB pathway variants, we found that NifB from *M. infernus* and *Methanothermobacter thermautotrophicus* can be produced in yeast. In both cases, coexpression of *A. vinelandii* NifU, NifS, and FdxN synthesized NifB that, as purified, supported FeMo-co formation in vitro. Importantly, one *M. thermautotrophicus* NifB pathway generated NifX that contained in vivo-formed NifB-co.

## Results

### Library Strategy and Design.

The starting hypothesis was that genetic factors influencing NifB activity would include expression levels of NifB and the accessory proteins NifU, NifS, NifX, and FdxN, as well as intrinsic properties encoded in distinct NifB variants. Different combinations of these factors were tested by applying pathway library design (25, 26), which enabled combinatorial construction of strains containing specific selection of genes, regulatory sequences, targeting signals, and purification tags (*Materials and Methods*).

**Fig. 2.**
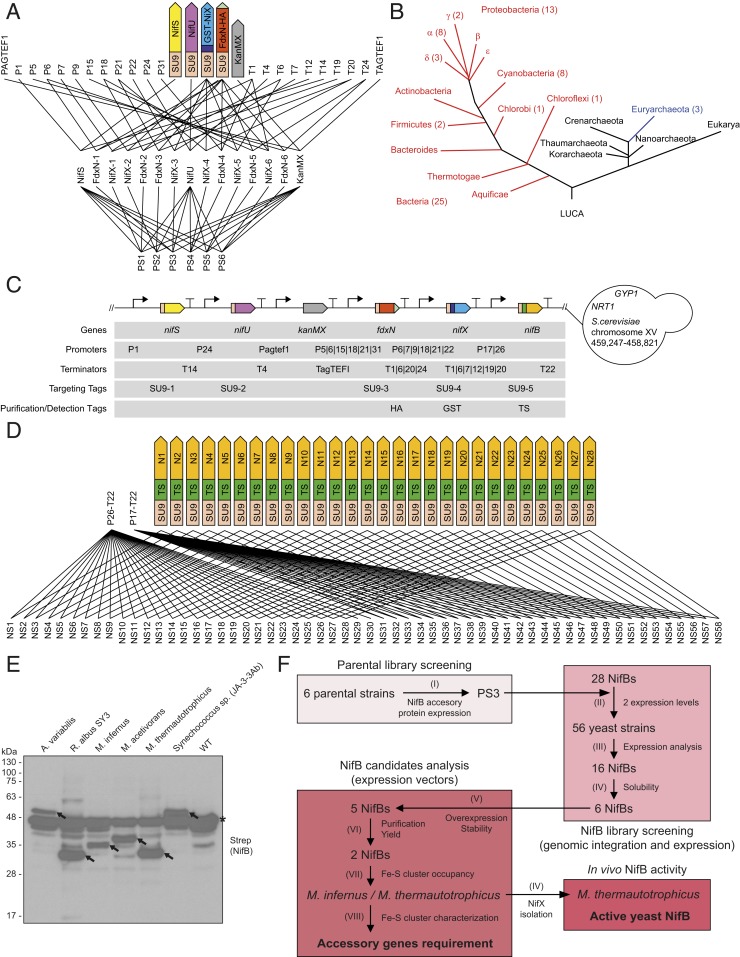
Generation of yeast NifB library. (*A*) *S. cerevisiae* CEN.PK113−7D was integrated with NifS, NifU, NifX (GST-NifX), and FdxN (FdxN-HA) at varying expression levels to create 6 parental strains (PS) for NifB expression. (*B*) Phylogenic distribution of *nifB* genes selected for the library. (*C*) The genetic schematic of the *nif* constructs and their location in the *S. cerevisiae* genome are shown. Promoters and terminators for FdxN, NifX, and NifB that were utilized in different combinations within the library are separated by “|” symbols. The cluster was inserted in the direction of leading-strand replication, between 459,247 and 458,821 bp on chromosome-XV in *S. cerevisiae* CEN.PK113−7D. Part sequences are provided in *SI Appendix*. (*D*) Fifty-six strains were constructed to express 28 *nifB* genes at 2 levels in the parental strain PS3. Promoters and terminator for high (P26-T22) and medium (P17-T22) *nifB* expression were chosen from previous designs (25). Each line represents an assembly step (see *Materials and Method* and *SI Appendix* for further details). (*E*) Western blot analysis of soluble protein extracts of wild-type *S. cerevisiae* (WT) and *S. cerevisiae* strains expressing the 6 soluble NifB candidates as indicated. Arrows indicate migration of full-length NifB proteins. Star indicates nonspecific signal. Ponceau-stained membrane is shown in *SI Appendix*, Fig. S5*C*. (*F*) Workflow followed in this report. Six parental yeast strains were generated to tune expression of *A. vinelandii nifS*, *nifU*, *fdxN*, and *nifX* (I) (A and *SI Appendix*, Fig. S1). Twenty-eight *nifB* genes at 2 different expression levels were integrated with accessory genes as in parental strain 3 (PS3), generating 56 yeast strains (II) (B–D). Sixteen distinct NifB proteins could be detected in yeast protein extracts (III) (*SI Appendix*, Fig. S5*A*), of which 6 accumulated as soluble proteins (IV) (*E*). These *nifB* candidate genes were transferred to high-expression vectors, resulting in 5 mainly intact full-length NifB proteins (V) (*SI Appendix*, Fig. S9). Two of the candidates could be purified at high yields (VI) (*SI Appendix*, Fig. S10), where the *M. infernus* NifB accumulated with higher Fe-S cluster occupancy (VII) (*SI Appendix*, Fig. S11) and was selected for analysis of activity, Fe-S clusters, and accessory gene dependency (VIII) (Figs. 3 and 4 and *SI Appendix*, Figs. S14–S18). In vivo NifB activity was shown by isolation of a NifX/NifB-co complex in a *S. cerevisiae* strain expressing the *M. thermautotrophicus* NifB protein (IV) (Fig. 5 and *SI Appendix*, Fig. S24). A summary of the screen is shown in *SI Appendix*, Table S3.

First, we designed a library of 6 parental strains to optimize stable expression of *A. vinelandii nifU*, *nifS*, *nifX*, and *fdxN* genes postulated as necessary to determine NifB activity in yeast. For *nifU* and *nifS,* expression was controlled by promoter/terminator combinations previously used (25), whereas *fdxN* and *nifX* expression was newly designed with the constraint that NifX should be equal or up to 36-fold higher than FdxN (*SI Appendix*, Fig. S1 *A* and *B* and Tables S1 and S2). The rationale was that NifX expression levels have been reported to be generally higher than other Nif proteins involved in FeMo-co synthesis (27) and that NifX can stabilize produced NifB-co (8). FdxN carried a C-terminal HA-tag to facilitate immunoblot detection (Fig. 2*A*), while NifX was fused to the C-terminal end of glutathione *S*-transferase (GST) to facilitate purification. All proteins were targeted to the mitochondria of *S. cerevisiae* using sequence variants of the SU9 signal selected to limit undesired homologous recombination (Fig. 2*A* and *SI Appendix*, *Supplementary Text* sequences). Parental strain 3 produced the most consistent protein expression and was used as foundation for the second library (*SI Appendix*, Fig. S1 *C* and *D*).

NifB proteins are notoriously difficult to study due to instability (11–13, 28). To find NifB proteins better suited for expression in eukaryotic cells, we mined the Structure–Function Linkage Database (29) for 28 different *nifB* gene sequences and synthesized them with codon optimization for expression in *S. cerevisiae*. Because the ultimate goal is to engineer nitrogenase in plants, *nifB* genes were selected according to the following criteria: 1) those originating from or being associated to photosynthetic organisms, 2) those originating from organisms with aerobic lifestyle, and 3) those whose products have been previously shown to be functional in NifB-co formation (Fig. 2*B*, *SI Appendix*, Table S3, and Dataset S1). As it was previously shown that somewhat-soluble NifB protein from the archaeon *M. infernus* could be produced in *S. cerevisiae* (17), the selection included additional archaeal *nifB* genes. Thus, 25 *nifB* genes were of bacterial origin (13 Proteobacteria, 8 Cyanobacteria, 2 Firmicutes, 1 Chlorobi, and 1 Chloroflexi) and 3 of archaeal origin. Eighteen harbored the C-terminal NifX-like domain present in NifB proteins with a 2-domain structure, while the remaining 10 candidates contained only the conserved SAM-radical domain (*SI Appendix*, Table S3 and Dataset S1) (18).

A library of 56 yeast strains was built by factorial design in which each *nifB* was tested at 2 expression levels predicted to differ by 9-fold (Fig. 2 *C* and *D* and *SI Appendix*, Fig. S1*A* and Table S2), together with *nifU*, *nifS*, *fdxN*, and *nifX* as expressed in parental strain 3 (Fig. 2*A* and *SI Appendix*, Table S1). To facilitate NifB detection and isolation, a purification tag was placed between the amino (N)-terminal SU9 mitochondria targeting signal and the NifB-encoding region (Fig. 2*D* and *SI Appendix*, *Supplementary Text* sequences). Three different tags (10× His, 1× Strep, and 2× Strep) were tested for their effect on expression and solubility of NifB from *M. infernus*, used as reference protein (*SI Appendix*, Fig. S2, Table S4, and *Supplementary Text* sequences). Because the solubility of the Twin-Strep (TS)-tagged NifB protein was highest (*SI Appendix*, Fig. S3), and because Strep-Tactin–based purification procedures do not rely on metal-affinity resins that can remove the labile [4Fe-4S] clusters carried by NifB proteins, the TS-tag was chosen for the library screen (Fig. 2*D* and *SI Appendix*, *Supplementary Text* sequences). The enhanced solubility of *M. infernus* TS-NifB at moderate temperature was unexpected as purification of a C-terminally His-tagged version required prior heat treatment (17). Temperature-independent solubility of this engineered variant was confirmed (*SI Appendix*, Fig. S4 and Table S4), suggesting that addition of a neutral N-terminal extension (such as TS) could prevent its aggregation and/or membrane sequestration.

Sixteen distinct *nifB* genes generated polypeptides with migration in sodium dodecyl sulfate (SDS) gels that corresponded to those expected after mitochondrial import and SU9 processing (*SI Appendix*, Table S3 and Fig. S5*A*). Six of these NifB candidates accumulated mainly as soluble proteins (*SI Appendix*, Table S3 and Fig. S5 *B* and *C* and Fig. 2*E*) and were selected for further analysis. The workflow of this study is summarized in Fig. 2*F*.

### Purification of NifB Candidates Expressed in Aerobically Growing Yeast.

The 6 soluble NifB candidates were screened for activity using previously described NifB-dependent in vitro FeMo-co synthesis and insertion assay (5). This assay relies on the activation of apo-NifDK present in crude extracts of a NifB-deficient *A. vinelandii* strain (6, 30). Soluble extracts from aerobic flask-cultured *S. cerevisiae* strains were subject to anaerobic small-scale Strep-Tactin pull-downs to enrich NifB, which was then tested for NifB activity. No apo-NifDK activation was detected in any of the samples (*SI Appendix*, Fig. S6 *A* and *B*), and Western blot analysis showed that NifB levels were insufficient to support this screening assay (*SI Appendix*, Fig. S6*C*). Sufficient biomass was obtained from aerobically grown fermenter cultures of strain SB187Y expressing *M. infernus* NifB, which permitted the purification of approximately 3 mg of NifB (*SI Appendix*, Fig. S7 *A*–*C*). As-isolated NifB lacked activity, as previously reported (*SI Appendix*, Fig. S7*D*). It was observed that FdxN accumulation in fermented cells decreased over time and that GST-NifX suffered degradation, which precluded NifX purification (*SI Appendix*, Fig. S7*E*). Differential expression of NifB and FdxN could be problematic as FdxN is important for in vivo NifB activity (15). The protein extracts were also prone to protein precipitation, which hindered further scale-up of the purification procedure as well as interpretation of the results.

Although NifB is not highly expressed under diazotrophic conditions in their natural hosts (27), in-depth biophysical characterization of the protein and its clusters require large amounts of pure protein. To investigate the effect of NifU, NifS, and FdxN on NifB [Fe-S] cluster content and activity, we transferred the genes encoding the 6 soluble NifB candidates identified in the library screening to yeast expression vectors (*SI Appendix*, Fig. S8, Table S4, and *Supplementary Text* sequences). The *nifX* gene was not included at this stage, because NifX is not required for NifB function and, in addition, it could trigger release of NifB-co from NifB. Expression of all genes was controlled by GAL regulatory elements previously used for effective production of NifU, NifS, FdxN, and NifB (17). All NifB candidates accumulated at high levels, except for the *Synechococcus sp.* JA-3-3Ab variant (*SI Appendix*, Fig. S9). NifB from *Methanosarcina acetivorans* produced significant levels of a faster migrating isoform indicating protein degradation, so this construct was excluded from further analyses. Aerobic fermenter cultures for the remaining 5 strains were prepared, and NifB proteins were purified. Only NifB from *M. thermautotrophicus* and *M. infernus* yielded mainly soluble protein when produced at the high levels required for their biochemical characterization (*SI Appendix*, Fig. S10). Notably, both *M. thermautotrophicus* and *M. infernus* are archaea, and their NifB proteins lack the C-terminal NifX-like domain (*SI Appendix*, Table S3). Ultraviolet (UV)-visible spectra indicated that both proteins accommodated [Fe-S] clusters (*SI Appendix*, Fig. S11). Because the cluster occupancy appeared higher in the *M. infernus* NifB and given previous experience with its analysis, this variant was selected for in-depth biophysical analysis and for testing the requirement of NifU, NifS, and FdxN for NifB function.

### Biophysical Properties of *M. infernus* NifB.

**Fig. 3.**
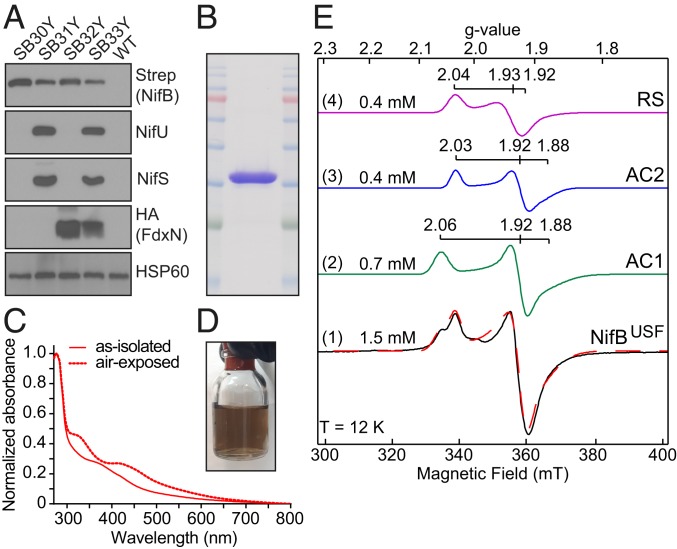
Expression and purification of *M. infernus* NifB carrying [Fe-S] clusters from yeast. (*A*) Immunoblot analysis of protein expression in total extracts of wild-type *S. cerevisiae* (WT) and *S. cerevisiae* strains used for NifB purifications (strain SB30Y, for expression of NifB*; SB31Y, for expression of NifB^US^; SB32Y, for expression of NifB^F^; SB33Y, for expression of NifB^USF^). (*B* and *C*) Coomassie staining (*B*) and as-isolated and air-exposed UV-visible spectra (*C*) of NifB^USF^ protein. (*D*) Appearance of NifB^USF^ (purification 13; *SI Appendix*, Table S5) obtained from 315 g of yeast cells following biotin-elution and desalting (total volume, about 13 mL). (*E*) X-band EPR spectra of NifB^USF^ (purification 14; *SI Appendix*, Table S5) (1) and subcomponents of spectral simulation for NifB^USF^ (2 to 4). Experimental data are shown in black solid lines, while overall spectral simulations are shown in red dotted lines. The *g* values of each species, spin concentration of the subcomponents, and cluster nomenclature (adapted from ref. 12) are indicated in the figure.

For clarity, mitochondria-targeted TS-NifB produced in yeast and in the absence of any other prokaryotic component is hereafter denoted as NifB*. NifB species produced in yeast in combination with mitochondrial NifU, NifS, and FdxN are denoted by a superscript of the corresponding coexpressed *nif* gene product. For example, the NifB species produced in combination with NifU, NifS, and FdxN is indicated as NifB^USF^ (*SI Appendix*, Fig. S12 and Table S4). Because *M. infernus* NifB^USF^ accumulated as a largely soluble protein, it could be purified as a dark brown protein (Fig. 3 *A*–*D*). The influence of coexpressing NifU, NifS, and FdxN separately, or in combination, on NifB capacity for SAM-dependent NifB-co formation could also be evaluated. Proper protein targeting to yeast mitochondria and subsequent processing was confirmed by migration of the corresponding proteins on SDS–polyacrylamide gel electrophoresis (SDS/PAGE) and, in the case of NifB, by N-terminal amino acid sequencing (Fig. 3*A* and *SI Appendix*, Fig. S13). Various NifB species anoxically isolated from aerated fermenter cultures of yeast cells yielded about 6 mg of protein per 100 g of cells, and the metal content and UV-visible spectra of these species were determined (*SI Appendix*, Figs. S14 and S15 and Table S5).

NifB is known to carry 3 distinct [4Fe-4S] clusters (12). One of these, designated RS, is associated with SAM and is a permanent catalytic cluster, whereas the other 2 are accessory clusters, designated AC1 and AC2, which are ultimately fused to form NifB-co. Because the UV-visible spectrum and Fe concentration indicated that the *M. infernus* NifB^USF^ and NifB^US^ species could have the highest cluster occupancy (Fig. 3*C* and *SI Appendix*, Fig. S15 and Table S5), it was of interest to examine the electron paramagnetic resonance (EPR) spectra of these proteins. The EPR spectrum of as-isolated *M. infernus* NifB^USF^ measured at 12 K exhibited S = 1/2 signals typical of reduced [4Fe-4S]^+^ clusters (Fig. 3*E* and *SI Appendix*, Fig. S16). This assignment was confirmed by temperature-dependent measurements, which showed signal disappearance above 70 K (*SI Appendix*, Fig. S17) (31). The signal was well reproduced by including 3 different [4Fe-4S]^+^ clusters in spectral simulations (Fig. 3*E*), having *g* values almost identical to those of the clusters found in the reconstituted *M. infernus* NifB expressed in *E. coli* (12). Total spin concentration of the S = 1/2 [4Fe-4S]^+^ signals was ∼1.5 mM, which translated to ∼6 mM Fe. The Fe concentration estimated by EPR was also in good agreement with chemical Fe quantitation (∼7 mM), indicating that almost all Fe in NifB^USF^ was in the form of [4Fe-4S]^+^ clusters. In contrast, only one type of [Fe_4_S_4_]^+^ clusters (AC1 cluster; ref. 12) was observed in the EPR spectrum of the as-isolated NifB^US^ proteins (*SI Appendix*, Fig. S18), while the RS and AC2 clusters were missing.

### Purification and Activities of *M. infernus* and *M. thermautotrophicus* NifB Species Produced in Yeast.

**Fig. 4.**
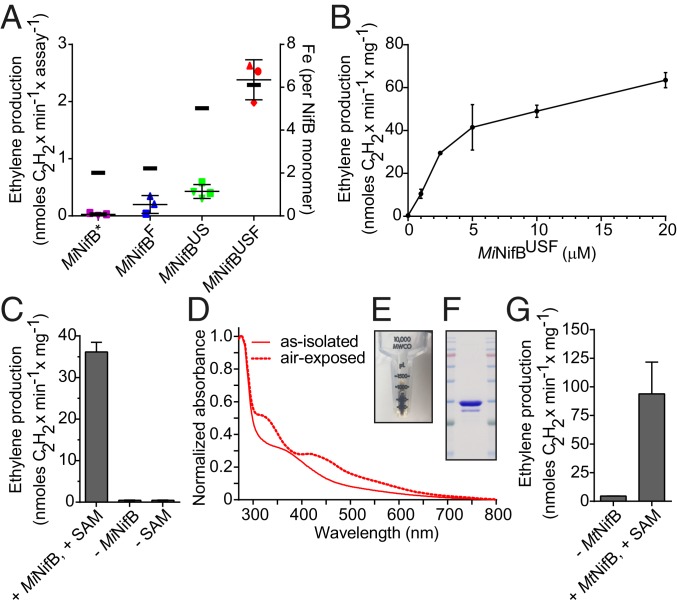
Genetic and biochemical requirements for NifB functionality. (*A*) In vitro synthesis of FeMo-co and apo-NifDK reconstitution assay using ∆*nifB A. vinelandii* (UW140) CFEs and 12.5 μM as-isolated *M. infernus* NifB* (purple), NifB^US^ (green), NifB^F^ (blue), or NifB^USF^ (red). Activity is represented as nanomoles of ethylene produced per minute and assay (left *y* axis). Error bars represent means ± SD (*n* = 3: NifB*, NifB^F^, and NifB^USF^; *n* = 4: NifB^US^). The shapes of symbols for each yeast strain indicate whether *M. infernus* NifB was purified from cells originating from the same or different fermenters. Average Fe content of each NifB is indicated with a black thick line (right *y* axis; *SI Appendix*, Table S5). (*B*) Titration of in vitro FeMo-co synthesis and apo-NifDK reconstitution using purified proteins (see *Materials and Methods* for details) and as-isolated *M. infernus* NifB^USF^ (purification 13; *SI Appendix*, Table S5). Activity is represented as nanomoles of ethylene produced per minute and milligram of NifDK. Error bars represent means ± SD (*n* = 2). Specific activities of holo-NifDK and NifB-codependent activated apo-NifDK determined under the same reaction conditions were, respectively, 1,331 and 260 nmol of ethylene formed per minute and milligram of NifDK protein. (*C*) Requirement of SAM for *M. infernus* NifB^USF^-dependent in vitro FeMo-co synthesis and apo-NifDK reconstitution in a completely defined assay; 5 μM *M*. *infernus* NifB^USF^ was used per assay (purification 13; *SI Appendix*, Table S5). Activities are compared to complete conditions (see *Materials and Methods* for details) and reported as nanomoles of ethylene produced per minute and milligram of apo-NifDK. Error bars represent means ± SD (*n* = 2). Specific activities of holo-NifDK and NifB-codependent activated apo-NifDK determined under the same reaction conditions were, respectively, 1,137 and 202 nmol of ethylene formed per minute and milligram of NifDK protein. (*D*) As-isolated and air-exposed UV-visible spectra of *M. thermautotrophicus* NifB^USF^ protein. (*E* and *F*) Appearance (*E*) and Coomassie staining (*F*) of *M. thermautotrophicus* NifB^USF^ obtained from 55 g of yeast cells following biotin-elution, desalting, and concentration. (*G*) *M. thermautotrophicus* NifB^USF^-dependent in vitro FeMo-co synthesis and apo-NifDK reconstitution using purified proteins (see *Materials and Methods* for details); 5 μM as-isolated *M. thermautotrophicus* NifB^USF^ was used in the assay. Error bars represent means ± SD (*n* = 2). Specific activities of holo-NifDK and NifB-codependent activated apo-NifDK determined under the same reaction conditions were, respectively, 1,314 and 334 nmol of ethylene formed per minute and milligram of NifDK protein.

The abilities of various NifB species to support FeMo-co synthesis was tested using the above-described in vitro FeMo-co synthesis and insertion assay (5). As-isolated *M. infernus* NifB* had no ability to support in vitro FeMo-co synthesis, and NifB^F^ and NifB^US^ had only minimal capacity relative to NifB^USF^ (Fig. 4*A*). In vitro FeMo-co synthesis performed in reactions containing only purified Nif components (*SI Appendix*, Fig. S19) (5) confirmed that as-isolated *M. infernus* NifB^USF^ could support FeMo-co formation without requiring prior [Fe-S] cluster reconstitution. A dose-dependent increase in apo-NifDK activation was observed in this system when NifB^USF^ was used as the limiting component in activation (Fig. 4*B*). These experiments established that NifB^USF^ can provide NifB-co to support FeMo-co formation in the in vitro system. However, they did not establish whether NifB-co was already present within the as-isolated NifB^USF^, or if SAM was required to convert a fraction of [4Fe-4S] clusters contained within as-isolated NifB^USF^ to NifB-co. This question was resolved by showing that NifB-co formation and apo-NifDK activation required both NifB^USF^ and SAM (Fig. 4*C* and *SI Appendix*, Fig. S20), demonstrating that preformed NifB-co was not contained within as-isolated NifB.

*M. thermautotrophicus* NifB^USF^ was also purified with [Fe-S] clusters and supported in vitro formation of FeMo-co and apo-NifDK activation (Fig. 4 *D*–*G* and *SI Appendix*, Fig. S21). *M. thermautotrophicus* NifB^USF^ purifications had higher yields than those of *M. infernus* NifB^USF^ (average, 17.9 mg of NifB^USF^ per 100 g of cells; *n* = 2), while the Fe content was slightly lower (average, 4 Fe atoms per NifB^USF^ monomer; *n* = 2) (*SI Appendix*, Table S5).

### Isolation of NifX with Bound FeMo-co Precursor Produced in Yeast.

Both *M. thermautotrophicus* and *M. infernus* NifB proteins lack the C-terminal NifX-like domain, and it is possible that they are incapable of accumulating NifB-co. As NifX has demonstrated affinity for NifB-co, it was built into the pathways to trap any in vivo-formed NifB-co. To overcome the instability of GST-NifX observed in the library screening, GST was replaced by a TS-tag–tobacco etch virus (TEV) site cassette (*SI Appendix*, Fig. S22 and *Supplementary Text* sequences). In addition, as accumulation of *M. thermautotrophicus* and *M. infernus* NifB proteins in yeast was very high, endogenous mitochondria SAM levels could be limiting. A mitochondria-targeted variant of the cytosolic SAM synthase (Sam1p) was therefore engineered following the strategy used by Marobbio et al. (32) (*SI Appendix*, Fig. S22 and *Supplementary Text* sequences). This approach is similar to the overexpression in *E. coli* of the SAM synthase gene *metK* used to obtain functional *M. infernus* NifB (12). Functionality of mitochondria-targeted Sam1p was verified by growth of *sam5*Δ cells transformed with SU9-Sam1p-FLAG in yeast extract–peptone media containing a nonfermentable carbon source (32) (*SI Appendix*, Fig. S23).

**Fig. 5.**
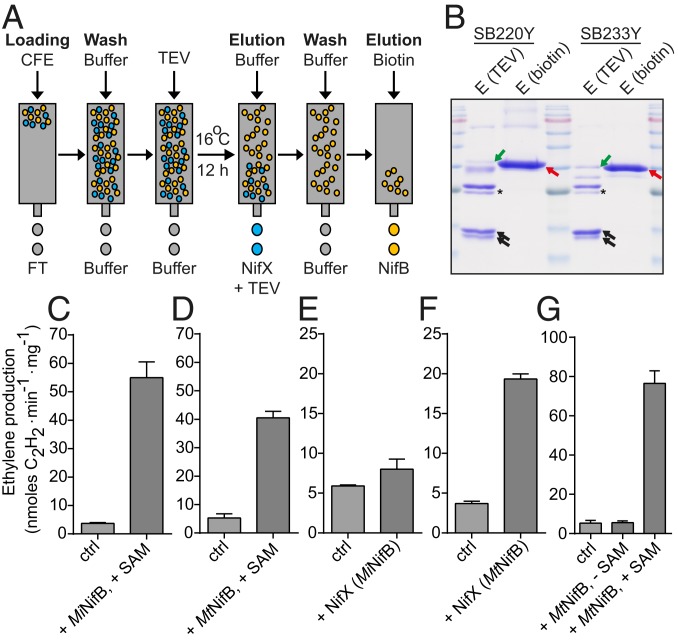
NifB- and NifX-dependent FeMo-co synthesis using proteins isolated from *S. cerevisiae* strains SB220Y (expressing NifX and *M. infernus* NifB^USF+SAM^) and SB233Y (expressing NifX and *M. thermautotrophicus* NifB^USF+SAM^). (*A*) Purification strategy to isolate NifX and NifB proteins from SB220Y and SB233Y. (*B*) Coomassie staining showing the appearance of the NifX and NifB proteins in the TEV and biotin-elution fractions, respectively. NifX migrates as a double band (black arrows). TEV is marked by black stars. NifB proteins are marked by red arrows. Some NifB proteins were also present in the TEV elution fraction (green arrows). Detailed analysis of the purification procedure is shown in *SI Appendix*, Fig. S24 *B* and *C*. (*C*–*G*) In vitro synthesis of FeMo-co and apo-NifDK reconstitution using purified protein components (see *Materials and Methods* for details). Activity is reported as nanomoles of ethylene produced per minute and milligram of NifDK. Error bars represent means ± SD (*n* = 2). For NifB-dependent FeMo-co synthesis (*C* and *D*), 5 μM as-isolated *M. infernus* NifB^USF+SAM^ (*C*) or *M. thermautotrophicus* NifB^USF+SAM^ (*D*) was used. For NifX-dependent FeMo-co synthesis in the absence of SAM, 5 μM NifX purified from SB220Y (*M. infernus* NifB^USF+SAM^) (*E*) or 9 μM NifX purified from SB233Y (*M. thermautotrophicus* NifB^USF+SAM^) (*F*) was used. To test NifB-dependent FeMo-co synthesis in the absence of SAM, 10 μM *M. thermautotrophicus* NifB^USF+SAM^ was used (*G*). Control reactions (ctrl) were performed in the absence of NifB (*C*, *D*, and *G*) or NifX (*E* and *F*). Specific activities of holo-NifDK and NifB-codependent activated apo-NifDK determined under the same reaction conditions were, respectively, 1,232 and 285 nmol of ethylene (*C* and *F*), 1,148 and 417 nmol of ethylene (*D* and *G*), and 1,014 and 343 nmol of ethylene (*E*) formed per minute and milligram of NifDK protein.

NifB and NifX proteins were purified simultaneously from mitochondria of *S. cerevisiae* strains additionally coexpressing NifU, NifS, FdxN, and Sam1p (Fig. 5 *A* and *B* and *SI Appendix*, Fig. S24). Both *M. infernus* NifB^USF+SAM^ and *M. thermautotrophicus* NifB^USF+SAM^ supported SAM-dependent FeMo-co formation and apo-NifDK reconstitution using purified protein components (Fig. 5 *C* and *D*). As NifX does not possess catalytic activity but acts as carrier of NifB-co, synthesis of FeMo-co and apo-NifDK reconstitution using NifX/NifB-co complex does not require SAM. We therefore performed SAM-independent FeMo-co synthesis assays using NifX purified from yeast expressing *M. infernus* NifB^USF+SAM^ or *M. thermautotrophicus* NifB^USF+SAM^. No substantial NifDK activity was measured with NifX purified from yeast expressing *M. infernus* NifB^USF+SAM^ (Fig. 5*E*). On the contrary, NifX purified from yeast expressing *M. thermautotrophicus* NifB^USF+SAM^ generated significant NifDK reconstitution (Fig. 5*F*), implying that NifX preparations contained bound NifB-co and that mitochondrial *M. thermautotrophicus* NifB^USF+SAM^ was active in vivo. Some NifB^USF+SAM^ contamination was observed in purified NifX fractions (Fig. 5*B*), but its role in SAM-independent FeMo-co synthesis was ruled out by demonstrated lack of activity of pure *M. thermautotrophicus* NifB^USF+SAM^ in the in vitro FeMo-co synthesis assay (Fig. 5*G*).

## Discussion

The first report of successful expression of functional NifH (dinitrogenase reductase) in aerobically grown *S. cerevisiae* established that mitochondria provide a suitable environment for production of the O_2_-sensitive nitrogenase proteins (16). The study also showed that activation of mitochondrial-targeted NifH only required additional coexpression of its associated maturase NifM. Thus, endogenous yeast mitochondrial [Fe-S] cluster biosynthetic machinery sufficed to provide NifH with its essential [4Fe-4S] cluster, which is normally provided by NifU and NifS (33). This result suggested that not all *nif* gene products essential for functional assembly of an active nitrogenase in a model prokaryotic system would necessarily be required for assembly of an active nitrogenase in a particular eukaryotic system. In other words, certain essential prokaryotic components can be replaced by eukaryotic proteins having similar functions. However, as discussed below, the present work reveals that this conclusion is not necessarily correct in the case of NifU and NifS, as they are required for formation of active NifB. Following the concept of system simplification and gene transfer reduction, a synthetic biology approach has been applied to establish that ferredoxin-NADPH oxidoreductases and ferredoxins of plant organelle origin can serve as electron sources to support nitrogenase catalysis (21). In another study, synthetic gene fusions and posttranslational processing enabled the regrouping of the 14 *Klebsiella oxytoca* genes required for heterologous expression of an active nitrogenase in *E. coli* into only 5 “giant” genes (23). Both strategies are excellent examples of how synthetic biology can be employed to simplify the challenge of endowing eukaryotic organisms with the capacity to reduce N_2_ (22, 24).

Taking into consideration recent progress in overcoming the anticipated problem of O_2_ sensitivity of nitrogenase components by targeting them to mitochondria, as well as the potential of using synthetic biology to reduce the genetic complexity of the system, in vivo formation of the nitrogenase active-site cofactor remains as one of the most significant obstacles to producing an active nitrogenase in eukaryotes. Given that NifB-co is a common precursor required for assembly of the corresponding active-site cofactors contained in all 3 nitrogenase types, formation of active NifB is critical to endowing any eukaryote with the capacity for N_2_ reduction. By employing synthetic biology to simultaneously test multiple factors influencing NifB function, the present work shows that a modified archaeal NifB variant expressed in aerobic yeast cultures, in combination with NifU, NifS, and FdxN accessory proteins from a diazotrophic Proteobacterium, is active without a requirement for further in vitro reconstitution. Expression and solubility levels of the different NifB variants tested here could not be anticipated. For example, although the NifB originating from *Gloeothece sp. (KO68DGA)* and *Cyanothece sp. (ATCC 51142)* showed more than 95% identity, NifB accumulation was only detected for the *Gloeothece sp. (KO68DGA)* protein. Notably, some NifB variants were detected only when expressed at high levels, while others worked only at low levels. Moreover, it was not known a priori that for accessory genes, some combinations of expression levels would result in complete abrogation of NifB expression. In addition, a surprisingly small number of NifB proteins were soluble. Although both *M. thermautotrophicus* and *M. infernus* NifB proteins were predicted to be stable (Dataset S1), expression of other NifB sequences with similar low instability index could not be detected, highlighting that NifB accumulation was not easily anticipated from sequence information or from prior observations. One important observation is that both *nifB* genes were sourced from extreme thermophiles, implying that such proteins may be more stable in this heterologous expression system. Overall, because a library-based approach revealed these kinds of dependencies, we anticipate that in the future, methods to facilitate simultaneous testing of multiple design factors will be important for the engineering of pathways of increasing complexity.

A requirement for NifU and NifS to produce active NifB was not expected because the maturation of [4Fe-4S]-containing NifH in yeast does not require NifU and NifS. Namely, for the assembly and delivery of the [4Fe-4S] cluster contained in yeast-expressed NifH, it appears that the function of NifU and NifS can be supplanted by the yeast-encoded scaffold (ISU) and cysteine desulfurase (NFS1). Why this is not the case for NifB [4Fe-4S] clusters could be related to the complexity of this enzyme containing 3 different [4Fe-4S] clusters compared to a single [4Fe-4S] in NifH.

It had been shown that a Δ*nifUS* mutation in *Klebsiella* *pneumoniae* decreased NifB levels by 10-fold and abolished NifB-co activity in extracts (13). By uncoupling *gst-nifB* expression from *nif* regulation, similar amounts of GST-NifB accumulated independently of the *nifUS* genetic background. Furthermore, GST-NifB produced by this method was replete with Fe-S species and could be isolated with bound NifB-co. Both GST-NifB variants (expressed in presence or absence of *nifUS*) could activate apo-NifDK to similar levels, although overall NifB-co levels were much lower in extracts of the Δ*nifUS* strain. This result contrasts with our results in *S. cerevisiae*, where NifU and NifS are required to produce NifB protein with high [Fe-S] cluster occupancy and no bound NifB-co was found. It should be noted that while NifB was produced in *K. pneumoniae* under strict anaerobic conditions, the *S. cerevisiae* strains used here were cultured under aerobic conditions. In addition, the different tags used for protein purification can influence metal content and activity, and the properties of the as-isolated proteins may not necessarily reflect the characteristics of the native protein. This could, for example, explain the different levels of NifX-bound NifB-co in *S. cerevisiae* strains carrying *nifB* from *M. infernus* or *M. thermautotrophicus*.

The involvement of FdxN in NifB maturation might also be relevant to understand the function of NifU and NifS. The U-type [Fe-S] proteins, of which NifU was the first member to be discovered (34), provide cysteine desulfurase-dependent scaffolds for the assembly of simple [Fe-S] clusters destined for maturation of other [Fe-S] cluster-containing proteins (33). In some cases, the U-type scaffold is not the direct donor to a particular client [Fe-S] protein but, instead, an intermediate carrier is involved. Although FdxN is annotated as a ferredoxin, its apparent involvement in supporting the acquisition of [Fe-S] clusters by NifB, rather than in SAM-dependent formation of NifB-co, could indicate a role as intermediate [4Fe-4S] carrier rather than as electron donor. In some cases, primary U-type scaffolds can function in the absence of the intermediate carrier, but only at a low level, and this could explain the very low level of NifB^US^-directed NifB-co formation and why FdxN is not strictly essential for *A. vinelandii* N_2_ fixation (15). Another possibility is that FdxN would function in either the reduction or protection against oxidation of cysteinyl residues in NifB involved in the coordination of a specific Fe-S cluster.

It should be noted that, based on elemental analysis and UV-visible and EPR spectroscopies, the *M. infernus* NifB^USF^ contained only about 60% of the potential [4Fe-4S] cluster occupancy. This suggests either a dynamic in vivo process wherein NifB-co is formed at the assembly site and subsequently released or that the availability of Fe and S (or the machinery providing [Fe-S] clusters) is insufficient. In other words, isolated NifB^USF^ is likely to represent a mixed population including one fraction replete with [4Fe-4S] clusters poised for NifB-co formation and another fraction that has released NifB-co but has not yet been recharged with the accessory clusters. Release of NifB-co from NifB upon its completion is supported by the observation that no NifB-co capable of supporting FeMo-cofactor formation in the absence of SAM was detected in as-isolated NifB^USF^.

It is intriguing that we could detect NifX-bound NifB-co from yeast expressing *M. thermautotrophicus* NifB^USF+SAM^ but not from yeast expressing *M. infernus* NifB^USF+SAM^. Like *M. infernus*, *M. thermautotrophicus* does not harbor an obvious *nifX* gene that could indicate that its NifB protein has evolved to interact with NifX. Instead, the difference in NifX-bound NifB-co could be explained by different NifB^USF+SAM^ expression, solubility or activity, or a combination of those. Another important question is whether NifB activity within mitochondria might be limited by the natural levels of SAM within that organelle. The *S. cerevisiae* SAM synthase was here engineered into a mitochondria-targeted variant because of the very high expression levels of *M. infernus* or *M. thermautotrophicus* NifB^USF^ proteins using the GAL-induced expression systems. The exact physiological parameters for SAM-dependent NifB activity when produced at lower levels in mitochondria will require further studies.

The present study leads to 5 important observations that are key to engineering a N_2_-fixing eukaryote: 1) an active form of NifB, required for the formation of the NifB-co precursor to the active-site cofactor of all nitrogenase types, can be produced in the mitochondria of a model eukaryotic organism such as *S. cerevisiae*; 2) NifB-co can be produced in the mitochondria of a eukaryotic cell grown under aerobic conditions; 3) factors such as FdxN, which are not necessarily essential for N_2_ fixation in prokaryotic cells, could be essential to produce a N_2_-fixing eukaryote; 4) genes and expression levels not necessary to produce active NifH in *S. cerevisiae*, such as NifU and NifS, are necessary to produce active NifB; and 5) complementary assembly factors produced by highly divergent bacteria can be combined and sorted to achieve the production of building blocks essential to formation of an active nitrogenase in eukaryotes.

## Materials and Methods

### Strains, Media, and Molecular Biology for Generation of Yeast Libraries.

*S. cerevisiae* CEN.PK113–7D (MATa URA3 TRP1 LEU2 HIS3 MAL2–8c SUC2) was the host strain for all library constructs and grown at 30 °C in yeast extract–peptone–dextrose media, with 200 μg/mL G418 added when appropriate. Yeast transformations were carried out according to the lithium acetate method (35, 36). Chemically competent *E. coli* DH5α (New England Biolabs) was used as a cloning strain and grown at 37 °C in lysogeny broth media with appropriate antibiotics (100 μg/mL carbenicillin or 25 μg/mL kanamycin) and inducer (100 μL of 40 mg/mL 5-bromo-4-chloro-3-indolyl-β-d-galactopyranoside) was spread and dried on plates for blue/white screening when appropriate.

All Sanger sequencing reactions were performed by Quintara Biosciences. Plasmid isolations were performed with Qiagen Qiaprep kits. Genomic DNA was isolated using the Promega Wizard Genomic DNA Preparation Kit. Gel electrophoresis was carried out using 1% agarose E-Gels according to the manufacturer’s instructions (Invitrogen). BsaI was purchased from New England Biolabs. BbsI was purchased from Thermo Fisher Scientific. High concentration T4 DNA ligase was purchased from Promega. All PCR primers were ordered from Integrated DNA Technologies. All PCRs used Q5 2× Master Mix from New England Biolabs. PCRs were performed on Eppendorf thermocyclers.

### Library Assembly.

Genes encoding *nifB* genes, SU9, and TS-tag were designed for expression in *S. cerevisiae* using the GeneOptimizer tool (ThermoFisher) and synthesized by ThermoFisher via the Engineering Nitrogen Symbiosis for Africa project. Other sequences have been described previously (25, 26). Once a design was defined, parts were cloned using a hierarchical Type IIS assembly strategy. Synthesized parts were PCR-amplified to add appropriate scars and were cloned into an ampicillin-resistant level-0 vector with BpiI (no. ER1012; ThermoFisher) and high-concentration T4 ligase (no. M1794; Promega). Each assembled part vector contained the part flanked by BsaI sites and 4-base pair (bp) overhangs. Each gene has a unique SU9 sequence that was added into its level-0 plasmid upstream of the gene. While the peptide sequence of SU9 was maintained, the DNA sequence of the mitochondrial tag was varied in order to minimize risk of undesired recombination. An N-terminal GST-tag was added to NifX, and all NifB variants had an N-terminal 2× Strep-tag (TS-tag) added. To assemble each level-1 transcription unit, 3 level-0 vectors containing either a promoter, gene, or terminator were combined with a level-1 destination vector, BsaI (no. R0535L; New England Biolabs), and high-concentration T4 ligase. Level-1 destination vectors were position-specific yeast-integrative plasmids with 50 bp of homologous DNA sequence to facilitate homologous recombination. The integrative yeast plasmids have the lethal *ccdb* gene for selection of clones and kanamycin resistance for *E. coli* selection. All assemblies were transformed into *E. coli*. Plasmid extraction was performed using a Qiaprep kit (no. 27106; Qiagen). Vectors were verified by Sanger sequencing (Quintara Biosciences).

For integration, NifS was cloned into pDSM-5a, NifU was cloned into pDSM-ab, FdxN was cloned into pDSM-cd, NifX was cloned into pDSM-de, and NifBs were cloned into pDSM-ef. Six combinations of parental strains were created with varying promoters and terminators to vary expression levels as shown (Fig. 2*A* and *SI Appendix*, Fig. S1 *A* and *B* and Tables S1 and S2). To construct the library of NifB variants, promoters Ptef1 and PPXR1 from *S. cerevisiae* were used for the high- and low-expression levels, respectively, and both used terminator TKIURA3 from *Kluyveromyces lactis.* A kanamycin-resistance gene was cloned into pDSM-bc with the strong promoter Pagtef1 and the TagTEFI terminator, both from *Ashbya gossypii*. These transcription units can recombine and integrate into yeast chromosome XV between NRT1 and GYP1 (starting at 459,247 bp and ending at 458,821 bp). Integrations were performed using PCR-amplified transcription units (Q5 no. M0492S; New England Biolabs) from the pDSM vectors, as well as PCR-amplified bridging DNA derived from the yeast genome. Parts were introduced to yeast by homologous recombination using the lithium acetate transformation method (35, 36). Integrations were verified by whole-genome DNA extraction (Wizard no. A1125; Promega), PCR, and gel electrophoresis (1% agarose E-gel).

### Preparation of Yeast Anaerobic Cell-Free Extracts and NifB Purifications.

*S. cerevisiae* cells expressing SU9-TS-NifB were resuspended in anaerobic buffer A—100 mM tris(hydroxymethyl)aminomethane (Tris)⋅HCl (pH 8.8), 300 mM NaCl, 10% glycerol—supplemented with 2 mM dithionite (DTH), 5 mM β-mercaptoethanol (β-ME), 1 mM phenylmethylsulfonyl fluoride, 1 μg/mL leupeptin, and 5 μg/mL DNase I. The cells were lysed in an Emulsiflex-C5 homogenizer (Avestin Inc.) at 25,000 pounds per square inch. Cell-free extracts (CFEs) were obtained by removal of cell debris and precipitated yeast proteins by centrifugation (50,000 × *g* for 1 h at 4 °C) and filtration through a 0.2-μm pore-size filter (Nalgene Rapid-Flow; Thermo Scientific). All procedures were performed under anaerobic conditions.

TS-NifB was purified by Strep-tag–binding chromatography using a 5-mL Strep-Tactin XT Superflow Cartridge (IBA Lifesciences) under anaerobic conditions (<0.1 ppm of O_2_) using an AKTA Prime fast protein liquid chromatography system (GE Healthcare) inside a glovebox (MBraun). All buffers were previously made anaerobic by sparging with N_2_. Before loading the CFE, the Strep-Tactin column was equilibrated with buffer B (100 mM Tris⋅HCl [pH 8.0], 300 mM NaCl, 10% glycerol, 2 mM DTH, 5 mM β-ME). A pH above 7.5 of the CFE was ensured before loading. Typically, anaerobic CFE from 100 g of cell paste was loaded at 2 mL/min and washed with 5 successive washes of 15 mL of buffer B. Bound protein was typically eluted with 12 mL of buffer B supplemented with 50 mM biotin and desalted using a HiPrep 26/10 Desalting column (GE Healthcare) equilibrated with buffer C (50 mM Tris⋅HCl [pH 8.0], 300 mM NaCl, 10% glycerol, 5 mM β-ME). Purity of the NifB protein was verified by SDS/PAGE and Coomassie staining, concentrated using a 10-kDa cutoff pore centrifugal membrane device (Amicon Ultra-15; Millipore), and analyzed by UV-visible spectroscopy. Finally, pure TS-NifB was supplemented with 2 mM DTH, frozen, and stored in liquid N_2_.

### In Vitro Synthesis of FeMo-co and Apo-NifDK Reconstitution Assay Using UW140 CFEs.

Assays were performed as described by Curatti et al. (6), with slight modifications. Reactions were prepared inside a glovebox (CoyLabs) using 9-mL serum vials previously washed with 1 mL of anaerobic buffer. The in vitro FeMo-co synthesis and insertion reactions were performed in 400-μL total volume that included 50 μL of reaction buffer (25 mM Tris⋅HCl [pH 7.8], 17.5 μM Na_2_MoO_4_, 175 μM *R*-homocitrate, 880 μM SAM, 3 mM DTH), 100 μL of adenosine triphosphate (ATP) mix (3.6 mM ATP, 59 mM phosphocreatine disodium salt, 7.5 mM MgCl_2_, 7.5 mM DTH, 500 μg/mL creatine phosphokinase), and 200 μL of UW140 (*A. vinelandii ΔnifB*) CFE at 14.64 mg/mL total protein concentration supplemented with 2 μM NifH. Finally, 50 μL of buffer (25 mM Tris⋅HCl [pH 7.8]), or buffer supplemented with NifB (12.5 μM final concentration), or purified NifB-co as positive-control reaction (10 μM Fe final concentration) was added. The N_2_ atmosphere was changed to argon (Ar), and vials were incubated for FeMo-co synthesis and insertion at 30 °C for 35 min.

Following in vitro synthesis of FeMo-co, activation of apo-NifDK present in the UW140 extract was analyzed following addition of excess NifH and ATP-regenerating mixture (total volume, 0.8 mL) by acetylene reduction assay at 30 °C for 15 min following standard procedures (37). Positive-control reactions included *A. vinelandii* DJ (wild-type) CFE, *A. vinelandii* UW140 CFE complemented with NifB-co purified from *K. pneumoniae* strain UC32 (UN1217, *nifN*::mu, *Ptac*::*gst*-*nifX*) (8), or [Fe-S] cluster reconstituted yeast NifB_*Mi*_-His_10_ (17).

### In Vitro FeMo-co Synthesis and Insertion Assays in Defined System Using Purified Proteins.

Assays were performed as described by Curatti et al. (5), with slight modifications. Unless specified, NifB-dependent FeMo-co synthesis assays were performed in 100-μL reactions containing 17.5 μM Na_2_MoO_4_, 175 μM *R*-homocitrate, 125 μM FeSO_4_, 125 μM Na_2_S, 125 μM SAM, 1.23 mM ATP, 18 mM phosphocreatine disodium salt, 2.2 mM MgCl_2_, 3 mM DTH, 40 μg/mL creatine phosphokinase, 5.0 μM NifB, 3.0 μM NifX, 1.5 μM apo-NifEN, 3.0 μM NifH, 0.6 μM apo-NifDK, and 1 mg/mL bovine serum albumin in 22 mM Tris⋅HCl buffer (pH 7.5). FeMo-co synthesis and insertion into apo-NifDK was performed under N_2_ atmosphere at 30 °C for 45 min. NifX-dependent FeMo-co synthesis was performed in the absence of NifB and SAM, and with NifX isolated from yeast replacing the *A. vinelandii* NifX in the above reaction.

Following in vitro synthesis of FeMo-co, activation of apo-NifDK was analyzed by addition of 500 μL of 2.0 μM NifH and ATP-regenerating mixture (1.23 mM ATP, 18 mM phosphocreatine disodium salt, 2.2 mM MgCl_2_, 3 mM DTH, 40 μg/mL creatine phosphokinase, final concentrations in 22 mM Tris⋅HCl [pH 7.5] buffer) in 9-mL vials under Ar atmosphere. Acetylene reduction assays were performed at 30 °C for 15 min following standard procedures (37). Positive-control reactions for acetylene reduction were carried out with pure preparations of *A. vinelandii* holo-NifDK or apo-NifDK activated using precursor-deficient apo-NifEN supplemented with purified NifB-co (25 μM Fe final concentration) (8). The purification of other proteins used in the assay has been previously described (14).

### EPR Analysis of NifB.

NifB preparations in 50 mM Tris⋅HCl (pH 8.0), 300 mM NaCl, 10% glycerol, 2 mM DTH, and 5 mM β-ME were prepared for EPR analysis. X-band (9.64 GHz) EPR spectra were recorded on a Bruker E500A spectrometer equipped with an Oxford ESR 910 cryostat for low-temperature measurements. The microwave frequency was calibrated with a frequency counter and the magnetic field with an NMR gauss meter. The temperature of the X-band cryostat was calibrated with a carbon-glass resistor temperature probe (CGR-1-1000; LakeShore Cryotronics). For all EPR spectra, a modulation frequency and amplitude of 100 kHz and 1 mT were used. The EPR spectra of Fig. 3*E* and *SI Appendix*, Fig. S14 were recorded at 12 K. EPR spectral simulations were performed using the simulation software Spin Count (38); 1 mM Cu(II)ethylenediaminetetraacetic solution is used as spin standard for spin quantification. Two EPR samples independently prepared from 2 different purifications, NifB^US^ and NifB^USF^, were measured. Both purifications yielded very similar EPR signals. One set of data is presented in Fig. 3*E*, and both sets are presented in *SI Appendix*, Fig. S12.

### Data and Materials Availability.

All data are available in the main text, *SI Appendix*, or Dataset S1.

## Supplementary Material

Supplementary File

Supplementary File
