# Supplementary material for: Biosynthesis of the nitrogenase active-site cofactor precursor NifB-co in Saccharomyces cerevisiae
Source: Proc Natl Acad Sci U S A. 2019 Nov 25;116(50):25078–86. doi: 10.1073/pnas.1904903116 (PMC6911178; doi:10.1073/pnas.1904903116)
Supplement: Supplementary File [file pnas.1904903116.sapp.pdf]

Supplementary Information for

Title
Biosynthesis of the nitrogenase active-site cofactor precursor NifB-co in *Saccharomyces cerevisiae*

Short title
Functional NifB in *Saccharomyces cerevisiae*

Author Affiliation
Stefan Burén[a], Katelin Pratt[b], Xi Jiang[a], Yisong Guo[c], Emilio Jimenez-Vicente[d], Carlos Echavarri-Erasun[a,e], Dennis R. Dean[d], Ishtiaq Saaem[f,b], D. Benjamin Gordon[f,b], Christopher A. Voigt[f], and Luis M. Rubio[a,e]

[a]Centro de Biotecnología y Genómica de Plantas, Universidad Politécnica de Madrid, Instituto Nacional de Investigación y Tecnología Agraria y Alimentaria, Pozuelo de Alarcón, 28223 Madrid, Spain; [b]Broad Institute of MIT and Harvard, Cambridge, MA 02142; [c]Department of Chemistry, Carnegie Mellon University, Pittsburgh, PA 15213; [d]Department of Biochemistry, Virginia Polytechnic Institute, Blacksburg, VA 24061; [e]Departamento de Biotecnología-Biología Vegetal, Escuela Técnica Superior de Ingeniería Agronómica, Alimentaría y de Biosistemas, Universidad Politécnica de Madrid, 28040 Madrid, Spain; and [f]Synthetic Biology Center, Department of Biological Engineering, Massachusetts Institute of Technology, Cambridge, MA 02139

Author ORCID number
Stefan Burén (0000-0002-8487-2732), Katelin Pratt, Xi Jiang (0000-0002-1819-9041), Yisong Guo (0000-0002-4132-3565), Emilio Jimenez-Vicente (0000-0002-3347-3096), Carlos Echavarri-Erasun (0000-0001-8363-5199), Dennis R. Dean (0000-0001-8960-6196), Ishtiaq Saaem, D. Benjamin Gordon (0000-0003-0619-7791), Christopher A. Voigt (0000-0003-0844-4776), and Luis M. Rubio (0000-0003-1596-2475).

Corresponding Authors
Luis M. Rubio (ORCID 0000-0003-1596-2475)
Centro de Biotecnología y Genómica de Plantas, Universidad Politécnica de Madrid, Instituto Nacional de Investigación y Tecnología Agraria y Alimentaria, Pozuelo de Alarcón, 28223 Madrid, Spain.
+34 914 52 49 00
lm.rubio@upm.es

Christopher A. Voigt (0000-0003-0844-4776)
Synthetic Biology Center, Department of Biological Engineering, Massachusetts Institute of Technology, Cambridge, MA 02139, United States.
+1 617 324 4851
cavoigt@gmail.com

www.pnas.org/cgi/doi/10.1073/pnas.1904903116

This file includes:

Supplementary Materials and Methods
Supplementary Text
Supplementary Figures
Supplementary Tables
Supplementary Dataset
Supplementary References

Supplementary Materials and Methods

Generation of plasmids for galactose-induced yeast expression. *E. coli* DH5α was used for storage and amplification of yeast expression pESC vectors (Agilent Technologies). *E .coli* was grown at 37°C in Luria-Bertani (LB) medium supplemented with appropriate antibiotics. pN2GLT4 for yeast codon-optimized expression of mitochondria targeted *A. vinelandii* NifU ($NifU_{Av}$) and NifS ($NifS_{Av}$) has been previously described (1).

To transfer *su9-twinstrep-nifB* candidate genes to pESC vectors for GAL induced expression, genes were amplified from *S. cerevisiae* genomic DNA using primers 5´-AGTCGGATCCATGGCCTCCACTCGTGTCC and 5´-AAAGGTACCTATTAAGTTTCCTGTATATTTAGTCACC. Amplified DNA fragments were digested with BamHI/KpnI, and inserted into similarly digested pESC-TRP already containing the sequence coding for SU9-FdxN-HA, generating pN2SB99, pN2SB100, pN2SB101, pN2SB102, pN2SB103 and pN2SB104.

Sequence coding for *su9-twinstrep-nifB*$_{Mi}$ was created by overlapping PCR reactions as specified below, and cloned into pN2SB22 (1) using standard techniques. Primers used for generating *su9-twinstrep-nifB*$_{Mi}$ were 5'- AAAAAGATCTATGGCCTCCACTCGTGT-3' and 5'-TTTTCCATGGATCCTTCGAGTTTTTCAAATTGTGGATGTG-3' (using GenScript generated *su9-twinstrep* fragment as template), 5'-TTGAAAAACTCGAAGGATCCATGGAAAAGATGTCCAA-3' and 5'-TTTTTCTCGAGGTCACCTCAATGAGA-3' (using ThermoFisher generated *nifB*$_{Mi}$ fragment as template), to amplify *su9-twinstrep* and *nifB*$_{Mi}$, respectively. 5'-AAAAAGATCTATGGCCTCCACTCGTGT-3' and 5'-TTTTTCTCGAGGTCACCTCAATGAGA-3' were used for the overlapping PCR reaction. *su9-twinstrep-nifB*$_{Mi}$ was digested with BglII/XhoI and cloned into pN2SB22 (1) digested with BamHI/XhoI, replacing *su9-nifB*$_{Av}$*-His*$_{10}$ and generating pN2SB44. *su9-twinstrep-nifB*$_{Mi}$ was cloned into pESC-LEU using AgeI/NheI, generating pN2XJ154. *su9-twinstrep-nifB*$_{Mi}$ was cloned into pN2SB39 (1) using AgeI/NheI, replacing *su9-nifB*$_{Av}$*-His*$_{10}$ and generating pN2XJ155. Strep-tagged *M. infernus* NifB expressed from pN2XJ155 is four amino acids shorter than the corresponding protein expressed from pN2SB101 that contains an additional L-E-G-S peptide between the TwinStrep sequence and the start of the NifB protein (see Supplementary Text, DNA and amino acid sequences, for further details).

Sequence coding for *su9-sam1p* was created by overlapping PCR reactions as specified below. The sequence encoding *su9* was amplified using 5´-AAAGCGGCCGCATGGCCTCCACTCGTGTCCT-3´ and 5´-AATAAAAATGTACCGGCCATCGAAGAGTAGGCGCGCTTCTGGA-3´ (using GenScript generated *su9* fragment as template). The gene encoding SAM1 (YLR180W) was amplified from genomic *S. cerevisiae* DNA (strain CEN.PK2-1C) using 5´-AAGCGCGCCTACTCTTCGATGGCCGGTACATTTTTATTCACTT-3´ and 5´-TTTACTAGTGCGAACTTCAAAGTCTTAGGCTTTTCCCAT-3´. Primers 5´-AAAGCGGCCGCATGGCCTCCACTCGTGTCCT-3´ and 5´-TTTACTAGTGCGAACTTCAAAGTCTTAGGCTTTTCCCAT-3´ were used for the overlapping PCR. *su9-sam1p* was cloned into pESC-LEU using NotI/SpeI, creating *su9-sam1p-flag*. The sequence coding for *su9-twinstrep-tev-nifX* was created by overlapping PCR reactions as specified below. The sequence encoding *su9-twinstrep* was amplified from pN2XJ155 using 5´-AGTCGGATCCATGGCCTCCACTCGTGTCC-3´ and 5´-AGGTTCTCGCCGCCTCCTTCGAGTTTTTCAAATTGT-3´. The sequence encoding *tev-nifX*

was amplified using 5´-AAAAACTCGAAGGAGGCGGCGAGAACCTGTA-3´ and 5´-TTTTTGCTAGCTCACTCATCCCATCCCT-3´ (using GenScript generated *su9* fragment as template). Primers 5´-AGTCGGATCCATGGCCTCCACTCGTGTCC-3´ and 5´-TTTTTGCTAGCTCACTCATCCCATCCCT-3´ were used for the overlapping PCR. *su9-twinstrep-tev-nifX* was cloned into pESC-LEU containing *su9-sam1p-flag* using BamHI/NheI, creating pN2SB97. The sequence encoding *sam1p-flag* was amplified from pN2SB97 using 5´-AAAGCGGCCGCATGGCCGGTACATTTTTATTCACTT-3´ and 5´-GGCAAGGTAGACAAGCCGACAAC-3´. *sam1p-flag* was cloned into pN2SB97 using NotI/SpeI, replacing *su9-sam1p-flag* and generating pN2SB106.

Generation of yeast strains, growth, protein expression, and mitochondria isolations. *S. cerevisiae* W303-1a (*MAT*a *leu2-3,112 trp1-1 can1-100 ura3-1 ade2-1 his3-11,15*) was the host strain for expression vectors pN2GLT4 and pN2SB44 (to generate strain SB17Y); pN2GLT4 and pN2SB45 (to generate strain SB18Y); pN2GLT4 and pN2SB46 (to generate strain SB19Y); pN2XJ154 (to generate strain SB30Y); pN2GLT4 and pN2XJ154 (to generate strain SB31Y); pN2XJ155 (to generate strain SB32Y); pN2GLT4 and pN2XJ155 (to generate strain SB33Y); pN2GLT4, pN2SB97 and pN2XJ155 (to generate strain SB220Y); pN2GLT4 and pN2SB99 (to generate strain SB222Y); pN2GLT4 and pN2SB100 (to generate strain SB223Y); pN2GLT4 and pN2SB101 (to generate strain SB224Y); pN2GLT4 and pN2SB102 (to generate strain SB225Y); pN2GLT4 and pN2SB103 (to generate strain SB226Y); pN2GLT4 and pN2SB104 (to generate strain SB227Y); and pN2GLT4, pN2SB97 and pN2SB103 (to generate strain SB233Y). *S. cerevisiae* BY4741 with deleted SAM5 (YNL003c) was provided by EUROSCARF (strain Y05331) and was the host strain for pGEV-His (2) (to generate strain SB254Y), pGEV-His and pN2SB106 (to generate strain SB255Y), and pGEV-His and pN2SB97 (to generate strain SB256Y). Yeast transformations, growth and galactose inductions for small-scale protein extracts was performed as previously described (1).

Total yeast protein extracts to verify protein expression were prepared in order to retain small proteins (3). Typically, cell pellets of 1-2 ml yeast culture were resuspended in 20 µl lysis buffer (100 mM NaOH, 50 mM EDTA, 2% SDS, 2% β-mercaptoethanol (β-ME), 1 mM phenyl-methylsulfonyl fluoride (PMSF)) per OD x ml culture and heated at 90ºC for 10 min. Subsequently, 10 µl 1 M acetic acid was added per 100 µl lysis buffer and samples were vortexed for 30 sec. Double concentrated Laemmli buffer (2xLB) was added and samples were again heated at 90ºC for 10 min. Finally, the supernatant following centrifugation using a bench-top centrifuge operating at full speed was analyzed by SDS-PAGE. Soluble yeast protein extraction to analyze NifB solubility was performed by mechanical disruption, or using a protein extraction reagent. Mechanical disruption (Fig. 2*E*, and *SI Appendix*, Fig. S4 and Fig. S5*C*) was performed by resuspending yeast cell pellets in buffer (100 mM Tris-HCl (pH 8.0), 400 mM NaCl, 10% glycerol, 1 mM PMSF, 5 mM bME). Cells were broken in 2 ml tubes using 0.5 mm glass beads (BioSpec Products) in a mixer mill (Retsch MM300) operating at 30 Hz in 5 cycles of 1 min at 4ºC. The supernatant after 20 min centrifugation at 20,000 x g and 4ºC containing soluble proteins was analyzed by SDS-PAGE and immunoblot analysis. Soluble protein extraction using the YeastBuster Protein Extraction Reagent (Novagen) (*SI Appendix*, Fig. S3*B* and Fig. S6) was performed following the manufacturers instruction. Shortly, yeast cells were centrifuged at 5.000 g for 3 min. Pellets were then resuspended in 9 ul YeastBuster per OD*ml yeast culture (supplemented with 1xTHP, 5 µg/ml DNAse I, 200 µg/ml lysozyme, and 1 mM PMSF) and incubated at 600 rpm for 20 min at room temperature. Samples were then centrifuged for 20 min

at 4ºC. The resulting supernatant containing soluble proteins was analyzed by SDS-PAGE and immunoblot analysis. Total yeast protein extracts of strain SB17Y were used to analyze temperature-dependent solubility of NifB as previously described (1). Mitochondria isolations were performed as previously described (4). Enrichment was verified using tubulin (cytoplasmic) and HSP60 (mitochondria) marker proteins. Similar loading on SDS-PAGE experiments was obtained by preparing samples according to optical density, and was confirmed by using either Commassie staining of polyacrylamide gels or Ponceau staining of nitrocellulose membranes. Additionally, immunoblotting with antibodies against tubulin or HSP60 were used as control of gel loading and sample precipitation.

Cultures for yeast expressed NifB purifications were grown in a 4 l fermenter (BIO-STAT) as previously reported (1), with the following modifications. For strains SB30Y-SB32Y (procedure 1), cultures were grown at 30ºC in selective SD-medium for 16 h, followed by 8 h in rich medium (0.25% yeast extract, 0.25% bactopeptone, 0.25% bactotryptone, 2.5% sucrose), supplemented with 25 mg/l ammonium iron(III) citrate, 0.225 mM iron (II) sulfate, 1.25 mM magnesium sulfate, 1.5 mM calcium chloride and trace element solution (5). Finally, protein expression was induced for 16 h by addition of 2.25% galactose, 0.25% bactopeptone, 0.25% bactotryptone and vitamin solution (5). The pH was automatically maintained around 5 using 0.8 M ammonium hydroxide. Air flow was maintained at 2.5 l air/min per 4 L culture, at 250 rpm. Dissolved oxygen dropped to zero (as measured by oxygen sensor, Mettler Toledo) before addition of galactose, and remained at zero during the rest of the process. For strains SB222Y-SB227Y (procedure 2), cultures were grown as for SB30Y-SB32Y, except that a 300 ml pre-inoculum grown in selective SD-medium was used to start a 4 L fermenter, with rich medium and galactose from the beginning, for 20 h. For constitutive Nif expression in strain SB187Y (procedure 3), a 4 L culture was grown as previously reported (6), but with glucose instead of galactose.

Growth of SU9-Samp1-FLAG-transformed *sam5*Δ and control cells was measured in YP media containing 3% glycerol as non-fermentable carbon source (7). *S. cerevisiae* wild-type BY4741, and BY4741 with deleted SAM5 (YNL003c, strain Y05331) were provided by EUROSCARF. GAL regulatory elements were induced using 0.1 μM β-estradiol in cells containing pGEV-His (2). Each strain was cultured in triplicates with a starting optical density (OD^{600}) of 0.10, and grown under aerobic conditions at 30ºC. Induction and protein expression was verified in total yeast protein extracts collected 75 h after the start of the experiment.

NifB pulldowns and activity screening assay.

S. cerevisiae strains for Strep-tactin pulldowns and screening of NifB activity were flask-cultured in standard YPD medium, at 30ºC under aerobic conditions. Soluble yeast protein extracts to analyze NifB activity were prepared in anaerobic YeastBuster Protein Extraction Reagent (Novagen). Shortly, yeast cells corresponding to 25 ml culture at OD=8 were centrifuged at 3.000 g for 10 min. Pellets were washed in 1 ml milli-Q water, centrifuged, and then frozen in liquid N_2 before introduced into an anaerobic glovebox (MBraun). Pellets were washed once in anaerobic buffer (100 mM Tris-HCl pH 8.0, 300 mM NaCl, 10% glycerol, 2 mM DTH, 5 mM β-ME), and then lysed in 1 ml anaerobic YeastBuster Protein Extraction Reagent (supplemented with 1xTHP, 2 mM DTH, 5 mM β-ME, 1 mM PMSF, 1 μg/ml leupeptin, and 5 μg/ml DNAse I). Following incubation for 20 min at room temperature and 1,000 rpm, samples were centrifuged for 5 min at maximum speed using benchtop centrifuge. The resulting supernatants containing soluble proteins were analyzed by SDS-PAGE and immunoblot analysis. 700 μl of the supernatant was added to 1.3 ml of the above described buffer containing Strep-tactin resin (IBA Lifesciences), and then

incubated for 2 h with gentle shaking inside the glovebox. Strep-tactin beads were washed once with 1 ml buffer, and finally eluted in 180 µl buffer containing 50 mM biotin (IBA Lifesciences). NifB enrichment in the supernatant was tested by SDS-PAGE and immunoblot analysis, and 75 ul (in duplicates) was used for FeMo-co synthesis and apo-NifDK reconstitution using UW140 cell-free extracts.

UV-visible spectroscopy, N-terminal sequencing, Western blotting and protein methods. As-isolated NifB preparations were used for colorimetric Fe determination (8), *in vitro* FeMo-co synthesis and nitrogenase activity assays (see sections below), and UV-visible spectroscopy. UV-visible absorption spectra were recorded under anaerobic conditions in septum-sealed cuvettes using a Shimadzu UV-2600 spectrophotometer. When indicated, anaerobic samples were exposed to air during 5 min. UV-visible absorption spectra were recorded against buffer C as baseline. Absorbance at 800 nm was subtracted and spectra were then normalized to 279 nm. The N-terminal amino acid sequence of purified NifB was determined by Edman degradation (Proteome Factory AG). Western blot signals were detected using X-ray films (AGFA), or using an iBright FL1000 Imaging System (ThermoFisher Scientific) with auto-contrast setting. Protein concentrations were measured using the BCA protein assay (PIERCE). Samples were pre-treated with iodoacetamide before performing the BCA assay to eliminate the interfering effect of DTH (9).

Antibodies. Antibodies used in this study and their dilutions for immunoblotting were as follows: polyclonal antibodies detecting $NifU_{Av}$ (used at 1:2,000 in 5% BSA), $NifS_{Av}$ (used at 1:1,000 in 5% BSA), $NifB_{Mi}$ (used at 1:2,000 in 5% BSA) were raised against purified preparations of the corresponding *A. vinelandii* or *M. infernus* proteins. HA-tag (3F10, 12013819001, Roche, 1:1,000 in 2% non-fat dry milk), Strep-tag II (StrepMAB-Classic, 2-1507-001, IBA Lifesciences, 1:2,000 in 5% BSA), Strep-Tactin conjutaged to HRP (2-1502-001, IBA Lifesciences, 1:50,000 in TBS-T), HSP60 (LK-2, ab59458, Abcam, 1:1,000 in 5% BSA), and Tubulin (3H3087, sc-69971, Santa Cruz Biotechnology, 1:500 in 5% BSA) specific antibodies are commercially available.

Supplementary Text

DNA and amino acid sequences

DNA sequences used for parental strains and NifB library:

SU9-**NifS**

ATGGCTTCCACTCGTGTCCTGGCCTCTCGCCTGGCCTCGCAGATGGCTGCTTCCGCCAAA

M A S T R V L A S R L A S Q M A A S A K

GTTGCCCGCCCTGCTGTCCGCGTTGCTCAGGTGAGCAAGCGCACCATCCAGACTGGCTCG

V A R P A V R V A Q V S K R T I Q T G S

CCCCTCCAGACCCTCAAGCGCACCCAGATGACTTCCATCGTCAACGCCACCACCCGCCAA

P L Q T L K R T Q M T S I V N A T T R Q

GCTTTCCAGAAGCGCGCCTACTCTTCGGCCGACGTTTACTTGGATAATAACGCTACTACA

A F Q K R A Y S S **A D V Y L D N N A T T**

AGAGTCGATGACGAAATAGTACAAGCTATGTTGCCATTTTTCACAGAACAATTCGGTAAC

R V D D E I V Q A M L P F F T E Q F G N

CCTTCCAGTTTGCATTCCTTCGGTAACCAAGTTGGTATGGCCTTGAAGAAAGCTAGACAA

P S S L H S F G N Q V G M A L K K A R Q

TCTGTCCAAAAATTGTTAGGTGCAGAACACGATTCCGAAATCGTTTTTACCAGTTGTGGT

S V Q K L L G A E H D S E I V F T S C G

ACTGAATCTGACTCAACCGCCATTTTGTCTGCCTTAAAAGCTCAACCAGAAAGAAAGACT

T E S D S T A I L S A L K A Q P E R K T

GTCATAACCACTGTTGTCGAACATCCTGCAGTATTGTCTTTATGCGATTATTTGGCCTCA

V I T T V V E H P A V L S L C D Y L A S

GAAGGTTACACTGTTCATAAGTTACCAGTCGATAAAAAGGGTAGATTGGACTTAGAACAC

E G Y T V H K L P V D K K G R L D L E H

TATGCTTCCTTGTTAACAGATGACGTAGCTGTAGTTAGTGTTATGTGGGCAAATAACGAA

Y A S L L T D D V A V V S V M W A N N E

ACTGGTACATTGTTTCCAATTGAAGAAATGGCAAGATTAGCCGATGACGCTGGTATAATG

T G T L F P I E E M A R L A D D A G I M

TTCCATACTGATGCAGTACAAGCCGTTGGTAAAGTCCCTATAGACTTGAAGAACTCGTCA

F H T D A V Q A V G K V P I D L K N S S

ATCCACATGTTGTCCTTAAGTGGTCATAAATTGCACGCTCCAAAGGGTGTTGGTGTCTTG

I H M L S L S G H K L H A P K G V G V L

TACTTAAGAAGAGGTACAAGATTCAGACCTTTGTTAAGAGGTGGTCATCAAGAAAGAGGT
Y L R R G T R F R P L L R G G H Q E R G

AGGAGAGCCGGTACTGAAAATGCTGCATCTATTATAGGTTTGGGTGTTGCCGCTGAAAGA
R R A G T E N A A S I I G L G V A A E R

GCTTTACAATTCATGGAACATGAAAACACTGAAGTTAAGAGATTGCGTGATAAGTTAGAA
A L Q F M E H E N T E V K R L R D K L E

GCAGGTATTTTGGCCGTCGTACCACACGCATTTGTTACTGGTGACCCAGACAATAGATTA
A G I L A V V P H A F V T G D P D N R L

CCTAACACAGCTAACATCGCATTCGAATACATCGAAGGTGAAGCTATCTTGTTGTTGTTG
P N T A N I A F E Y I E G E A I L L L L

AACAAAGTTGGTATAGCAGCCTCCAGTGGTTCTGCTTGTACATCTGGTTCATTGGAACCA
N K V G I A A S S G S A C T S G S L E P

TCACATGTTATGAGAGCAATGGATATTCCTTATACAGCTGCACACGGTACTGTTAGATTT
S H V M R A M D I P Y T A A H G T V R F

TCTTTGAGTAGATACACAACCGAAGAAGAAATTGATAGAGTCATTAGAGAAGTACCACCT
S L S R Y T T E E E I D R V I R E V P P

ATTGTTGCTCAATTGAGAAAATTGTCTCCTTACTGGTCAGGTAATGGTCCTGTTGAGGAC
I V A Q L R K L S P Y W S G N G P V E D

CCTGGTAAAGCCTTTGCTCCTGTCTATGGTTAA
P G K A F A P V Y G -

SU9-**NifU**

ATGGCCTCCACTAGAGTCCTCGCCTCTCGGCTGGCCTCCCAGATGGCCGCTTCCGCCAAG
M A S T R V L A S R L A S Q M A A S A K

GTAGCCCGCCCTGCTGTCCGCGTTGCCCAGGTCAGCAAGCGCACCATCCAAACTGGCTCC
V A R P A V R V A Q V S K R T I Q T G S

CCCCTCCAGACCCTCAAGCGTACCCAGATGACCTCCATCGTCAACGCCACCACTCGCCAG
P L Q T L K R T Q M T S I V N A T T R Q

GCTTTCCAGAAGCGCGCCTACTCTTCCTGGGACTACTCTGAAAAGGTTAAGGAACATTTC
A F Q K R A Y S S **W D Y S E K V K E H F**

TACAATCCAAAGAACGCCGGTGCTGTAGAAGGTGCAAACGCCATTGGTGACGTTGGTTCA
Y N P K N A G A V E G A N A I G D V G S

TTATCCTGTGGTGACGCTTTGAGATTAACATTGAAAGTTGACCCTGAAACCGATGTCATC
L S C G D A L R L T L K V D P E T D V I

TTGGACGCAGGTTTTCAAACTTTCGGTTGCGGTTCTGCTATTGCATCTTCATCCGCTTTG
L D A G F Q T F G C G S A I A S S S A L

ACTGAAATGGTTAAGGGTTTGACATTGGATGAAGCATTGAAAATCTCAAACCAAGATATC
T E M V K G L T L D E A L K I S N Q D I

GCTGACTATTTGGATGGTTTGCCACCTGAAAAGATGCATTGTTCCGTCATGGGTAGAGAA
A D Y L D G L P P E K M H C S V M G R E

GCCTTACAAGCTGCAGTAGCTAACTACAGAGGTGAAACCATTGAAGATGACCACGAAGAA
A L Q A A V A N Y R G E T I E D D H E E

GGTGCATTGATATGTAAATGCTTTGCCGTTGATGAAGTTATGGTCAGAGATACCATAAGA
G A L I C K C F A V D E V M V R D T I R

GCAAATAAGTTAAGTACTGTAGAAGATGTTACTAACTACACAAAAGCTGGTGGTGGTTGT
A N K L S T V E D V T N Y T K A G G G C

TCTGCTTGCCATGAAGCAATAGAAAGAGTTTTGACAGAAGAATTGGCCGCTAGAGGTGAA
S A C H E A I E R V L T E E L A A R G E

GTATTCGTTGCAGCCCCAATTAAAGCCAAAAAGAAAGTCAAGGTATTGGCTCCAGAACCT
V F V A A P I K A K K K V K V L A P E P

GCCCCAGCTCCTGTTGCAGAAGCCCCAGCTGCAGCCCCTAAGTTGTCAAATTTGCAAAGA
A P A P V A E A P A A A P K L S N L Q R

ATTAGAAGAATCGAAACAGTCTTGGCTGCAATAAGACCTACCTTGCAAAGAGACAAAGGT
I R R I E T V L A A I R P T L Q R D K G

GACGTCGAATTAATTGATGTAGACGGTAAAAATGTTTACGTCAAATTGACCGGTGCTTGT
D V E L I D V D G K N V Y V K L T G A C

ACTGGTTGCCAAATGGCATCCATGACATTAGGTGGTATACAACAAAGATTGATCGAAGAA
T G C Q M A S M T L G G I Q Q R L I E E

TTGGGTGAGTTCGTCAAAGTTATCCCAGTCTCCGCTGCCGCACACGCCCAAATGGAAGTC
L G E F V K V I P V S A A A H A Q M E V

TAA
-

SU9-**GST**-**NifX**

ATGGCTTCAACAAGGGTTTTGGCTTCAAGATTGGCTTCCCAGATGGCAGCTTCCGCTAAG
M A S T R V L A S R L A S Q M A A S A K

GTGGCTAGACCAGCAGTGAGAGTGGCACAAGTTTCAAAGAGAACTATTCAAACAGGTTCT
V A R P A V R V A Q V S K R T I Q T G S

CCATTACAAACTTTGAAGAGAACTCAAATGACATCAATCGTTAACGCTACTACAAGACAA
P L Q T L K R T Q M T S I V N A T T R Q

GCTTTTCAAAAGAGAGCTTACTCTTCAATGTCTCCAATCTTGGGTTACTGGAAAATTAAG
A F Q K R A Y S S **M S P I L G Y W K I K**

GGTTTGGTTCAACCTACAAGATTGTTATTGGAATATTTGGAAGAAAAGTACGAAGAACAT
G L V Q P T R L L L E Y L E E K Y E E H

TTGTACGAAAGAGATGAAGGAGATAAGTGGAGAAATAAGAAATTCGAATTGGGTTTGGAA
L Y E R D E G D K W R N K K F E L G L E

TTCCCAAATTTGCCTTACTACATCGATGGAGATGTTAAATTGACTCAATCAATGGCTATT
F P N L P Y Y I D G D V K L T Q S M A I

ATTAGATACATCGCTGATAAGCATAATATGTTGGGTGGTTGTCCTAAGGAAAGAGCTGAA
I R Y I A D K H N M L G G C P K E R A E

ATCTCTATGTTAGAAGGTGCTGTTTTGGATATTAGATACGGTGTTTCAAGAATTGCTTAT
I S M L E G A V L D I R Y G V S R I A Y

TCTAAGGATTTTGAAACATTGAAGGTTGATTTTCTTTCAAAGTTGCCAGAAATGTTGAAG
S K D F E T L K V D F L S K L P E M L K

ATGTTCGAAGATAGATTGTGTCATAAGACTTATTTGAATGGTGACCATGTTACACATCCT
M F E D R L C H K T Y L N G D H V T H P

GATTTTATGTTGTACGATGCTTTGGATGTTGTTTTGTACATGGACCCAATGTGTTTAGAT
D F M L Y D A L D V V L Y M D P M C L D

GCTTTTCCTAAGTTGGTTTGTTTCAAAAAGAGAATCGAAGCTATCCCACAAATCGATAAG
A F P K L V C F K K R I E A I P Q I D K

TACTTAAAATCTTCTAAATATATCGCTTGGCCTTTGCAAGGTTGGCAAGCTACTTTTGGT
Y L K S S K Y I A W P L Q G W Q A T F G

GGTGGAGATCATCCACCAAAATCTTCTTCTCCAACTAGACAATTACAAGTTTTGGATTCA
G G D H P P K S S S P T R Q L Q V L D S

GAAGATGATGGTACATTATTGAAAGTTGCTTTTGCTTCATCTGATAGAGAATTGGTTGAT
E D D G T L L K V A F A S S D R E L V D

CAACATTTTGGTTCATCTAGATCTTTTGCTATCTATGGTGTTAATCCTGAAAGATCACAA
Q H F G S S R S F A I Y G V N P E R S Q

TTGTTGTCTGTTGTTGAATTCGGTGAATTAGAACAAGATGGTAACGAAGATAAATTGGCT
L L S V V E F G E L E Q D G N E D K L A

AGAAAAATTGATTTGTTGGATGGTTGTGTTGCTGTTTATTGTTGTGCTTGTGGTGCTTCA
R K I D L L D G C V A V Y C C A C G A S

GCTGTTAGACAATTGATGGCTATTGGTGTTCAACCAATTAAGGTTTCTGAAGGTGCTAGA
A V R Q L M A I G V Q P I K V S E G A R

ATTGCTGAATTGATCGAAGCTTTGCAAGTTGAATTGAGAGAAGGTCCTTCTGCTTGGTTG
I A E L I E A L Q V E L R E G P S A W L

GCTAAAGCTATTCAAAGAACAAGAGGTCCAGATATGAGAAGATTTGATGCTATGGCTGCT
A K A I Q R T R G P D M R R F D A M A A

GAAGGTTGGGATGAGTAA
E G W D E -

SU9-**FdxN-HA**

ATGGCCTCCACTCGTGTTCTCGCCTCTCGCCTCGCCTCCCAGATGGCTGCCTCCGCCAAG
M A S T R V L A S R L A S Q M A A S A K

GTTGCTCGCCCTGCTGTCCGCGTTGCTCAAGTCAGCAAGCGCACCATCCAGACTGGCTCC
V A R P A V R V A Q V S K R T I Q T G S

CCTCTCCAGACCCTCAAGCGCACCCAGATGACCTCGATCGTCAACGCCACCACCCGCCAG
P L Q T L K R T Q M T S I V N A T T R Q

GCTTTTCAGAAGCGCGCCTACTCTTCCATGGCTCTTAAGATAGTTGAGTCTTGTGTGAAC
A F Q K R A Y S S **M A L K I V E S C V N**

TGCTGGGCATGTGTTGATGTGTGCCCAAGTGAGGCTATATCCTTGGCAGGTCCTCATTTT
C W A C V D V C P S E A I S L A G P H F

GAAATTTCTGCTTCAAAATGCACCGAGTGTGATGGAGACTATGCTGAAAAGCAATGCGCA
E I S A S K C T E C D G D Y A E K Q C A

TCTATTTGTCCAGTTGAAGGTGCTATCTTGTTAGCAGACGGAACTCCTGCTAACCCACCT
S I C P V E G A I L L A D G T P A N P P

GGTTCACTTACAGGAATCCCACCTGAAAGATTGGCTGAGGCAATGAGAGAAATACAGGCA
G S L T G I P P E R L A E A M R E I Q A

AGGTATCCATATGATGTTCCAGATTATGCTTAA
R Y P Y D V P D Y A -

SU9-**TS** (for NifB proteins)
ATGGCCTCCACTCGTGTCCTCGCCTCTCGCCTGGCCTCCCAGATGGCTGCTTCCGCCAAG
M A S T R V L A S R L A S Q M A A S A K

GTTGCCCGCCCTGCTGTCCGCGTTGCTCAGGTCAGCAAGCGCACCATCCAGACTGGCTCC
V A R P A V R V A Q V S K R T I Q T G S

CCCCTCCAGACCCTCAAGCGCACCCAGATGACCTCCATCGTCAACGCCACCACCCGCCAG
P L Q T L K R T Q M T S I V N A T T R Q

GCTTTCCAGAAGCGCGCCTACTCTTCCTCAGCATGGAGTCATCCTCAGTTTGAGAAAGGT
A F Q K R A Y S S S A **W S H P Q F E K G**

GGAGGTTCAGGTGGTGGAAGCGGTGGATCTGCTTGGTCACATCCACAATTTGAAAAA
G G S G G G S G G S A W S H P Q F E K

Yeast optimized DNA sequences, and their translated amino acid sequences, cloned in pESC expression vectors. N-terminus for NifB as deduced from N-terminal sequencing is highlighted in green (SSSAW).

SU9-**NifU,** (pN2GLT4)
ATGGCCTCCACTCGTGTCCTCGCCTCTCGCCTGGCCTCCCAGATGGCTGCTTCCGCCAAG
M A S T R V L A S R L A S Q M A A S A K

GTTGCCCGCCCTGCTGTCCGCGTTGCTCAGGTCAGCAAGCGCACCATCCAGACTGGCTCC
V A R P A V R V A Q V S K R T I Q T G S

CCCCTCCAGACCCTCAAGCGCACCCAGATGACCTCCATCGTCAACGCCACCACCCGCCAG
P L Q T L K R T Q M T S I V N A T T R Q

GCTTTCCAGAAGCGCGCCTACTCTTCCAGGCCTTGGGACTACTCTGAAAAGGTTAAGGAA
A F Q K R A Y S S R P **W D Y S E K V K E**

CATTTCTACAATCCAAAGAACGCCGGTGCTGTAGAAGGTGCAAACGCCATTGGTGACGTT
H F Y N P K N A G A V E G A N A I G D V

GGTTCATTATCCTGTGGTGACGCTTTGAGATTAACATTGAAAGTTGACCCTGAAACCGAT
G S L S C G D A L R L T L K V D P E T D

GTCATCTTGGACGCAGGTTTTCAAACTTTCGGTTGCGGTTCTGCTATTGCATCTTCATCC
V I L D A G F Q T F G C G S A I A S S S

GCTTTGACTGAAATGGTTAAGGGTTTGACATTGGATGAAGCATTGAAAATCTCAAACCAA
A L T E M V K G L T L D E A L K I S N Q

GATATCGCTGACTATTTGGATGGTTTGCCACCTGAAAAGATGCATTGTTCCGTCATGGGT
D I A D Y L D G L P P E K M H C S V M G

AGAGAAGCCTTACAAGCTGCAGTAGCTAACTACAGAGGTGAAACCATTGAAGATGACCAC
R E A L Q A A V A N Y R G E T I E D D H

GAAGAAGGTGCATTGATATGTAAATGCTTTGCCGTTGATGAAGTTATGGTCAGAGATACC
E E G A L I C K C F A V D E V M V R D T

ATAAGAGCAAATAAGTTAAGTACTGTAGAAGATGTTACTAACTACACAAAAGCTGGTGGT
I R A N K L S T V E D V T N Y T K A G G

GGTTGTTCTGCTTGCCATGAAGCAATAGAAAGAGTTTTGACAGAAGAATTGGCCGCTAGA
G C S A C H E A I E R V L T E E L A A R

GGTGAAGTATTCGTTGCAGCCCCAATTAAAGCCAAAAAGAAAGTCAAGGTATTGGCTCCA
G E V F V A A P I K A K K K V K V L A P

GAACCTGCCCCAGCTCCTGTTGCAGAAGCCCCAGCTGCAGCCCCTAAGTTGTCAAATTTG
E P A P A P V A E A P A A A P K L S N L

CAAAGAATTAGAAGAATCGAAACAGTCTTGGCTGCAATAAGACCTACCTTGCAAAGAGAC
Q R I R R I E T V L A A I R P T L Q R D

AAAGGTGACGTCGAATTAATTGATGTAGACGGTAAAAATGTTTACGTCAAATTGACCGGT
K G D V E L I D V D G K N V Y V K L T G

GCTTGTACTGGTTGCCAAATGGCATCCATGACATTAGGTGGTATACAACAAAGATTGATC
A C T G C Q M A S M T L G G I Q Q R L I

GAAGAATTGGGTGAGTTCGTCAAAGTTATCCCAGTCTCCGCTGCCGCACACGCCCAAATG
E E L G E F V K V I P V S A A A H A Q M

GAAGTCTGA
E V -

SU9-**NifS,** (pN2GLT4)
ATGGCCTCCACTCGTGTCCTCGCCTCTCGCCTGGCCTCCCAGATGGCTGCTTCCGCCAAG
M A S T R V L A S R L A S Q M A A S A K

GTTGCCCGCCCTGCTGTCCGCGTTGCTCAGGTCAGCAAGCGCACCATCCAGACTGGCTCC
V A R P A V R V A Q V S K R T I Q T G S

CCCCTCCAGACCCTCAAGCGCACCCAGATGACCTCCATCGTCAACGCCACCACCCGCCAG
P L Q T L K R T Q M T S I V N A T T R Q

GCTTTCCAGAAGCGCGCCTACTCTTCCGCAGCCATGGCCGACGTTTACTTGGATAATAAC
A F Q K R A Y S S A A **M A D V Y L D N N**

GCTACTACAAGAGTCGATGACGAAATAGTACAAGCTATGTTGCCATTTTTCACAGAACAA
A T T R V D D E I V Q A M L P F F T E Q

TTCGGTAACCCTTCCAGTTTGCATTCCTTCGGTAACCAAGTTGGTATGGCCTTGAAGAAA
F G N P S S L H S F G N Q V G M A L K K

GCTAGACAATCTGTCCAAAAATTGTTAGGTGCAGAACACGATTCCGAAATCGTTTTTACC
A R Q S V Q K L L G A E H D S E I V F T

AGTTGTGGTACTGAATCTGACTCAACCGCCATTTTGTCTGCCTTAAAAGCTCAACCAGAA
S C G T E S D S T A I L S A L K A Q P E

AGAAAGACTGTCATAACCACTGTTGTCGAACATCCTGCAGTATTGTCTTTATGCGATTAT
R K T V I T T V V E H P A V L S L C D Y

TTGGCCTCAGAAGGTTACACTGTTCATAAGTTACCAGTCGATAAAAAGGGTAGATTGGAC
L A S E G Y T V H K L P V D K K G R L D

TTAGAACACTATGCTTCCTTGTTAACAGATGACGTAGCTGTAGTTAGTGTTATGTGGGCA
L E H Y A S L L T D D V A V V S V M W A

AATAACGAAACTGGTACATTGTTTCCAATTGAAGAAATGGCAAGATTAGCCGATGACGCT
N N E T G T L F P I E E M A R L A D D A

GGTATAATGTTCCATACTGATGCAGTACAAGCCGTTGGTAAAGTCCCTATAGACTTGAAG
G I M F H T D A V Q A V G K V P I D L K

AACTCGTCAATCCACATGTTGTCCTTAAGTGGTCATAAATTGCACGCTCCAAAGGGTGTT
N S S I H M L S L S G H K L H A P K G V

GGTGTCTTGTACTTAAGAAGAGGTACAAGATTCAGACCTTTGTTAAGAGGTGGTCATCAA
G V L Y L R R G T R F R P L L R G G H Q

GAAAGAGGTAGAAGAGCCGGTACTGAAAATGCTGCATCTATTATAGGTTTGGGTGTTGCC
E R G R R A G T E N A A S I I G L G V A

GCTGAAAGAGCTTTACAATTCATGGAACATGAAAACACTGAAGTTAAGAGATTGCGTGAT
A E R A L Q F M E H E N T E V K R L R D

AAGTTAGAAGCAGGTATTTTGGCCGTCGTACCACACGCATTTGTTACTGGTGACCCAGAC
K L E A G I L A V V P H A F V T G D P D

AATAGATTACCTAACACAGCTAACATCGCATTCGAATACATCGAAGGTGAAGCTATCTTG
N R L P N T A N I A F E Y I E G E A I L

TTGTTGTTGAACAAAGTTGGTATAGCAGCCTCCAGTGGTTCTGCTTGTACATCTGGTTCA
L L L N K V G I A A S S G S A C T S G S

TTGGAACCATCACATGTTATGAGAGCAATGGATATTCCTTATACAGCTGCACACGGTACT
L E P S H V M R A M D I P Y T A A H G T

GTTAGATTTTCTTTGAGTAGATACACAACCGAAGAAGAAATTGATAGAGTCATTAGAGAA
V R F S L S R Y T T E E E I D R V I R E

GTACCACCTATTGTTGCTCAATTGAGAAAATTGTCTCCTTACTGGTCAGGTAATGGTCCT
V P P I V A Q L R K L S P Y W S G N G P

GTTGAAGACCCTGGTAAAGCCTTTGCTCCTGTCTATGGTTGA
V E D P G K A F A P V Y G -

SU9-**FdxN**, (pN2SB44, pN2SB45, pN2SB46)
ATGGCCTCCACTCGTGTCCTCGCCTCTCGCCTGGCCTCCCAGATGGCTGCTTCCGCCAAG
M A S T R V L A S R L A S Q M A A S A K

GTTGCCCGCCCTGCTGTCCGCGTTGCTCAGGTCAGCAAGCGCACCATCCAGACTGGCTCC
V A R P A V R V A Q V S K R T I Q T G S

CCCCTCCAGACCCTCAAGCGCACCCAGATGACCTCCATCGTCAACGCCACCACCCGCCAG
P L Q T L K R T Q M T S I V N A T T R Q

GCTTTCCAGAAGCGCGCCTACTCTTCCATGGCTCTTAAGATAGTTGAGTCTTGTGTGAAC
A F Q K R A Y S S **M A L K I V E S C V N**

TGCTGGGCATGTGTTGATGTGTGCCCAAGTGAGGCTATATCCTTGGCAGGTCCTCATTTT
C W A C V D V C P S E A I S L A G P H F

GAAATTTCTGCTTCAAAATGCACCGAGTGTGATGGAGACTATGCTGAAAAGCAATGCGCA
E I S A S K C T E C D G D Y A E K Q C A

TCTATTTGTCCAGTTGAAGGTGCTATCTTGTTAGCAGACGGAACTCCTGCTAACCCACCT
S I C P V E G A I L L A D G T P A N P P

GGTTCACTTACAGGAATCCCACCTGAAAGATTGGCTGAGGCAATGAGAGAAATACAGGCA
G S L T G I P P E R L A E A M R E I Q A

AGGTAA
R -

SU9-**FdxN-HA**, (pN2XJ155, pN2SB99-104)
ATGGCCTCCACTCGTGTCCTCGCCTCTCGCCTGGCCTCCCAGATGGCTGCTTCCGCCAAG
M A S T R V L A S R L A S Q M A A S A K

GTTGCCCGCCCTGCTGTCCGCGTTGCTCAGGTCAGCAAGCGCACCATCCAGACTGGCTCC
V A R P A V R V A Q V S K R T I Q T G S

CCCCTCCAGACCCTCAAGCGCACCCAGATGACCTCCATCGTCAACGCCACCACCCGCCAG
P L Q T L K R T Q M T S I V N A T T R Q

GCTTTCCAGAAGCGCGCCTACTCTTCCATGGCTCTTAAGATAGTTGAGTCTTGTGTGAAC
A F Q K R A Y S S **M A L K I V E S C V N**

TGCTGGGCATGTGTTGATGTGTGCCCAAGTGAGGCTATATCCTTGGCAGGTCCTCATTTT
C W A C V D V C P S E A I S L A G P H F

GAAATTTCTGCTTCAAAATGCACCGAGTGTGATGGAGACTATGCTGAAAAGCAATGCGCA
E I S A S K C T E C D G D Y A E K Q C A

TCTATTTGTCCAGTTGAAGGTGCTATCTTGTTAGCAGACGGAACTCCTGCTAACCCACCT
S I C P V E G A I L L A D G T P A N P P

GGTTCACTTACAGGAATCCCACCTGAAAGATTGGCTGAGGCAATGAGAGAAATACAGGCA
G S L T G I P P E R L A E A M R E I Q A

AGGTATCCATATGATGTTCCAGATTATGCTTAA
R Y P Y D V P D Y A -

SU9-**TS-MiNifB**, (pN2SB44, pN2XJ154, pN2XJ155)
ATGGCCTCCACTCGTGTCCTCGCCTCTCGCCTGGCCTCCCAGATGGCTGCTTCCGCCAAG
M A S T R V L A S R L A S Q M A A S A K

GTTGCCCGCCCTGCTGTCCGCGTTGCTCAGGTCAGCAAGCGCACCATCCAGACTGGCTCC
V A R P A V R V A Q V S K R T I Q T G S

CCCCTCCAGACCCTCAAGCGCACCCAGATGACCTCCATCGTCAACGCCACCACCCGCCAG
P L Q T L K R T Q M T S I V N A T T R Q

GCTTTCCAGAAGCGCGCCTACTCTTCCTCAGCATGGAGTCATCCTCAGTTTGAGAAAGGT
A F Q K R A Y S S S A **W S H P Q F E K G**

GGAGGTTCAGGTGGTGGAAGCGGTGGATCTGCTTGGTCACATCCACAATTTGAAAAACTC
G G S G G G S G G S A W S H P Q F E K L

GAAGGATCCATGGAAAAGATGTCCAAGTTCTCCCATTTGTTGAAAGCTCATCCATGCTTC
E G S **M E K M S K F S H L L K A H P C F**

AACGAAAAGGTTCATGATAAGTACGGTAGAGTTCATTTGCCAGTTGCTCCAAGATGTAAC
N E K V H D K Y G R V H L P V A P R C N

ATTGCTTGTAAGTTCTGCAAGAGGTCCGTTTCTAAAGAATGTTGTGAACATAGACCAGGT
I A C K F C K R S V S K E C C E H R P G

GTTTCTTTGGGTGTTTTGAAACCAGAAGATGTTGAGGACTACCTGAAAAAGATCTTGAAA
V S L G V L K P E D V E D Y L K K I L K

GAGATGCCAAACATCAAGGTTGTTGGTATTGCTGGTCCTGGTGATTCTCTGTTTAACAAA
E M P N I K V V G I A G P G D S L F N K

GAAACTTTCGAAACCCTGAAGATCATCGACGAAAAGTTTCCCAACTTGATTAAGTGCATT
E T F E T L K I I D E K F P N L I K C I

TCCACCAACGGTCTGTTGTTGTCTAAGTACTACAAGGATTTGGCCAACTTGAACGTTAGA
S T N G L L L S K Y Y K D L A N L N V R

ACTATTACCGTTACTGTCAACGCCATTAAGCCAGAAATCTTGGAAAAAATCGTTGACTGG
T I T V T V N A I K P E I L E K I V D W

GTTTACTACGACAAGAAGTTGTATAGAGGTTTGGAAGGTGCCAAGTTGTTGATCGAAAAA
V Y Y D K K L Y R G L E G A K L L I E K

CAAATCGAAGGTATCAAGAAGGCCTCCGAAGAAGATTTCATTATCAAGATCAACACCGTC
Q I E G I K K A S E E D F I I K I N T V

TTGATCCCAGAAATCAACATGGATCACGTTGTTGAAATTGCCAAGTTCTTCAAGGATTAC
L I P E I N M D H V V E I A K F F K D Y

GCCTACGTTCAAAACATCATTCCATTGATTCCACAGTACAAGATGAAGGAATTGAGAGCA
A Y V Q N I I P L I P Q Y K M K E L R A

CCAACTTGCGAAGAAATCAAAAAGGTCAGAAAAGAGTGCGAGAAGTACATCCCACAATTC
P T C E E I K K V R K E C E K Y I P Q F

AGAGCTTGTGGTCAATGTAGAGCTGATGCTGTTGGTCTGATCAAAGAAAAAGAGCTGTTG
R A C G Q C R A D A V G L I K E K E L L

AAAGAGTTTTTCAAAGAGAAGAACAAAGAAAAGAACATCAAGCTGGAAGTGTTCGACTTG
K E F F K E K N K E K N I K L E V F D L

AAGCACTTCTCTCATTGA
K H F S H -

SU9-**S**-**MiNifB,** (pN2SB45)

ATGGCCTCCACTCGTGTCCTCGCCTCTCGCCTGGCCTCCCAGATGGCTGCTTCCGCCAAG
M A S T R V L A S R L A S Q M A A S A K

GTTGCCCGCCCTGCTGTCCGCGTTGCTCAGGTCAGCAAGCGCACCATCCAGACTGGCTCC
V A R P A V R V A Q V S K R T I Q T G S

CCCCTCCAGACCCTCAAGCGCACCCAGATGACCTCCATCGTCAACGCCACCACCCGCCAG
P L Q T L K R T Q M T S I V N A T T R Q

GCTTTCCAGAAGCGCGCCTACTCTTCCTCAGCATGGAGTCATCCTCAGTTTGAGAAACTC
A F Q K R A Y S S S A **W S H P Q F E K** L

GAAGGATCCATGGAAAAGATGTCCAAGTTCTCCCATTTGTTGAAAGCTCATCCATGCTTC
E G S **M E K M S K F S H L L K A H P C F**

AACGAAAAGGTTCATGATAAGTACGGTAGAGTTCATTTGCCAGTTGCTCCAAGATGTAAC
N E K V H D K Y G R V H L P V A P R C N

ATTGCTTGTAAGTTCTGCAAGAGGTCCGTTTCTAAAGAATGTTGTGAACATAGACCAGGT
I A C K F C K R S V S K E C C E H R P G

GTTTCTTTGGGTGTTTTGAAACCAGAAGATGTTGAGGACTACCTGAAAAAGATCTTGAAA
V S L G V L K P E D V E D Y L K K I L K

GAGATGCCAAACATCAAGGTTGTTGGTATTGCTGGTCCTGGTGATTCTCTGTTTAACAAA
E M P N I K V V G I A G P G D S L F N K

GAAACTTTCGAAACCCTGAAGATCATCGACGAAAAGTTTCCCAACTTGATTAAGTGCATT
E T F E T L K I I D E K F P N L I K C I

TCCACCAACGGTCTGTTGTTGTCTAAGTACTACAAGGATTTGGCCAACTTGAACGTTAGA
S T N G L L L S K Y Y K D L A N L N V R

ACTATTACCGTTACTGTCAACGCCATTAAGCCAGAAATCTTGGAAAAAATCGTTGACTGG
T I T V T V N A I K P E I L E K I V D W

GTTTACTACGACAAGAAGTTGTATAGAGGTTTGGAAGGTGCCAAGTTGTTGATCGAAAAA
V Y Y D K K L Y R G L E G A K L L I E K

CAAATCGAAGGTATCAAGAAGGCCTCCGAAGAAGATTTCATTATCAAGATCAACACCGTC
Q I E G I K K A S E E D F I I K I N T V

TTGATCCCAGAAATCAACATGGATCACGTTGTTGAAATTGCCAAGTTCTTCAAGGATTAC
L I P E I N M D H V V E I A K F F K D Y

GCCTACGTTCAAAACATCATTCCATTGATTCCACAGTACAAGATGAAGGAATTGAGAGCA
A Y V Q N I I P L I P Q Y K M K E L R A

CCAACTTGCGAAGAAATCAAAAAGGTCAGAAAAGAGTGCGAGAAGTACATCCCACAATTC
P T C E E I K K V R K E C E K Y I P Q F

AGAGCTTGTGGTCAATGTAGAGCTGATGCTGTTGGTCTGATCAAAGAAAAAGAGCTGTTG
R A C G Q C R A D A V G L I K E K E L L

AAAGAGTTTTTCAAAGAGAAGAACAAAGAAAAGAACATCAAGCTGGAAGTGTTCGACTTG
K E F F K E K N K E K N I K L E V F D L

AAGCACTTCTCTCATTGA
K H F S H -

SU9-**10xHis**-**MiNifB,** (pN2SB46)
ATGGCCTCCACTCGTGTCCTCGCCTCTCGCCTGGCCTCCCAGATGGCTGCTTCCGCCAAG
M A S T R V L A S R L A S Q M A A S A K

GTTGCCCGCCCTGCTGTCCGCGTTGCTCAGGTCAGCAAGCGCACCATCCAGACTGGCTCC
V A R P A V R V A Q V S K R T I Q T G S

CCCCTCCAGACCCTCAAGCGCACCCAGATGACCTCCATCGTCAACGCCACCACCCGCCAG
P L Q T L K R T Q M T S I V N A T T R Q

GCTTTCCAGAAGCGCGCCTACTCTTCCCATCATCACCATCACCATCACCATCACCATCTC
A F Q K R A Y S S **H H H H H H H H H H** L

GAAGGATCCATGGAAAAGATGTCCAAGTTCTCCCATTTGTTGAAAGCTCATCCATGCTTC
E G S **M E K M S K F S H L L K A H P C F**

AACGAAAAGGTTCATGATAAGTACGGTAGAGTTCATTTGCCAGTTGCTCCAAGATGTAAC
N E K V H D K Y G R V H L P V A P R C N

ATTGCTTGTAAGTTCTGCAAGAGGTCCGTTTCTAAAGAATGTTGTGAACATAGACCAGGT
I A C K F C K R S V S K E C C E H R P G

GTTTCTTTGGGTGTTTTGAAACCAGAAGATGTTGAGGACTACCTGAAAAAGATCTTGAAA
V S L G V L K P E D V E D Y L K K I L K

GAGATGCCAAACATCAAGGTTGTTGGTATTGCTGGTCCTGGTGATTCTCTGTTTAACAAA
E M P N I K V V G I A G P G D S L F N K

GAAACTTTCGAAACCCTGAAGATCATCGACGAAAAGTTTCCCAACTTGATTAAGTGCATT
E T F E T L K I I D E K F P N L I K C I

TCCACCAACGGTCTGTTGTTGTCTAAGTACTACAAGGATTTGGCCAACTTGAACGTTAGA
S T N G L L L S K Y Y K D L A N L N V R

ACTATTACCGTTACTGTCAACGCCATTAAGCCAGAAATCTTGGAAAAAATCGTTGACTGG
T I T V T V N A I K P E I L E K I V D W

GTTTACTACGACAAGAAGTTGTATAGAGGTTTGGAAGGTGCCAAGTTGTTGATCGAAAAA
V Y Y D K K L Y R G L E G A K L L I E K

CAAATCGAAGGTATCAAGAAGGCCTCCGAAGAAGATTTCATTATCAAGATCAACACCGTC
Q I E G I K K A S E E D F I I K I N T V

TTGATCCCAGAAATCAACATGGATCACGTTGTTGAAATTGCCAAGTTCTTCAAGGATTAC
L I P E I N M D H V V E I A K F F K D Y

GCCTACGTTCAAAACATCATTCCATTGATTCCACAGTACAAGATGAAGGAATTGAGAGCA
A Y V Q N I I P L I P Q Y K M K E L R A

CCAACTTGCGAAGAAATCAAAAAGGTCAGAAAAGAGTGCGAGAAGTACATCCCACAATTC
P T C E E I K K V R K E C E K Y I P Q F

AGAGCTTGTGGTCAATGTAGAGCTGATGCTGTTGGTCTGATCAAAGAAAAAGAGCTGTTG
R A C G Q C R A D A V G L I K E K E L L

AAAGAGTTTTTCAAAGAGAAGAACAAAGAAAAGAACATCAAGCTGGAAGTGTTCGACTTG
K E F F K E K N K E K N I K L E V F D L

AAGCACTTCTCTCATTGA
K H F S H -

SU9-**TS-AvNifB,** (pN2SB99)
ATGGCCTCCACTCGTGTCCTCGCCTCTCGCCTGGCCTCCCAGATGGCTGCTTCCGCCAAG
M A S T R V L A S R L A S Q M A A S A K

GTTGCCCGCCCTGCTGTCCGCGTTGCTCAGGTCAGCAAGCGCACCATCCAGACTGGCTCC
V A R P A V R V A Q V S K R T I Q T G S

CCCCTCCAGACCCTCAAGCGCACCCAGATGACCTCCATCGTCAACGCCACCACCCGCCAG
P L Q T L K R T Q M T S I V N A T T R Q

GCTTTCCAGAAGCGCGCCTACTCTTCCTCAGCATGGAGTCATCCTCAGTTTGAGAAAGGT
A F Q K R A Y S S S A **W S H P Q F E K G**

GGAGGTTCAGGTGGTGGAAGCGGTGGATCTGCTTGGTCACATCCACAATTTGAAAAAATG
G G S G G G S G G S A W S H P Q F E K M

ACTCCACCACCAACTGGTTTGTTGACTTCTGCTACTCCACAAACAAAAACCCAACCTAAG
T P P P T G L L T S A T P Q T K T Q P K

TCTAACTCTTGCGGTTGTTCTTCTAAAACTGATACCACTACTGGTCTGGACGAAAAGATT
S N S C G C S S K T D T T T G L D E K I

AAGGCCAGAATTGAAAAACACCCATGCTACTCTGAAGAGGCTCATCATCATTATGCTAGA
K A R I E K H P C Y S E E A H H H Y A R

ATGCATGTTGCTGTTGCTCCAGCTTGTAACATTCAATGTAACTACTGCAACAGAAAGTAC
M H V A V A P A C N I Q C N Y C N R K Y

GATTGCGCTAATGAATCTAGACCAGGTGTTGTCTCTGAATTATTGACTCCAGAAGAAGCT
D C A N E S R P G V V S E L L T P E E A

GCTCACAAGGTTTTGGTTATTGCTGGTAAAATTCCACAGATGACCGTTTTAGGTATTGCA
A H K V L V I A G K I P Q M T V L G I A

GGTCCAGGTGATCCATTGGCTAATCCAGAAAAAACTTTCAGGACCTTCGAATTGATTGCT
G P G D P L A N P E K T F R T F E L I A

GAAAAAGCCCCAGATATCAAGTTGTGTTTGTCTACCAATGGTTTGATGTTGCCAGATTAC
E K A P D I K L C L S T N G L M L P D Y

GTTGACAGAATCAAGCAATTGAACATCGATCATGTTACCATCACCATCAACATGGTTGAT
V D R I K Q L N I D H V T I T I N M V D

CCAGAAATTGGCACTAAGATCTATCCATGGGTTCACTACAGAAGAAAGAGATACAAAGGT
P E I G T K I Y P W V H Y R R K R Y K G

ATTGAAGCTGCCCAAATCTTGCACGAAAAACAAATGGAAGGTCTACAAGCTTTACAAGAG
I E A A Q I L H E K Q M E G L Q A L Q E

GCTGATATTTTGTGCAAGGTTAACTCCGTTATGATCCCAGGTATTAACGATGAACACTTG
A D I L C K V N S V M I P G I N D E H L

GTTGAAGTTAACCAGGTCATCAGATCTAAAGGTGCTTTCTTGCATAACATCATGCCATTG
V E V N Q V I R S K G A F L H N I M P L

ATTTCTGCTCCAGAACATGGTACTCATTTTGGTTTGACTGGTCAAAGAGGTCCAACTGCT
I S A P E H G T H F G L T G Q R G P T A

AAAGAATTGAAGCAAATCCAAGATAACTGTGCCGGCAATATGAAGATGATGAGACATTGC
K E L K Q I Q D N C A G N M K M M R H C

AGACAATGCAGAGCTGATGCTGTTGGTTTGTTAGGTGAAGATAGATCTCAAGAGTTCACC
R Q C R A D A V G L L G E D R S Q E F T

AAAGAAAAGTTCATGGAAATGGCTCCAGAGTACAACTTGGAACAAAGACAAACTGTTCAT
K E K F M E M A P E Y N L E Q R Q T V H

GCCGGTATCGAAAAGTCCCAAAAAGAAATTCAAGTCGTCAAAGAGAAGGTCGTCGAAACA
A G I E K S Q K E I Q V V K E K V V E T

TTGCAAACTACCTCATTCAACAACTCCCCAAAGATTTTGGTTGCCGTTGCTACAAAAGGT
L Q T T S F N N S P K I L V A V A T K G

GGTGGTTTGGTTAATCAACATTTCGGTCATGCCAAAGAATTCATGATCTACGAAGTTGAT
G G L V N Q H F G H A K E F M I Y E V D

GGTAAGAGCGCTAAGTTCGTTTCCCATAGAAAGATTGATCACTACTGCCAATCTGGTTAT
G K S A K F V S H R K I D H Y C Q S G Y

GGTGAAGAAGCTACCTTGGATAACATTATCCATGCCATTTCTGATTGCCAAGCCGTTTTG
G E E A T L D N I I H A I S D C Q A V L

GTTTCTAAGATTGGTAATTGTCCACAAGAGCAATTATTGAAGGCTGGCTTGCAAACTGTT
V S K I G N C P Q E Q L L K A G L Q T V

GAAGCCTACGATGTTATTGAAAAGGTTGCCTTGGAATTCTACGAGAAGTGGATTTTGGAA
E A Y D V I E K V A L E F Y E K W I L E

GCCAGAGATTGA
A R D -

SU9-**TS-RaNifB,** (PN2SB100)
ATGGCCTCCACTCGTGTCCTCGCCTCTCGCCTGGCCTCCCAGATGGCTGCTTCCGCCAAG
M A S T R V L A S R L A S Q M A A S A K

GTTGCCCGCCCTGCTGTCCGCGTTGCTCAGGTCAGCAAGCGCACCATCCAGACTGGCTCC
V A R P A V R V A Q V S K R T I Q T G S

CCCCTCCAGACCCTCAAGCGCACCCAGATGACCTCCATCGTCAACGCCACCACCCGCCAG
P L Q T L K R T Q M T S I V N A T T R Q

GCTTTCCAGAAGCGCGCCTACTCTTCCTCAGCATGGAGTCATCCTCAGTTTGAGAAAGGT
A F Q K R A Y S S S A **W S H P Q F E K G**

GGAGGTTCAGGTGGTGGAAGCGGTGGATCTGCTTGGTCACATCCACAATTTGAAAAAATG
G G S G G G S G G S A W S H P Q F E K M

GCCATCTCTTACGAAGAATTGACTACCAAACATCCATGTTTCGCTAGAGGTGAAAAAAAC
A I S Y E E L T T K H P C F A R G E K N

GGTTCTGGTAGAATTCACTTGCCAATTTCTCCATCCTGTAACATCGAATGTAGATTCTGC
G S G R I H L P I S P S C N I E C R F C

GAAAGGTCCTTCAACAACTACGAAATTAGACCAGGTGTTTCTAGAACCGTTATTACTCCT
E R S F N N Y E I R P G V S R T V I T P

GAAGAAGCTTTGGACGCTATTAGAAGGGCTTTAGAAGTTTGCCCAGATATTCATGTTGCT
E E A L D A I R R A L E V C P D I H V A

GGTATTGCTGGTCCAGGTGATACTTTGGCTTCTCCATATGCTTTGGAAACCTTCAAGTTG
G I A G P G D T L A S P Y A L E T F K L

ATCAAAGAAGAATACCCAGAACTGGTCAAGTGTATGTCTACTAATGGTTTGTTGTTGGCT
I K E E Y P E L V K C M S T N G L L L A

GAAAAGGCCCAAGAAATTTTGGATGTTGGTATTGATTCTTTGACCGTTACCGTTAATGCT
E K A Q E I L D V G I D S L T V T V N A

GTTGATCCAGAAATTGAAGCCAAGTTGAACGATGGTATTATCTGGCATGGTAAACACTAC
V D P E I E A K L N D G I I W H G K H Y

ACTGGTGTTGAAGCTGCCAAGATTTTGATCGAACAACAATTGAAGGGCATCAAGATATTG
T G V E A A K I L I E Q Q L K G I K I L

TCTGATGCTGGTATGACTTTGAAGGTCAACACTGTTTTTGTCCCAGAAATCAACGGTGAT
S D A G M T L K V N T V F V P E I N G D

CACATTGAAGAAGTTGCTAGAACTGTTGCTGAAGCTGGTGCTACTATCTACAACATTATT
H I E E V A R T V A E A G A T I Y N I I

CCATTGATCCCCAACCACAAGTTGAAGGATTGCAGAGAACCAGATTGTGTCGAATTGGAA
P L I P N H K L K D C R E P D C V E L E

AGAGTTATTTTGAAGGCCTCCAAGTACATCGACGTTTTCAGACATTGTCAAAGATGTAGA
R V I L K A S K Y I D V F R H C Q R C R

GCTGATGCAGTTGGTATTCCAGGTGGTAAAGATTATGGTGAAGAGATCTACCAAAACTTG
A D A V G I P G G K D Y G E E I Y Q N L

CAGAGATTGACTAGAAAGGATACCTTCTCTCATGGCTGA
Q R L T R K D T F S H G -

SU9-**TS-MiNifB,** (PN2SB101)
ATGGCCTCCACTCGTGTCCTCGCCTCTCGCCTGGCCTCCCAGATGGCTGCTTCCGCCAAG
M A S T R V L A S R L A S Q M A A S A K

GTTGCCCGCCCTGCTGTCCGCGTTGCTCAGGTCAGCAAGCGCACCATCCAGACTGGCTCC

V A R P A V R V A Q V S K R T I Q T G S

CCCCTCCAGACCCTCAAGCGCACCCAGATGACCTCCATCGTCAACGCCACCACCCGCCAG

P L Q T L K R T Q M T S I V N A T T R Q

GCTTTCCAGAAGCGCGCCTACTCTTCCTCAGCATGGAGTCATCCTCAGTTTGAGAAAGGT

A F Q K R A Y S S S A **W S H P Q F E K G**

GGAGGTTCAGGTGGTGGAAGCGGTGGATCTGCTTGGTCACATCCACAATTTGAAAAAATG

G G S G G G S G G S A W S H P Q F E K M

GAAAAGATGTCCAAGTTCTCCCATTTGTTGAAAGCTCATCCATGCTTCAACGAAAAGGTT

E K M S K F S H L L K A H P C F N E K V

CATGATAAGTACGGTAGAGTTCATTTGCCAGTTGCTCCAAGATGTAACATTGCTTGTAAG

H D K Y G R V H L P V A P R C N I A C K

TTCTGCAAGAGGTCCGTTTCTAAAGAATGTTGTGAACATAGACCAGGTGTTTCTTTGGGT

F C K R S V S K E C C E H R P G V S L G

GTTTTGAAACCAGAAGATGTTGAGGACTACCTGAAAAAGATCTTGAAAGAGATGCCAAAC

V L K P E D V E D Y L K K I L K E M P N

ATCAAGGTTGTTGGTATTGCTGGTCCTGGTGATTCTCTGTTTAACAAAGAAACTTTCGAA

I K V V G I A G P G D S L F N K E T F E

ACCCTGAAGATCATCGACGAAAAGTTTCCCAACTTGATTAAGTGCATTTCCACCAACGGT

T L K I I D E K F P N L I K C I S T N G

CTGTTGTTGTCTAAGTACTACAAGGATTTGGCCAACTTGAACGTTAGAACTATTACCGTT

L L L S K Y Y K D L A N L N V R T I T V

ACTGTCAACGCCATTAAGCCAGAAATCTTGGAAAAAATCGTTGACTGGGTTTACTACGAC

T V N A I K P E I L E K I V D W V Y Y D

AAGAAGTTGTATAGAGGTTTGGAAGGTGCCAAGTTGTTGATCGAAAAACAAATCGAAGGT

K K L Y R G L E G A K L L I E K Q I E G

ATCAAGAAGGCCTCCGAAGAAGATTTCATTATCAAGATCAACACCGTCTTGATCCCAGAA

I K K A S E E D F I I K I N T V L I P E

ATCAACATGGATCACGTTGTTGAAATTGCCAAGTTCTTCAAGGATTACGCCTACGTTCAA

I N M D H V V E I A K F F K D Y A Y V Q

AACATCATTCCATTGATTCCACAGTACAAGATGAAGGAATTGAGAGCACCAACTTGCGAA

N I I P L I P Q Y K M K E L R A P T C E

GAAATCAAAAAGGTCAGAAAAGAGTGCGAGAAGTACATCCCACAATTCAGAGCTTGTGGT
E I K K V R K E C E K Y I P Q F R A C G

CAATGTAGAGCTGATGCTGTTGGTCTGATCAAAGAAAAAGAGCTGTTGAAAGAGTTTTTC
Q C R A D A V G L I K E K E L L K E F F

AAAGAGAAGAACAAAGAAAAGAACATCAAGCTGGAAGTGTTCGACTTGAAGCACTTCTCT
K E K N K E K N I K L E V F D L K H F S

CATTGA
H -

SU9-**TS-MaNifB,** (pN2SB102)
ATGGCCTCCACTCGTGTCCTCGCCTCTCGCCTGGCCTCCCAGATGGCTGCTTCCGCCAAG
M A S T R V L A S R L A S Q M A A S A K

GTTGCCCGCCCTGCTGTCCGCGTTGCTCAGGTCAGCAAGCGCACCATCCAGACTGGCTCC
V A R P A V R V A Q V S K R T I Q T G S

CCCCTCCAGACCCTCAAGCGCACCCAGATGACCTCCATCGTCAACGCCACCACCCGCCAG
P L Q T L K R T Q M T S I V N A T T R Q

GCTTTCCAGAAGCGCGCCTACTCTTCCTCAGCATGGAGTCATCCTCAGTTTGAGAAAGGT
A F Q K R A Y S S S A **W S H P Q F E K G**

GGAGGTTCAGGTGGTGGAAGCGGTGGATCTGCTTGGTCACATCCACAATTTGAAAAAATG
G G S G G G S G G S A W S H P Q F E K M

CCAGAAGAAAACCAGCCAATCAAAGAAAAGAACAACGGTCCAATTTTGGGCGAAGAGTTG
P E E N Q P I K E K N N G P I L G E E L

TTGAGAAAGATTTCTGAACATCCATGCTACGATAAGAACGCCCAACATAAGTATGGTAGA
L R K I S E H P C Y D K N A Q H K Y G R

ATTCATTTGGCTGTTGCTCCAGCTTGTAACATCCAATGTAATTTCTGCGTTAGAGAATTC
I H L A V A P A C N I Q C N F C V R E F

GACTGCGTCAATGAATCTAGACCAGGTGTTACTTCTAAGGTTTTGACTCCAGAAGAAGCT
D C V N E S R P G V T S K V L T P E E A

TTGGAAAAGACCAAGCAAATTTTGGCTGAATACCCATTCATTAAGGTTGTTGCTATTGCT
L E K T K Q I L A E Y P F I K V V A I A

GGTCCAGGTGATCCATTGGCTAATGACGAAACTTTTGAAACCTTCGAGTTGATCAGAAAC
G P G D P L A N D E T F E T F E L I R N

GAATTCCCAGAAATCACCTTGTGTATGTCTACCAATGGTTTGATGTTGCCAGAAAAGTTG
E F P E I T L C M S T N G L M L P E K L

CCTGAAATTTTGAGAACTGGTGTTTCTACTTTGACCGTTACCGTTAATGCTATCGATCCA
P E I L R T G V S T L T V T V N A I D P

GAAATTCAAGCCAAGATCGTTGATCATATCTTCTACCACGGTAAGGTTTACAAAGGTGTT
E I Q A K I V D H I F Y H G K V Y K G V

GAAGCTGCTAAGATCCAAATCAAGAATCAATTGGATGGTATTAAGGCTGCTATTGATGCT
E A A K I Q I K N Q L D G I K A A I D A

GGTATCGTTGTTAAGGTTAACACCGTTTTGATTCCAGGCATTAACGATAAGCACATTATC
G I V V K V N T V L I P G I N D K H I I

GAGATTGCCAAGAAATTGAACGAATTGGGTGTCTACATCATGAACGTTATGCCATTGATT
E I A K K L N E L G V Y I M N V M P L I

AACCAGGGTGCTTTTGCTGATTTGGAACCACCAACACCTGAAGAAAGAAAAGCTGTTCAA
N Q G A F A D L E P P T P E E R K A V Q

GAGGCTTGTGAACCATACGTTATGCAAATGAGACATTGCAGACAATGTAGAGCTGATGCT
E A C E P Y V M Q M R H C R Q C R A D A

TATGGTTTGTTGGCTCAAGATATGTCCCAAATGTCTGAAGAGAGAAGAAAGGTCATTAAG
Y G L L A Q D M S Q M S E E R R K V I K

ATCCAGACCAAAGAAGATATGGAAAAGGCTAGAGCCGTCTTGGAAAAAAACGGTAAGAAA
I Q T K E D M E K A R A V L E K N G K K

GAAGCTTAA
E A -

SU9-**TS-MtNifB,** (PN2SB103)
ATGGCCTCCACTCGTGTCCTCGCCTCTCGCCTGGCCTCCCAGATGGCTGCTTCCGCCAAG
M A S T R V L A S R L A S Q M A A S A K

GTTGCCCGCCCTGCTGTCCGCGTTGCTCAGGTCAGCAAGCGCACCATCCAGACTGGCTCC
V A R P A V R V A Q V S K R T I Q T G S

CCCCTCCAGACCCTCAAGCGCACCCAGATGACCTCCATCGTCAACGCCACCACCCGCCAG
P L Q T L K R T Q M T S I V N A T T R Q

GCTTTCCAGAAGCGCGCCTACTCTTCCTCAGCATGGAGTCATCCTCAGTTTGAGAAAGGT
A F Q K R A Y S S S A **W S H P Q F E K G**

GGAGGTTCAGGTGGTGGAAGCGGTGGATCTGCTTGGTCACATCCACAATTTGAAAAAATG
G G S G G G S G G S A W S H P Q F E K M

CCAGATCAAAGACAAACCAGATTCGCTCATATTACTAAGGCTCATCCATGCTTCAACGAA
P D Q R Q T R F A H I T K A H P C F N E

AAGTTGCATGATAGAGTTGGTAGAGTTCATGTTCCAATTGCTCCAAGATGTAACATCCAT
K L H D R V G R V H V P I A P R C N I H

TGCAAGTTCTGTACCAGAGATATCAACGAATGTGAAAGACGTCCAGGTGTTACTGGTAGA
C K F C T R D I N E C E R R P G V T G R

TTGATGACTGCTGATGATGCTATTAAGCACGTCGAAAAGGTCAAAGAAGAAATGCCAATT
L M T A D D A I K H V E K V K E E M P I

TCCGTTATTGGTGTTGCTGGTCCAGGTGATGCTTTGGCTAATGAAGAAACTTTCGAGTTC
S V I G V A G P G D A L A N E E T F E F

TTCAAGAAGGCCTCTAAGAAGTTTCCAGATTTGTTGAAGTGTATGTCCACCAACGGTTTG
F K K A S K K F P D L L K C M S T N G L

TTGTTGCCAGATAGAGCTGATGAATTGGCTGAATTGGGTATTAACACTGTTACTGTTACC
L L P D R A D E L A E L G I N T V T V T

GTTAACGCTGTTGATCCAGAAATTGGTGAAAAGATCTACTCCTTCGTTGTCTACAAGGAT
V N A V D P E I G E K I Y S F V V Y K D

AAGGTTTATCATGGTAGAGAAGCCTTCGAAGTGTTGTCTAGAAATCAATTGGAAGGCATT
K V Y H G R E A F E V L S R N Q L E G I

GAAAAGTTGGCCGAAAGAGGTATTATCGTCAAGGTTAACTCTGTTTTGATCCCAGGTTTG
E K L A E R G I I V K V N S V L I P G L

AACGATGAACATATTGTCGATATTGCCCGTGAAGTTAAGAAAAGGGGTGCTTCTTTGATG
N D E H I V D I A R E V K K R G A S L M

AACATCATTCCATTGATTCCAATGGGTGAGATGAAGGATTATCCAAGACCAACCTGTGAA
N I I P L I P M G E M K D Y P R P T C E

CAAATCGAAAGAGTTAGAAACGAAGTCGAGAAGATCATCCCAGTTTTTAGAGCTTGTACT
Q I E R V R N E V E K I I P V F R A C T

CAATGTAGAGCAGATGCTTATGGTATCCCAGGTAAAAAAGAAGCTGATAAGCACTTGGAT
Q C R A D A Y G I P G K K E A D K H L D

ATGACCCCAGCTTCTCATTACTAA
M T P A S H Y -

SU9-**TS-SsNifB,** (pN2SB104)

```
ATGGCCTCCACTCGTGTTCTCGCCTCTCGCCTGGCCTCCCAGATGGCTGCTTCCGCCAAG
 M  A  S  T  R  V  L  A  S  R  L  A  S  Q  M  A  A  S  A  K

GTTGCCCGCCCTGCTGTCCGCGTTGCTCAGGTCAGCAAGCGCACCATCCAGACTGGCTCC
 V  A  R  P  A  V  R  V  A  Q  V  S  K  R  T  I  Q  T  G  S

CCCCTCCAGACCCTCAAGCGCACCCAGATGACCTCCATCGTCAACGCCACCACCCGCCAG
 P  L  Q  T  L  K  R  T  Q  M  T  S  I  V  N  A  T  T  R  Q

GCTTTCCAGAAGCGCGCCTACTCTTCCTCAGCATGGAGTCATCCTCAGTTTGAGAAAGGT
 A  F  Q  K  R  A  Y  S  S  S  A  W  S  H  P  Q  F  E  K  G

GGAGGTTCAGGTGGTGGAAGCGGTGGATCTGCTTGGTCACATCCACAATTTGAAAAAATG
 G  G  S  G  G  G  S  G  G  S  A  W  S  H  P  Q  F  E  K  M

CCACCATTGTCTACTCCACCAAGACAAGGTTCTCCATTTCCATCTGCTTGTGCTTGTTCT
 P  P  L  S  T  P  P  R  Q  G  S  P  F  P  S  A  C  A  C  S

TCATCTCCTAGACAAGCTTTGCCACCACACTTGCAACAAAGAATTGCTACTCATCCATGT
 S  S  P  R  Q  A  L  P  P  H  L  Q  Q  R  I  A  T  H  P  C

TACTCTGAAGCTGCTCATCATCATTATGCCAGAATGCATGTTGCTGTTGCTCCAGCTTGT
 Y  S  E  A  A  H  H  H  Y  A  R  M  H  V  A  V  A  P  A  C

AACATTCAATGTAACTACTGCAACAGAAAGTTCGATTGCGCTAATGAATCTAGACCAGGT
 N  I  Q  C  N  Y  C  N  R  K  F  D  C  A  N  E  S  R  P  G

GTTGTCTCTGAATTATTGACTCCAGCTGAAGCAGCTCACAAGGTTTTGGTTATTGCTGGT
 V  V  S  E  L  L  T  P  A  E  A  A  H  K  V  L  V  I  A  G

AAAATTCCACAATTGACCGTTGTTGGTATTGCAGGTCCAGGTGATCCATTGGCTAATCCA
 K  I  P  Q  L  T  V  V  G  I  A  G  P  G  D  P  L  A  N  P

AAACAAACTTTTGAAACCTTCGCCAGAATTGCTGAAAAAGCTCCAGATTTGAAGCTGTGT
 K  Q  T  F  E  T  F  A  R  I  A  E  K  A  P  D  L  K  L  C

TTGTCTACCAATGGTTTGATGTTGCCAGATTACATCGACGAAATCAAGAGATTGAACATC
 L  S  T  N  G  L  M  L  P  D  Y  I  D  E  I  K  R  L  N  I

GATCATGTTACCCTGACCATCAATATGGTTGATCCAGAAATTGGTGCTAGAATCTACCCA
 D  H  V  T  L  T  I  N  M  V  D  P  E  I  G  A  R  I  Y  P

TGGATTAGATGGCGTAGAAAAAGAATCAGAGGTGTTGAAGCTGCCAGAATATTGCACGAA
 W  I  R  W  R  R  K  R  I  R  G  V  E  A  A  R  I  L  H  E
```

AGACAAATGGAATCCTTGGACTTGTTGAGAGAAGCTGATATTTTGTGCAAGGTCAACTCC
R Q M E S L D L L R E A D I L C K V N S

GTTTTGATTCCAGGTATTAACGATACCCACTTGTTGGAAGTTAACAGGGTTATTCAAGAA
V L I P G I N D T H L L E V N R V I Q E

AAGGGTGCCTTCTTGCATAACATCATGCCATTGATTTCTGCTCCAGAACATGGTACTTAC
K G A F L H N I M P L I S A P E H G T Y

TTCGGTTTGATTGGTCAAAGAGGTCCAACTCCACAAGAATTGAAAGCCTTGCAAGATAGA
F G L I G Q R G P T P Q E L K A L Q D R

TGTTCCGGTAATATGAGAATGATGAGACATTGCAGACAATGCAGAGCTGATGCTGTTGGT
C S G N M R M M R H C R Q C R A D A V G

TTGTTGGGTGAAGATAGATCTCAAGAGTTTACCAAAGAAAGGTTCATGCAAATGACCCCA
L L G E D R S Q E F T K E R F M Q M T P

GAATACGATCCTGAAAAAAGAAGGCAAGTTCAAGCTTCCATTGCTCAATTGCAAGCTCAA
E Y D P E K R R Q V Q A S I A Q L Q A Q

CAACAATCTACTCCAACTCATTGGACTGCTGATTTGCCACCTGATTCTCCATCTATTTTG
Q Q S T P T H W T A D L P P D S P S I L

GTTGCAGTTGCTTCTAAAGGTGGTGGTTTGGTTAATAGACATTTCGGTCATGCTAGGGAA
V A V A S K G G G L V N R H F G H A R E

TTCTTGATCTACGAAGTTAATGCTAGAGGTGCTCAATTCGTTGGTCATAGAAAAGTTCCT
F L I Y E V N A R G A Q F V G H R K V P

CAATACTGTCATGGTGGTTCTGGTGAGGATAACAACTTGGAACAAATCTTGGATTTGATC
Q Y C H G G S G E D N N L E Q I L D L I

AGAGATTGCAGAGCCGTTTTGGTTTCTAAGATTGGTGATTGTCCAATGAAGAGGATTAGA
R D C R A V L V S K I G D C P M K R I R

GAAGCAGGTATCGAAGTTGTTGAAGATTACGATTTGATTGAGACTGTCGCCTTGAAGTTT
E A G I E V V E D Y D L I E T V A L K F

TACAGAACCTGGTTGGTTAATCAAGCCTTGGATTCCAAATCCTCCAGATCTTAA
Y R T W L V N Q A L D S K S S R S -

SU9-**TS**-*TEV*-**NifX** (pN2SB97, pN2SB106)
ATGGCCTCCACTCGTGTCCTCGCCTCTCGCCTGGCCTCCCAGATGGCTGCTTCCGCCAAG
M A S T R V L A S R L A S Q M A A S A K

GTTGCCCGCCCTGCTGTCCGCGTTGCTCAGGTCAGCAAGCGCACCATCCAGACTGGCTCC
V A R P A V R V A Q V S K R T I Q T G S

CCCCTCCAGACCCTCAAGCGCACCCAGATGACCTCCATCGTCAACGCCACCACCCGCCAG
P L Q T L K R T Q M T S I V N A T T R Q

GCTTTCCAGAAGCGCGCCTACTCTTCCTCAGCATGGAGTCATCCTCAGTTTGAGAAAGGT
A F Q K R A Y S S S A **W S H P Q F E K G**

GGAGGTTCAGGTGGTGGAAGCGGTGGATCTGCTTGGTCACATCCACAATTTGAAAAACTC
G G S G G G S G G S A W S H P Q F E K L

GAAGGAGGCGGCGAGAACCTGTACTTCCAGGGCGGCGGCTCCTCCCCGACTAGGCAGCTC
E G G G *E N L Y F Q G* G G **S S P T R Q L**

CAGGTGCTGGACTCTGAGGACGATGGCACACTCCTGAAGGTGGCCTTCGCCTCCTCCGAC
Q V L D S E D D G T L L K V A F A S S D

AGGGAGCTCGTCGATCAGCACTTCGGCTCGTCCCGGAGCTTCGCCATCTACGGAGTCAAC
R E L V D Q H F G S S R S F A I Y G V N

CCTGAGCGCAGCCAGCTCCTGTCTGTGGTCGAGTTCGGCGAGCTGGAGCAGGACGGCAAT
P E R S Q L L S V V E F G E L E Q D G N

GAGGATAAGCTCGCCCGCAAGATCGACCTCCTGGATGGCTGCGTGGCCGTCTACTGCTGC
E D K L A R K I D L L D G C V A V Y C C

GCCTGCGGAGCATCAGCGGTGAGGCAGCTGATGGCGATCGGCGTGCAGCCGATCAAGGTG
A C G A S A V R Q L M A I G V Q P I K V

TCCGAGGGAGCAAGGATCGCGGAGCTCATTGAGGCCCTGCAGGTCGAGCTGAGGGAGGGA
S E G A R I A E L I E A L Q V E L R E G

CCTTCTGCCTGGCTCGCCAAGGCGATCCAGCGGACCCGCGGCCCAGACATGCGCAGGTTC
P S A W L A K A I Q R T R G P D M R R F

GATGCCATGGCGGCCGAGGGATGGGATGAGTGA
D A M A A E G W D E -

SU9-**Sam1p**-**FLAG** (pN2SB97)
ATGGCCTCCACTCGTGTCCTCGCCTCACGCCTGGCCTCCCAGATGGCTGCTTCCGCCAAA
M A S T R V L A S R L A S Q M A A S A K

GTTGCCCGCCCTGCTGTCCGCGTTGCCCAGGTCAGCAAGCGCACCATCCAGACTGGGTCC
V A R P A V R V A Q V S K R T I Q T G S

CCCCTCCAGACCCTCAAGCGCACCCAAATGACCTCCATCGTCAACGCCACCACTCGCCAG
P L Q T L K R T Q M T S I V N A T T R Q

GCTTTCCAGAAGCGCGCCTACTCTTCGATGGCCGGTACATTTTTATTCACTTCTGAATCC
A F Q K R A Y S S **M A G T F L F T S E S**

GTTGGTGAAGGTCACCCAGATAAGATCTGTGACCAAGTTTCCGACGCCATCTTGGACGCT
V G E G H P D K I C D Q V S D A I L D A

TGTTTAGCCGAGGACCCTCACTCCAAAGTTGCGTGTGAAACCGCGGCAAAGACTGGTATG
C L A E D P H S K V A C E T A A K T G M

ATTATGGTCTTTGGTGAAATTACTACCAAGGCACAGTTGGATTACCAAAAAATCGTCAGA
I M V F G E I T T K A Q L D Y Q K I V R

GACACCATCAAGAAGATTGGTTACGATGATTCCGCCAAGGGTTTCGACTATAAGACCTGT
D T I K K I G Y D D S A K G F D Y K T C

AACGTCCTTGTCGCCATTGAGCAACAATCTCCAGATATCGCCCAAGGTGTCCACGAGGAG
N V L V A I E Q Q S P D I A Q G V H E E

AAGGATTTGGAAGACATCGGTGCCGGTGACCAAGGTATCATGTTTGGTTACGCCACAGAT
K D L E D I G A G D Q G I M F G Y A T D

GAAACTCCAGAGGGTTTGCCTTTGACTATTCTTTTGGCTCATAAACTAAACATGGCCATG
E T P E G L P L T I L L A H K L N M A M

GCTGACGCGAGAAGAGATGGCTCTTTAGCGTGGTTGAGACCAGACACCAAGACTCAAGTC
A D A R R D G S L A W L R P D T K T Q V

ACCGTCGAATACAAGGATGACCACGGTAGATGGGTTCCACAAAGAATCGACACCGTCGTC
T V E Y K D D H G R W V P Q R I D T V V

GTCTCCGCTCAACATGCTGACGAAATCACGACCGAGGACTTAAGAGCGCAACTAAAGTCC
V S A Q H A D E I T T E D L R A Q L K S

GAGATCATTGAAAAAGTCATCCCAAGAGACATGTTGGACGAAAACACCAAATACTTTATC
E I I E K V I P R D M L D E N T K Y F I

CAACCTTCCGGTAGATTCGTCATCGGTGGTCCTCAAGGTGACGCTGGTTTGACCGGTAGA
Q P S G R F V I G G P Q G D A G L T G R

AAGATCATCGTCGACGCTTACGGTGGTGCCTCATCCGTCGGTGGTGGTGCCTTCTCCGGT
K I I V D A Y G G A S S V G G G A F S G

AAGGACTACTCTAAGGTTGATCGTTCTGCCGCTTATGCCGCTAGATGGGTTGCCAAGTCC
K D Y S K V D R S A A Y A A R W V A K S

CTAGTTGCCGCTGGTTTATGTAAGAGAGTTCAAGTTCAATTTTCTTATGCCATCGGTATT
L V A A G L C K R V Q V Q F S Y A I G I

GCGGAACCATTGTCCTTGCACGTTGACACCTATGGTACTGCGACCAAGTCTGACGAAGAA
A E P L S L H V D T Y G T A T K S D E E

ATTATCGACATTATCAGCAAGAACTTTGACTTGAGACCTGGTGTATTGGTCAAGGAGTTG
I I D I I S K N F D L R P G V L V K E L

GACTTAGCTAGACCAATCTACTTGCCAACCGCTTCTTATGGCCATTTCACAAACCAAGAA
D L A R P I Y L P T A S Y G H F T N Q E

TACCCATGGGAAAAGCCTAAGACTTTGAAGTTCGCACTAGTATCGATGGATTACAAGGAT
Y P W E K P K T L K F A L V S M **D Y K D**

GACGACGATAAGATCTGA
D D D K I -

Sam1p-FLAG (pN2SB106)
ATGGCCGGTACATTTTTATTCACTTCTGAATCCGTTGGTGAAGGTCACCCAGATAAGATC
M A G T F L F T S E S V G E G H P D K I

TGTGACCAAGTTTCCGACGCCATCTTGGACGCTTGTTTAGCCGAGGACCCTCACTCCAAA
C D Q V S D A I L D A C L A E D P H S K

GTTGCGTGTGAAACCGCGGCAAAGACTGGTATGATTATGGTCTTTGGTGAAATTACTACC
V A C E T A A K T G M I M V F G E I T T

AAGGCACAGTTGGATTACCAAAAAATCGTCAGAGACACCATCAAGAAGATTGGTTACGAT
K A Q L D Y Q K I V R D T I K K I G Y D

GATTCCGCCAAGGGTTTCGACTATAAGACCTGTAACGTCCTTGTCGCCATTGAGCAACAA
D S A K G F D Y K T C N V L V A I E Q Q

TCTCCAGATATCGCCCAAGGTGTCCACGAGGAGAAGGATTTGGAAGACATCGGTGCCGGT
S P D I A Q G V H E E K D L E D I G A G

GACCAAGGTATCATGTTTGGTTACGCCACAGATGAAACTCCAGAGGGTTTGCCTTTGACT
D Q G I M F G Y A T D E T P E G L P L T

ATTCTTTTGGCTCATAAACTAAACATGGCCATGGCTGACGCGAGAAGAGATGGCTCTTTA
I L L A H K L N M A M A D A R R D G S L

GCGTGGTTGAGACCAGACACCAAGACTCAAGTCACCGTCGAATACAAGGATGACCACGGT
A W L R P D T K T Q V T V E Y K D D H G

AGATGGGTTCCACAAAGAATCGACACCGTCGTCGTCTCCGCTCAACATGCTGACGAAATC
R W V P Q R I D T V V V S A Q H A D E I

ACGACCGAGGACTTAAGAGCGCAACTAAAGTCCGAGATCATTGAAAAAGTCATCCCAAGA
T T E D L R A Q L K S E I I E K V I P R

GACATGTTGGACGAAAACACCAAATACTTTATCCAACCTTCCGGTAGATTCGTCATCGGT
D M L D E N T K Y F I Q P S G R F V I G

GGTCCTCAAGGTGACGCTGGTTTGACCGGTAGAAAGATCATCGTCGACGCTTACGGTGGT
G P Q G D A G L T G R K I I V D A Y G G

GCCTCATCCGTCGGTGGTGGTGCCTTCTCCGGTAAGGACTACTCTAAGGTTGATCGTTCT
A S S V G G G A F S G K D Y S K V D R S

GCCGCTTATGCCGCTAGATGGGTTGCCAAGTCCCTAGTTGCCGCTGGTTTATGTAAGAGA
A A Y A A R W V A K S L V A A G L C K R

GTTCAAGTTCAATTTTCTTATGCCATCGGTATTGCGGAACCATTGTCCTTGCACGTTGAC
V Q V Q F S Y A I G I A E P L S L H V D

ACCTATGGTACTGCGACCAAGTCTGACGAAGAAATTATCGACATTATCAGCAAGAACTTT
T Y G T A T K S D E E I I D I I S K N F

GACTTGAGACCTGGTGTATTGGTCAAGGAGTTGGACTTAGCTAGACCAATCTACTTGCCA
D L R P G V L V K E L D L A R P I Y L P

ACCGCTTCTTATGGCCATTTCACAAACCAAGAATACCCATGGAAAAGCCTAAGACTTTG
T A S Y G H F T N Q E Y P W E K P K T L

AAGTTCGCACTAGTATCGATGGATTACAAGGATGACGACGATAAGATCTGA
K F A L V S M **D Y K D D D D K** I -

Supplementary Figures

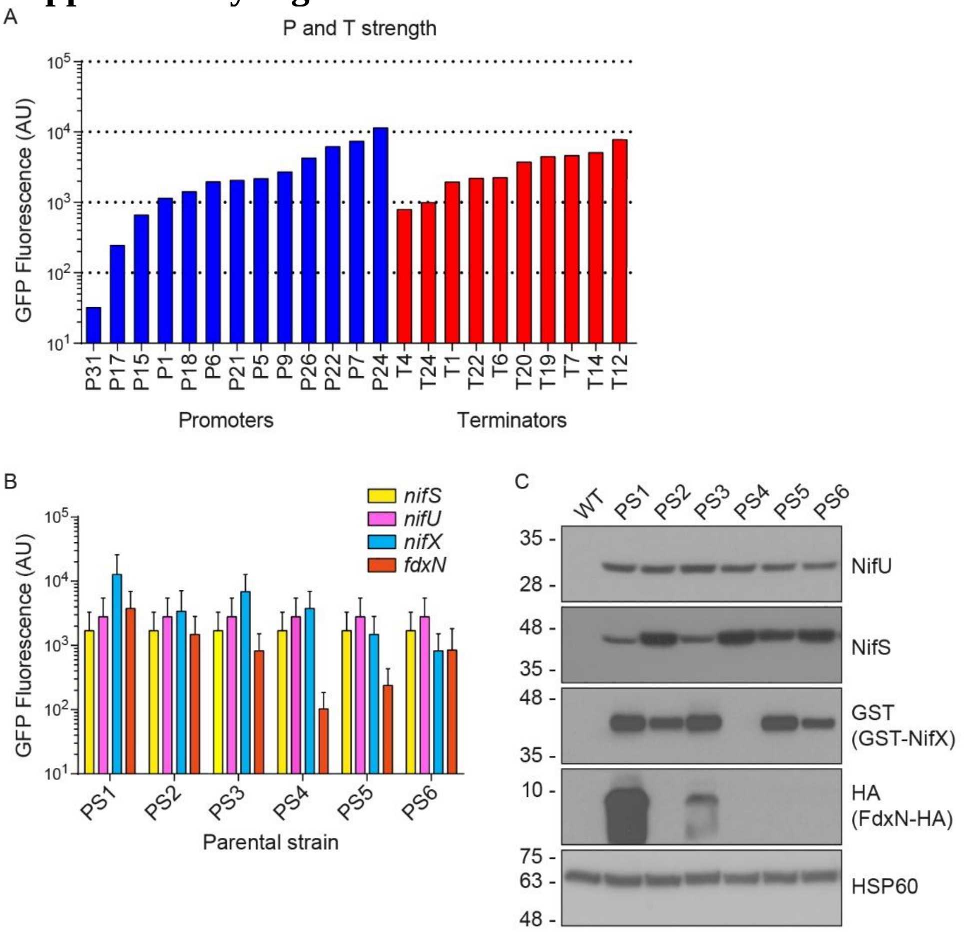

D

		NifS Clone 1	NifS Clone 2	NifS Clone 3	NifS Clone 4	NifU Clone 1	NifU Clone 2	NifU Clone 3	NifU Clone 4	FdxN Clone 1	FdxN Clone 2	FdxN Clone 3	FdxN Clone 4	NifX Clone 1	NifX Clone 2	NifX Clone 3	NifX Clone 4
PS	1	1	1	1	1	4	4	4	4	0	4	0	0	4	4	4	4
	2	4	3	4	4	4	4	4	4	0	0	0	0	3	4	4	3
	3	1	1	0	1	4	4	0	4	2	2	0	2	4	4	0	4
	4	4	3	0	0	4	4	0	1	0	0	0	0	2	0	0	4
	5	2	2	4	4	4	4	4	4	0	0	0	0	4	4	3	3
	6	4	4	4	4	4	4	4	4	0	0	0	0	4	4	3	3

Score	
0	Neg
1	Weak
2	
3	
4	Strong

Fig. S1. Expression level information used in the design of *S. cerevisiae* parental and NifB strains. (*A*) Part strengths of individual promoters and terminators used in this study, as previously measured using GFP in previous work (6). (*B*) Estimated *nif* gene expression according to levels for promoter-terminator cassettes observed using GFP in previous work (6). (*C*) Western blot analysis of total protein extracts to verify expression of NifU, NifS, NifX (GST-NifX) and FdxN (FdxN-HA) in the six parental strains (PS). Protein extract from wild-type *S. cerevisiae* (WT) was used as control for the specificity of the antibodies. Panel represent analysis of clone 2 of each PS. Antibodies targeting HSP60 was used as protein extract loading control. (*D*) Expression analysis NifS, NifU, FdxN-HA (FdxN) and GST-NifX (NifX) in four individual clones of PS1-PS6. Protein expression as seen from Western blotting was scored as 0 (not detected) or 1 to 4 (weak to strong). Clones 3 of PS3 and PS4 did not express detectable levels of any of the four proteins and were considered as false positives.

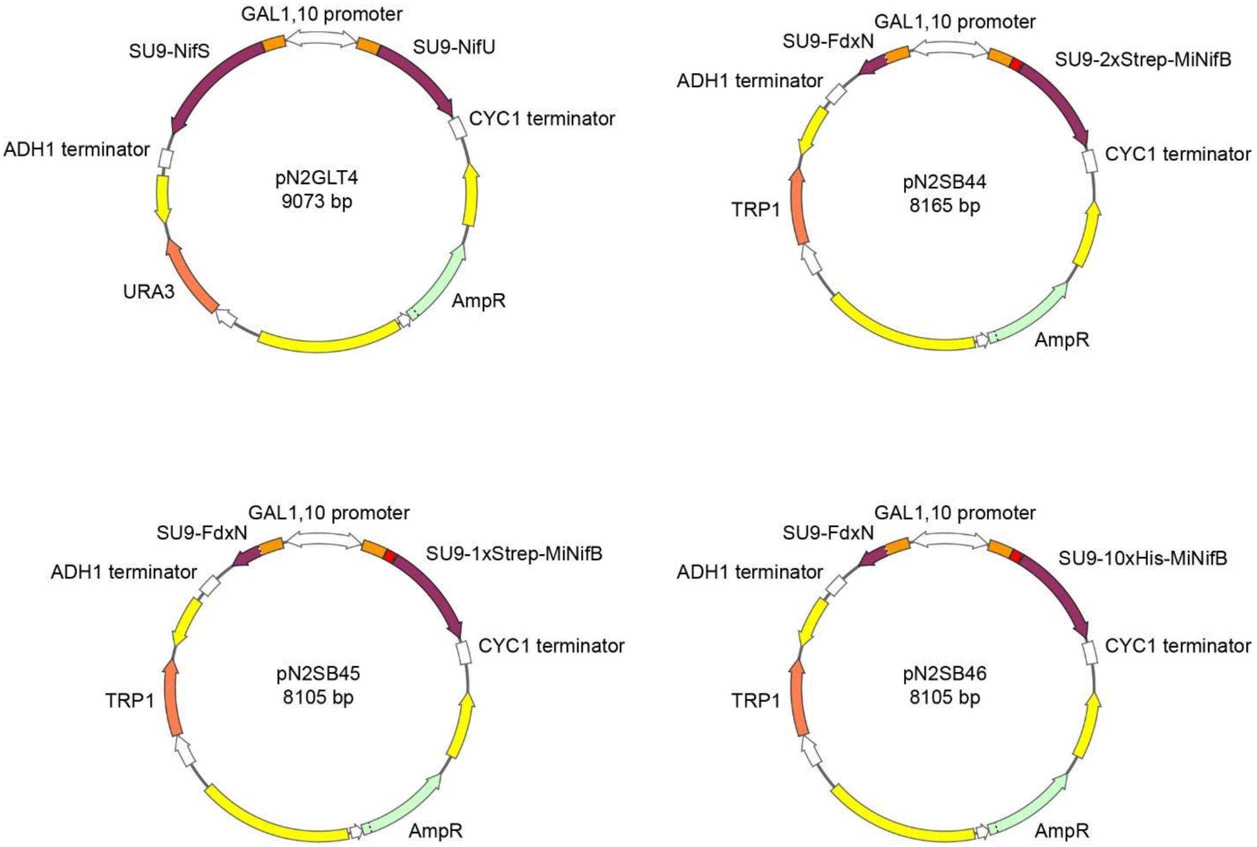

Fig. S2. Simplified DNA maps for yeast expression plasmids used in strains SB17Y, SB18Y, and SB19Y.

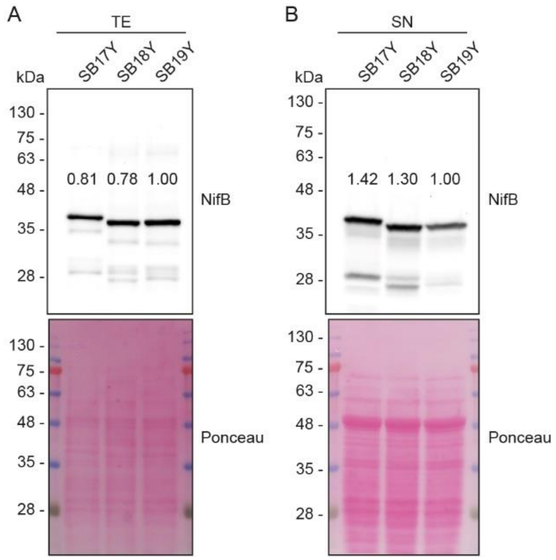

Fig. S3. Western blot analysis using antibodies targeting *M. infernus* NifB in total extracts (*A*) and soluble extracts (*B*) of *S. cerevisiae* strains expressing 2xStrep-tagged (SB17Y), 1xStrep-tagged (SB18Y), and 10xHis-tagged (SB19Y) *M. infernus* NifB protein. Numbers indicate signal intensity relative to that of the 10xHis-tagged protein.

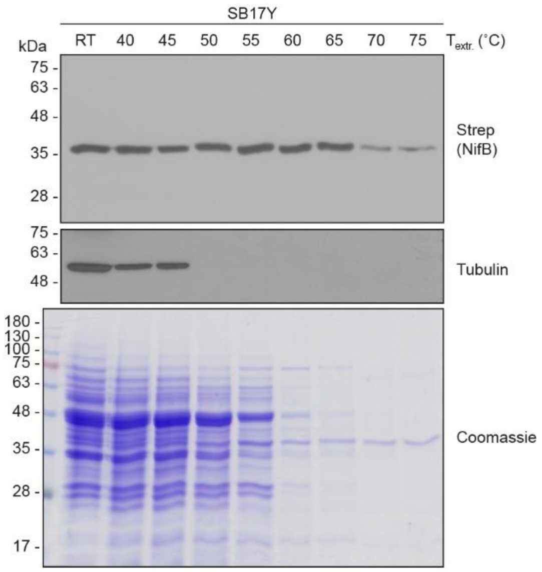

Fig. S4. Solubility at different temperatures of *M. infernus* NifB expressed in *S. cerevisiae* strain SB17Y. Following cell lysis, extracts were incubated at increasing temperatures. The soluble fractions upon centrifugation were analyzed by SDS-PAGE and Western blotting. Heat-induced precipitation of yeast proteins in the extract at the different temperatures is shown using antibodies recognizing tubulin, as well as by Coomassie staining of proteins from the extract resolved by SDS-PAGE.

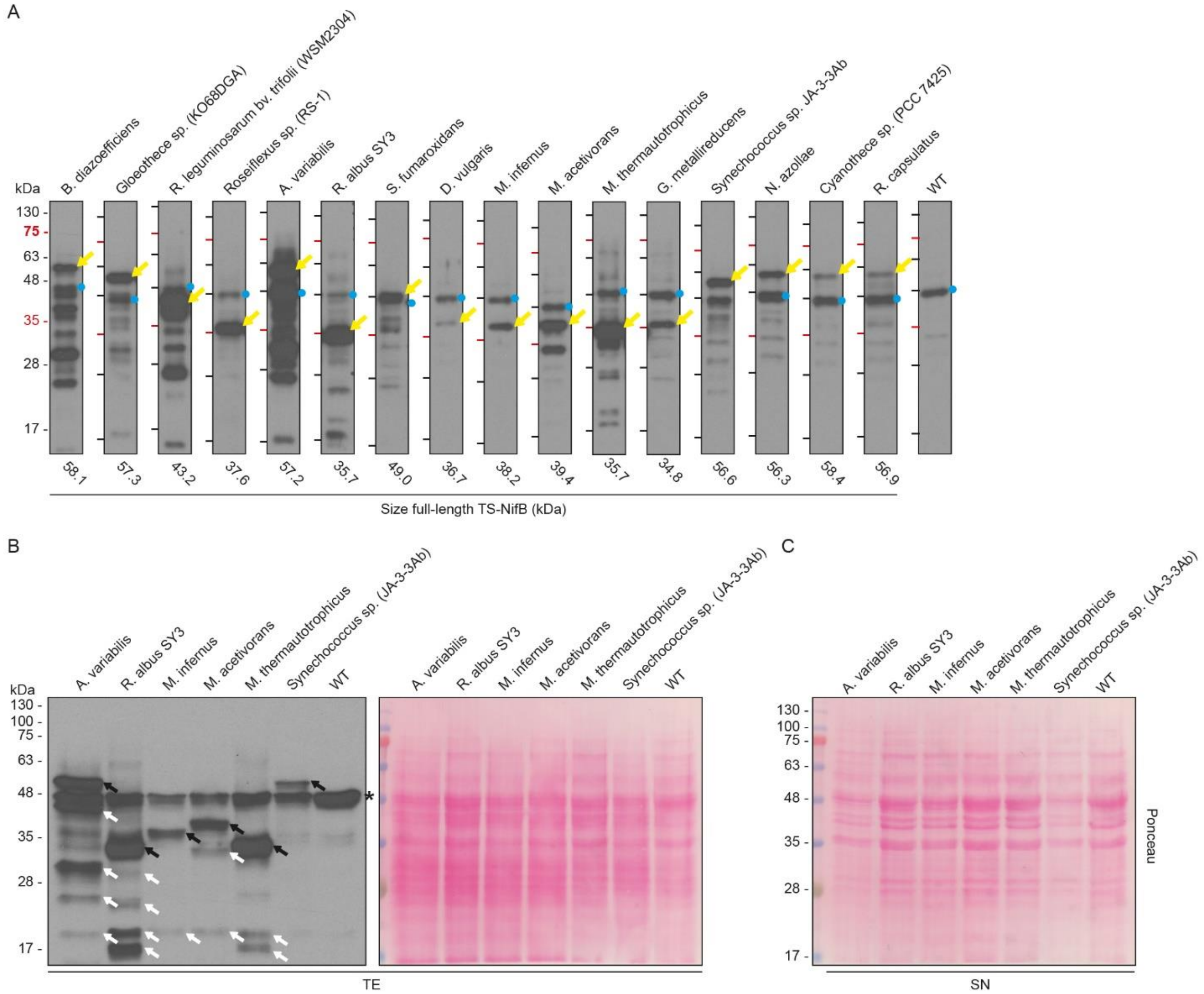

Fig. S5. Analysis of NifB expression in *S. cerevisiae* library. (*A*) Migration of the 16 expressed NifB proteins in total yeast extracts. Yellow arrow indicate migration of full-length NifB. Blue dot indicate non-specific signal. (*B*) Total extracts of strains expressing the six soluble NifB candidates shown in Fig. 2*E*. Black arrow indicate migration of full-length NifB protein. White arrows indicate NifB degradation products. Black star indicate non-specific signal. (*C*) Ponceau stained membrane of corresponding Western blot shown in Fig. 2*E*. Protein extract from wild-type *S. cerevisiae* (WT) was used as control for the specificity of the antibodies.

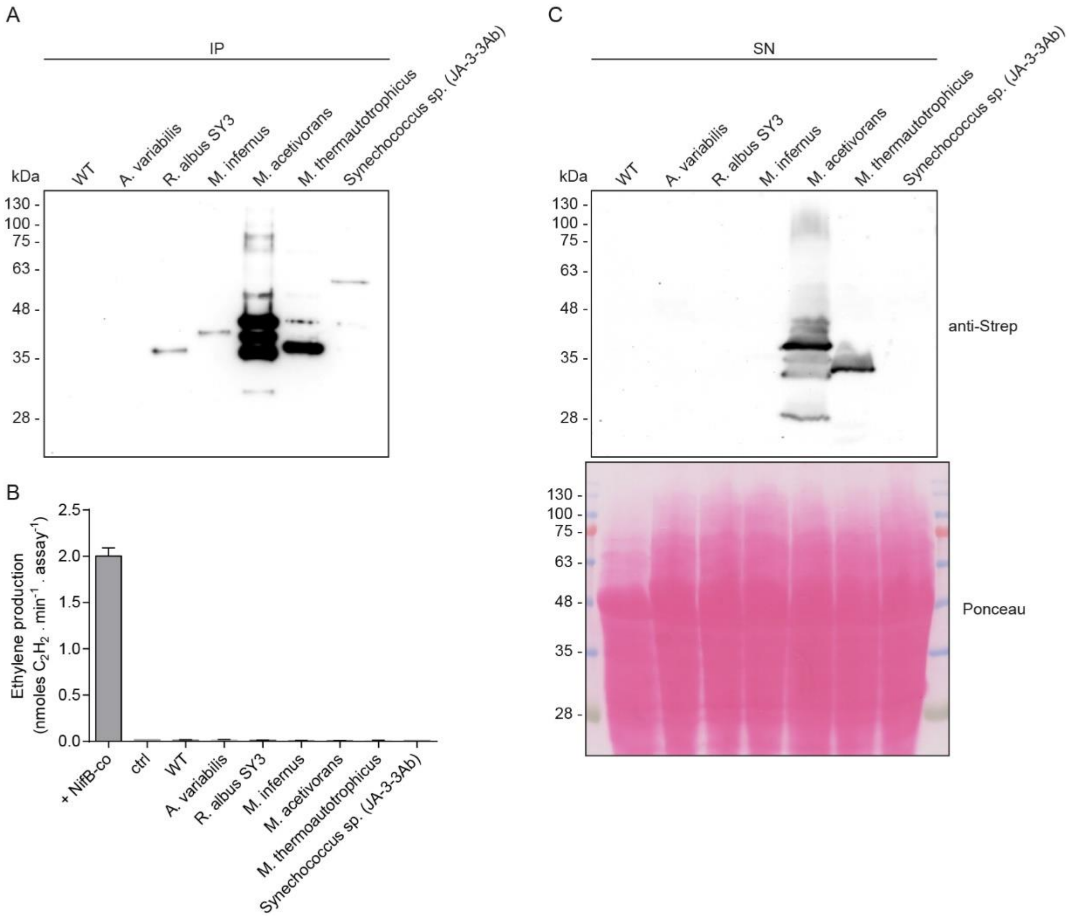

Fig. S6. Western blot analysis (*A*) and *in vitro* synthesis of FeMo-co and apo-NifDK reconstitution assay (*B*) using Δ*nifB A. vinelandii* (UW140) cell-free extracts of Strep-Tactin pulldowns of soluble extracts (*C*) of *S. cerevisiae* strains expressing NifB originating from *A. variabilis*, *R. albus* SY3, *M. infernus*, *M. acetivorans*, *M. thermautotrophicus*, and *Synechococcus sp.* JA-3-3Ab, or from wild-type *S. cerevisiae* (WT). Positive control reactions for the assay contained purified NifB-co (+ NifB-co). Negative control reactions for assay were performed in the absence of NifB-co (ctrl). Error bars represent mean ± standard deviation (n=2). The soluble extracts for the assay were prepared using the YeastBuster extraction method.

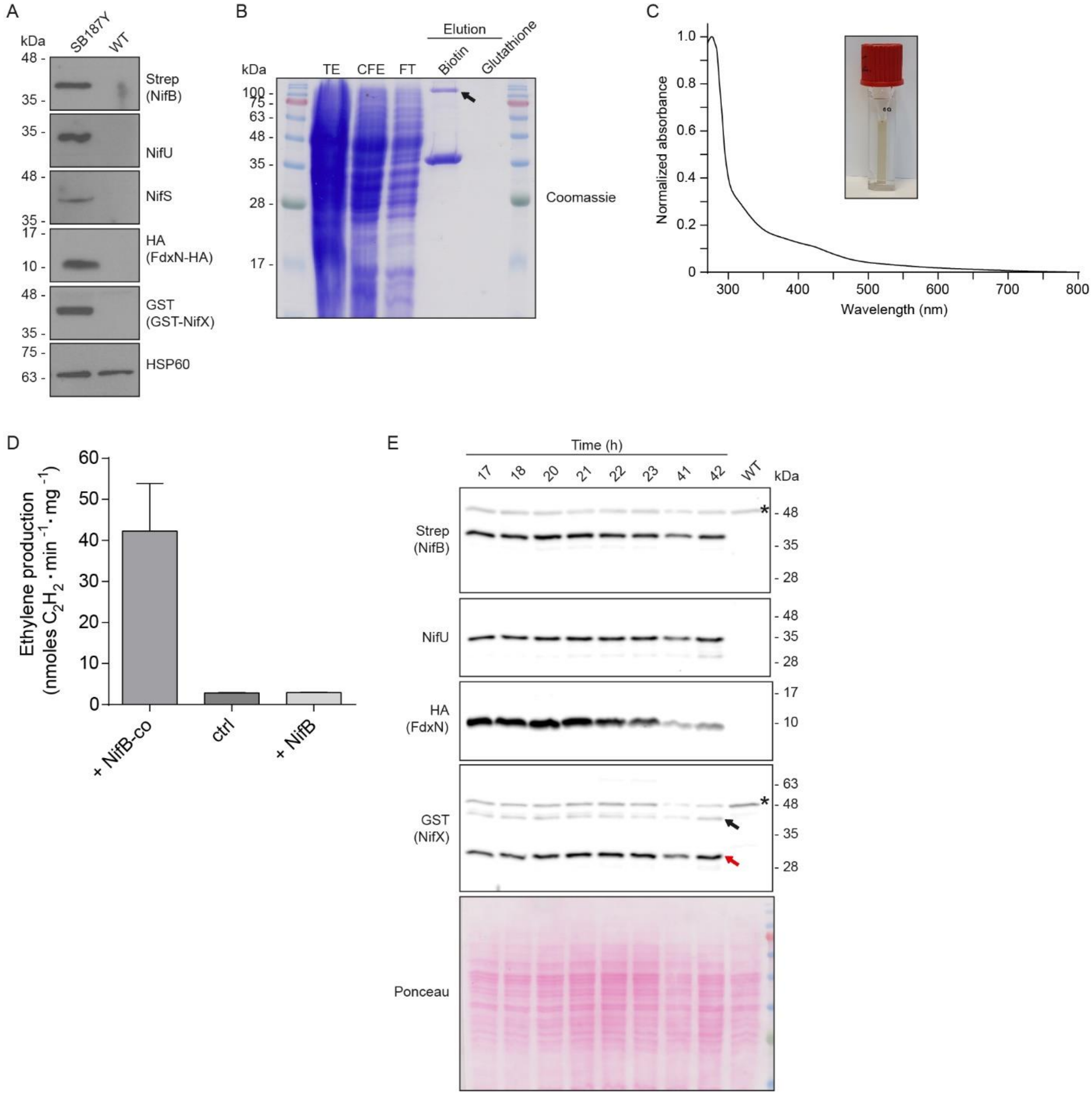

Fig. S7. (*A*) Western blot analysis of protein expression in total extracts of *S. cerevisiae* strain SB187Y (parental strain 3 expressing *M. infernus* NifB). Protein extract from wild-type *S. cerevisiae* (WT) was used as control for the specificity of the antibodies. (*B*) Example of purification of *M. infernus* NifB from SB187Y, following fermenter procedure 3 (*SI Appendix*, Supplementary Materials and Methods). TE, total protein extract; CFE, cell-free extract (supernatant after centrifugation of TE); FT, flow-through fraction, Biotin, biotin-eluted fraction; Glutathione, glutathione-eluted fraction. The band seen at the top of the gel (black arrow) is a contaminant often seen in our Strep-Tactin purifications of *S. cerevisiae* protein extracts, and corresponds to pyruvate carboxylase (PYC1) with theoretical mass 130.1 kDa. (*C*) UV-visible spectrum of *M. infernus* NifB purified from SB187Y. (*D*) NifB-dependent *in vitro* FeMo-co synthesis and apo-NifDK reconstitution using purified proteins. Five µM as-isolated *M. infernus* NifB purified from SB187Y (+ NifB) was used in the assay and compared to reactions where no NifB was added (ctrl). Positive control reactions for the assay contained NifB-co (+ NifB-co).

Error bars represent mean ± standard deviation (n=2). Specific activity of holo-NifDK determined under the same reaction conditions was 1,153 nmol ethylene formed per min and mg NifDK protein. (*E*) Western blot analysis of the expression of *M. infernus* NifB, NifU, FdxN, and GST-NifX in *S. cerevisiae* strain SB187Y during the fermenter process. Full-length GST-NifX (black arrow), a degradation variant of GST-NifX (red arrow), and an unspecific signal (star) are indicated.

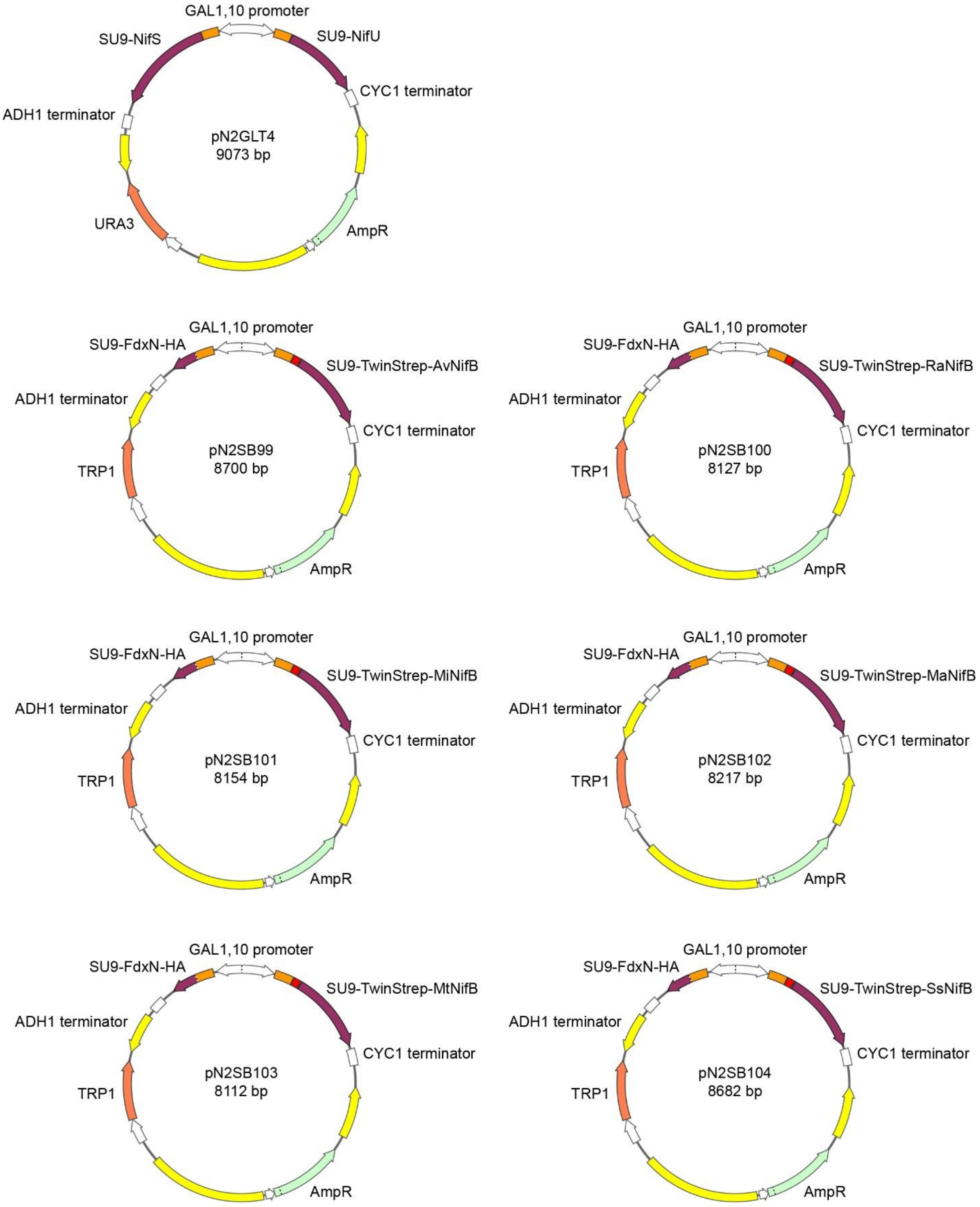

Fig. S8. Simplified DNA maps for yeast expression plasmids used in strains SB222Y, SB223Y, SB224Y, SB225Y, SB226Y and SB227Y.

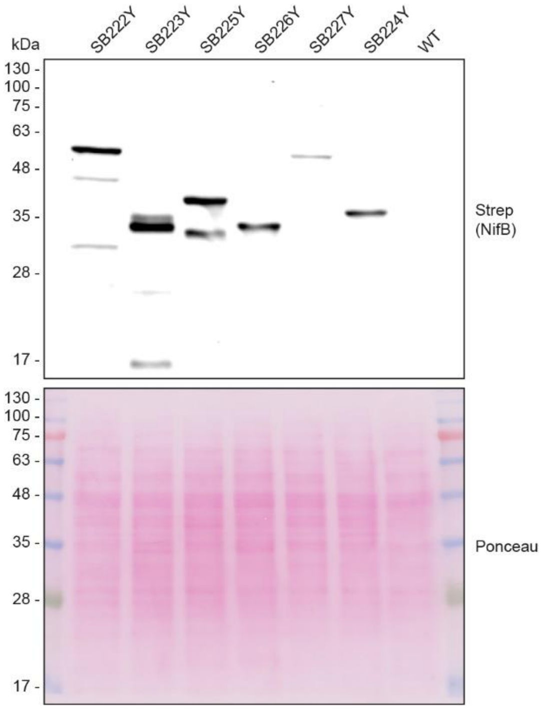

Fig. S9. Western blot analysis of GAL-induced NifB expression in total extracts of *S. cerevisiae* strains SB222Y, *A. variabilis* (57.2 kDa); SB223Y, *R. albus* SY3 (35.7 kDa); SB225Y, *M. acetivorans* (39.4 kDa); SB226Y, *M. thermautotrophicus* (35.7 kDa); SB227Y, *Synechococcus sp.* JA-3-3Ab (56.6 kDa); SB224Y, *M. infernus* (38.2 kDa). Sizes indicate expected molecular weight upon SU9-processing in the mitochondria. Protein extract from wild-type *S. cerevisiae* (WT) was used as control for the specificity of the antibodies.

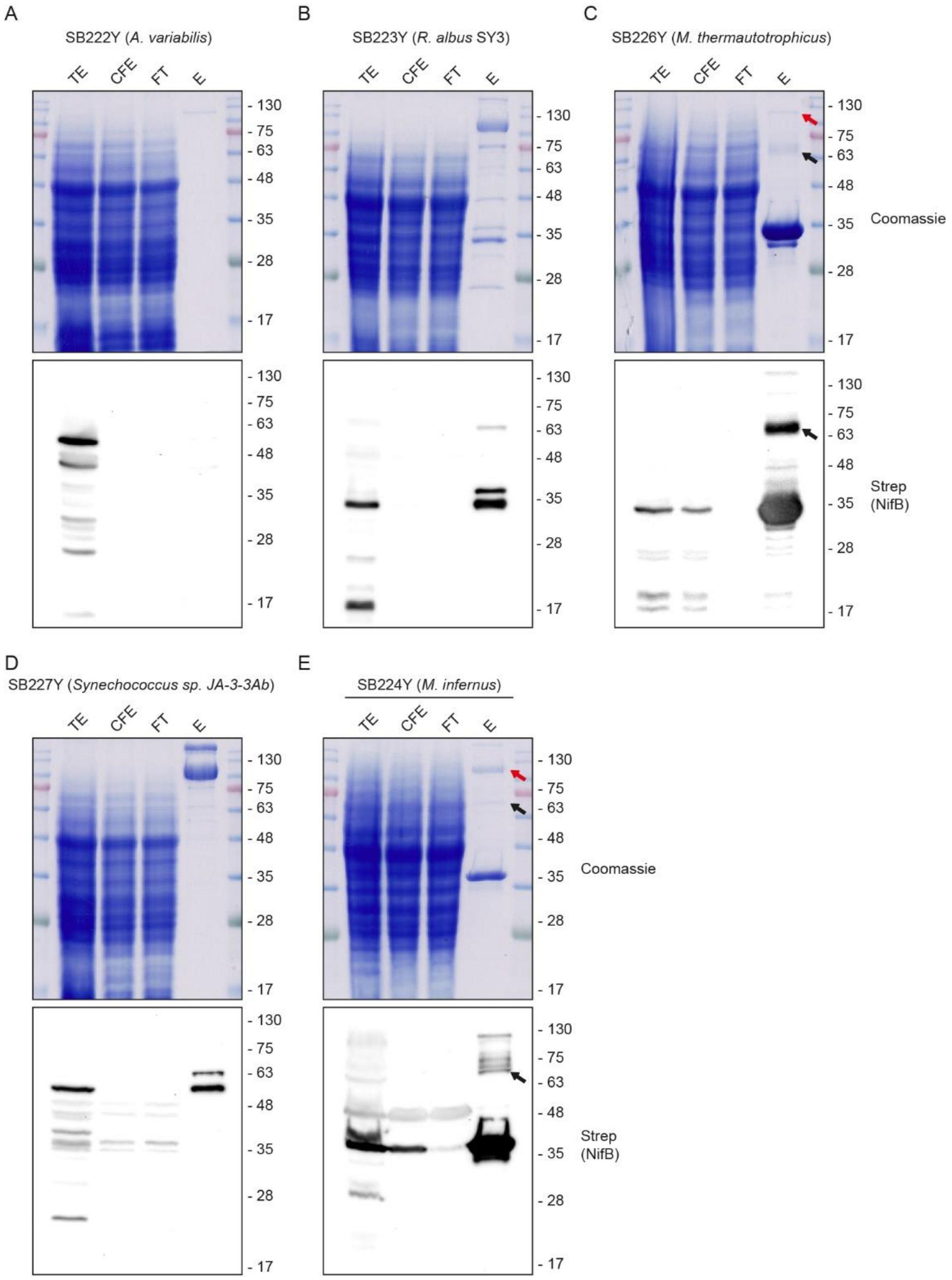

Fig. S10. GAL-induced NifB expression and Strep-Tactin purification, following fermenter procedure 2 (*SI Appendix*, Supplementary Materials and Methods). (*A*) SB222Y, *A. variabilis*, (*B*) SB223Y, *R. albus* SY3, (*C*) SB226Y, *M. thermautotrophicus*, (*D*) SB227Y, *Synechococcus sp.* JA-3-3Ab, (*E*) SB224Y, *M. infernus*. TE, total protein extract; CFE, cell-free extract (supernatant after centrifugation of TE); FT, Strep-Tactin flow-through fraction, E, biotin-eluted fraction. The bands are detected using both Coomassie stain and antibodies targeting the Strep-epitope (exemplified by black arrows in *C* and *E*) likely represent aggregates of NifB proteins. The band seen in the Coomassie gels migrating below the 130 kDa marker (exemplified by red arrows in *C* and *E*) is a contaminant often seen in our Strep-Tactin purifications of *S. cerevisiae* protein extracts, and corresponds to pyruvate carboxylase (PYC1) with theoretical mass 130.1

kDa. The NifB proteins from *M. thermautotrophicus* (*C*) and *M. infernus* (*E*) were mainly soluble and could be purified at high levels. Some NifB protein from Ruminococcus albus SY3 (*B*) and *Synechococcus sp.* JA-3-3Ab (*D*) could be found in the elution fractions although these NifB variants had low solubility.

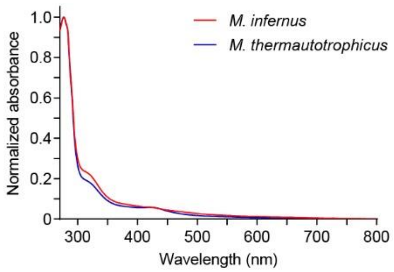

Fig. S11. UV-visible spectra of *M. infernus* and *M. thermautotrophicus* NifB proteins purified from *S. cerevisiae* strains SB224Y and SB226Y, respectively, following fermenter procedure 2 (*SI Appendix*, Supplementary Materials and Methods).

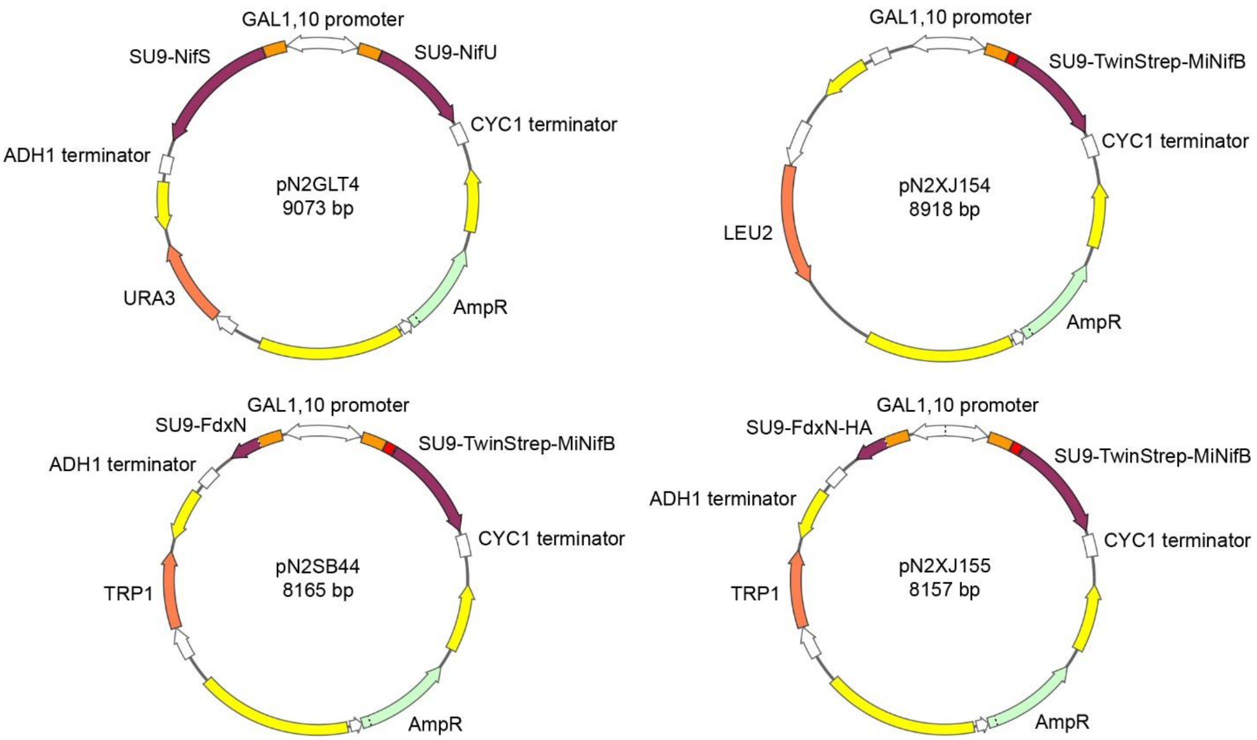

Fig. S12. Simplified DNA maps for yeast expression plasmids used in strains SB30Y, SB31Y, SB32Y and SB33Y.

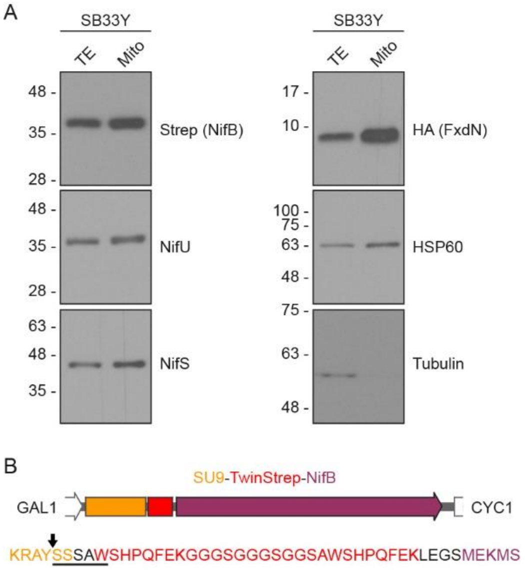

Fig. S13. (*A*) Immunoblot analysis of total extracts (TE) and mitochondria isolations (Mito) showing mitochondria targeting of NifB, NifU, NifS and FdxN-HA in SB33Y. Antibodies recognizing cytoplasmic (tubulin) and mitochondria (HSP60) control proteins are included. (*B*) SU9 processing site (black arrow) of NifB. Underlined sequence indicates the N-terminal amino acids identified by Edman degradation.

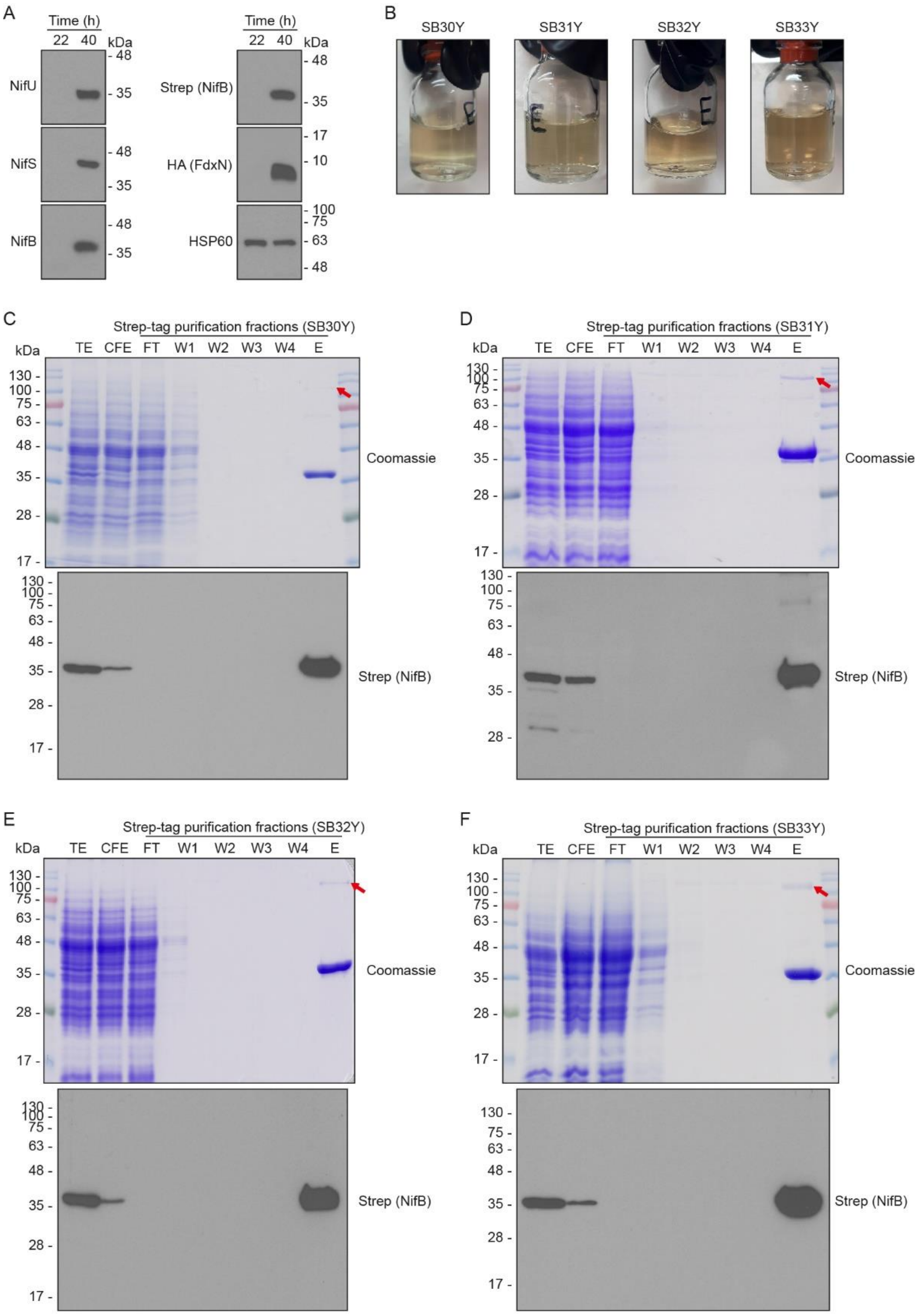

Fig. S14. Expression and purification of *M. infernus* NifB proteins. (*A*) Example of protein expression in fermenter-grown SB33Y (for expression of $NifB^{USF}$). 22 h and 40 h represents 22 h and 40 h after start of the fermenter. Galactose was added at t=22.5 h, and the cells were harvested at t=40 h. (*B*) Typical appearance of the corresponding NifB proteins purified from 100 g yeast cells following elution and desalting (total volume about 13 ml). (*C-F*) Coomassie

staining and immunoblot analysis of representative NifB purifications, following fermenter procedure 1 (*SI Appendix*, Supplementary Materials and Methods), from strain SB30Y (*C*) for expression of NifB*, strain SB31Y (*D*) for expression of $NifB^{US}$, strain SB32Y (*E*) for expression of $NifB^{F}$, and strain SB33Y (*F*) for expression of $NifB^{USF}$. TE, total extract; CFE, cell-free extract after centrifugation and filtering of TE; FT, flow-through fraction; W1-W4, chromatographic wash fractions; E, fraction following biotin elution. Molecular mass markers are indicated to the left and primary antibody to the right of each panel. The band seen in the Coomassie gels migrating below the 130 kDa marker (highlighted in *C*-*F* by red arrows) is a contaminant often seen in our Strep-Tactin purifications of *S. cerevisiae* protein extracts, and corresponds to pyruvate carboxylase (PYC1) with theoretical mass 130.1 kDa.

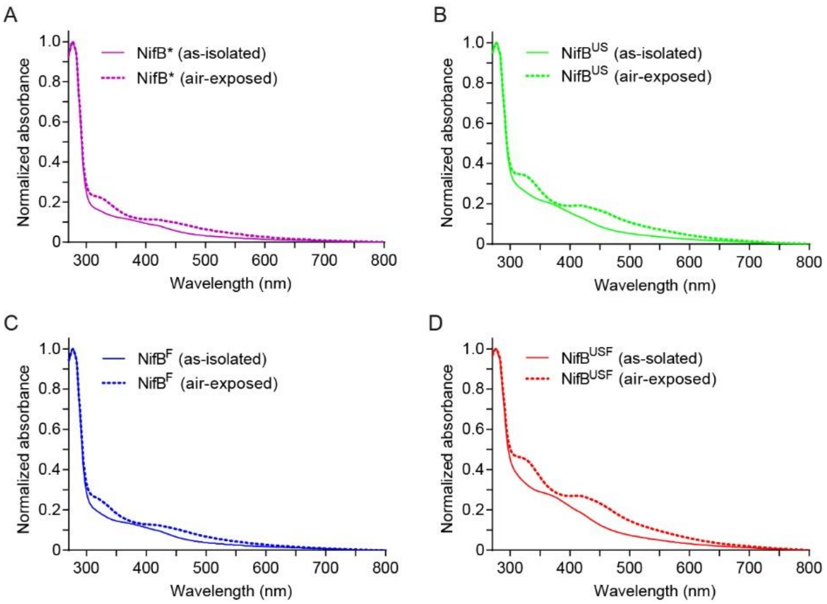

Fig. S15. As-isolated and air-exposed UV-visible spectra of *M. infernus* NifB proteins purified anaerobically from aerobically grown *S. cerevisiae*, following fermenter procedure 1 (*SI Appendix*, Supplementary Materials and Methods). (*A*) Strain SB30Y for expression of NifB*, (*B*) strain SB31Y for expression of $NifB^{US}$, (*C*) strain SB32Y for expression of $NifB^{F}$, and (*D*) strain SB33Y for expression of $NifB^{USF}$.

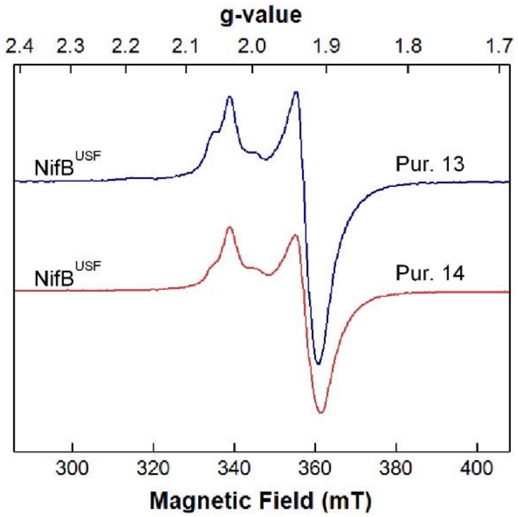

Fig. S16. 12 K X-band EPR spectra of two independent *M. infernus* NifB^{USF} preparations (see Table S5 for details).

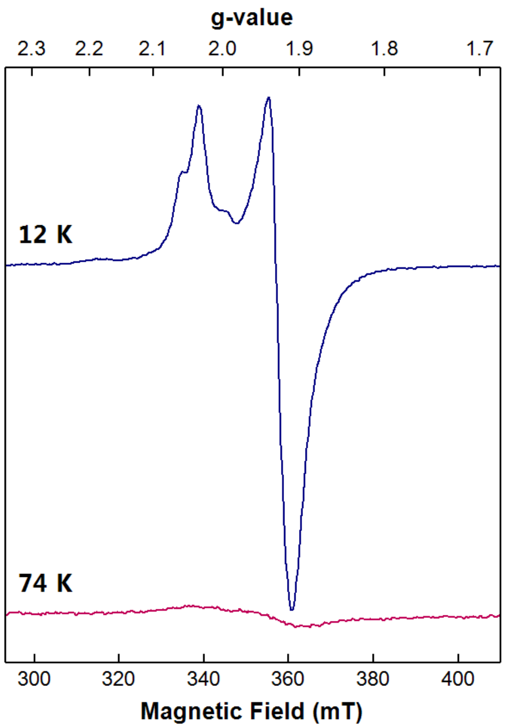

Fig. S17. X-band EPR spectra of *M. infernus* NifB^{USF} measured at two different temperatures.

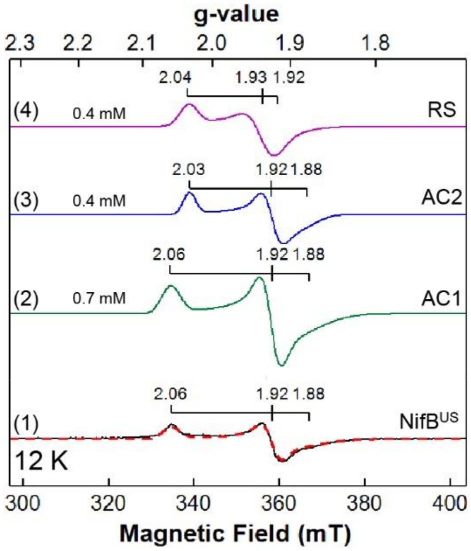

Fig. S18. X-band EPR spectra of *M. infernus* $NifB^{US}$ (purification 8+9, Table S5) (1), and subcomponents as in Fig. 3*E* (2 to 4). Experimental data is shown in black solid line, while overall spectral simulation (using AC1 cluster (10)) is shown in red dotted line. The *g* values of each species, spin concentration and cluster nomenclature (adapted from (10)) are indicated in the figure.

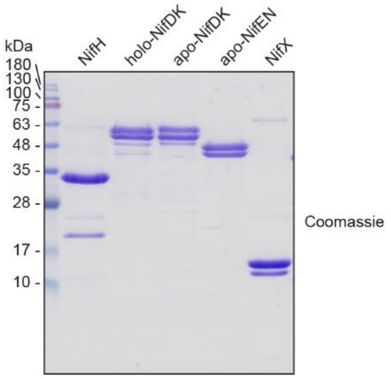

Fig. S19. Proteins used for NifB-dependent *in vitro* FeMo-co synthesis and apo-NifDK activation assays.

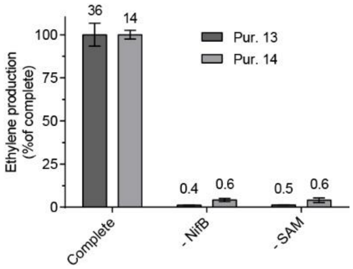

Fig. S20. Requirements for *M. infernus* $NifB^{USF}$-dependent *in vitro* FeMo-co synthesis and apo-NifDK reconstitution in a completely defined assay. Five µM *M. infernus* $NifB^{USF}$ were used per assay (purifications 13 and 14, Table S5). Activities are normalized to complete conditions (containing MoO_4^{2-}, *R*-homocitrate, Fe^{2+}, S^{2-}, SAM, DTH, apo-NifEN, apo-NifDK, NifX and NifH). Values above bars represent average nmol ethylene produced per min and mg apo-NifDK. Error bars represent mean ± standard deviation (n=2). Specific activity of holo-NifDK and NifB-co-dependent activated apo-NifDK determined under the same reaction conditions was 1,137 and 202 nmol ethylene formed per min and mg NifDK protein, respectively.

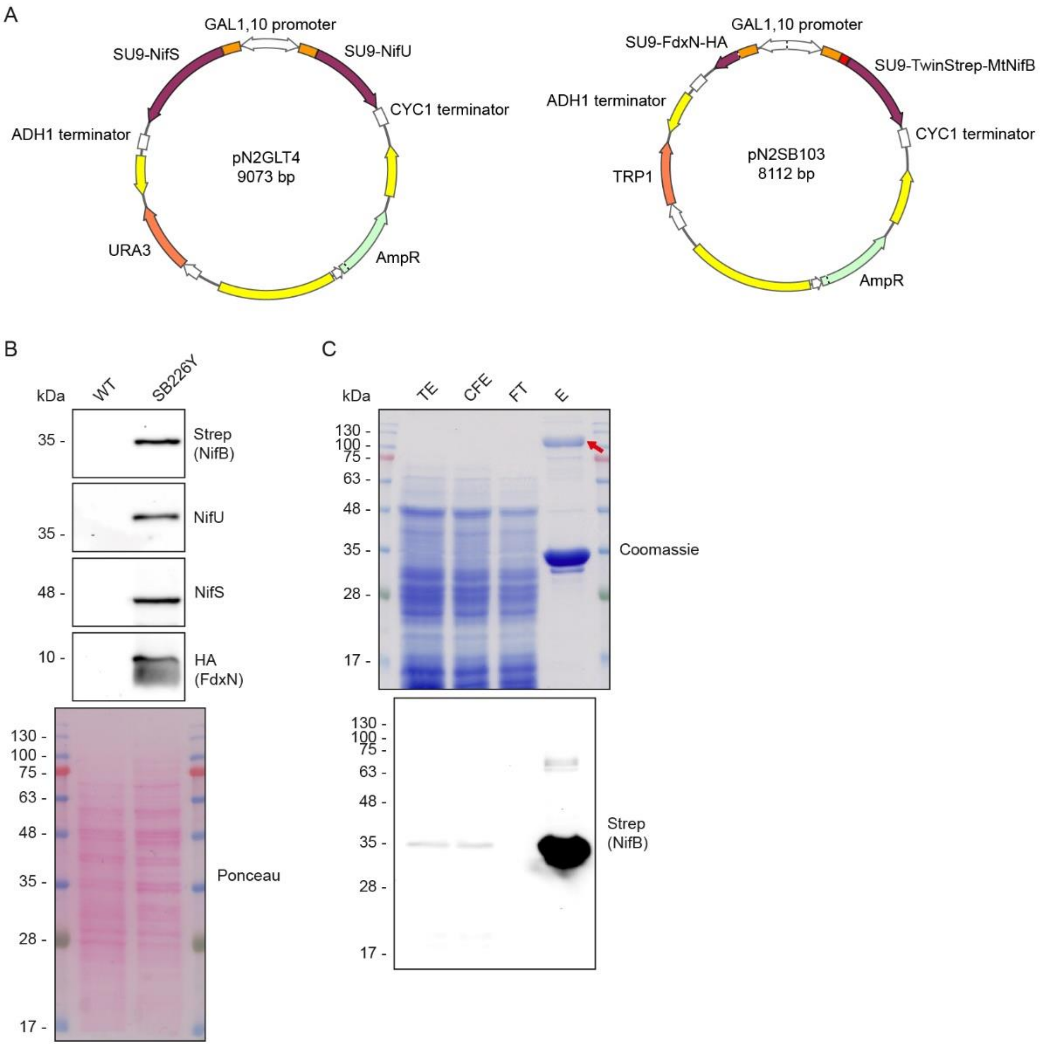

Fig. S21. Expression and purification of *M. thermautotrophicus* NifB^{USF} protein from *S. cerevisiae* strain SB226Y. (*A*) Simplified DNA maps for yeast expression plasmids used in strain SB226Y. (*B*) Western blot analysis of protein expression in total extracts of *S. cerevisiae* strain SB226Y. Protein extract from wild-type *S. cerevisiae* (WT) was used as control for the specificity of the antibodies. (*C*) Coomassie staining and Western blot analysis of a representative *M. thermautotrophicus* NifB^{USF} purification, TE, total extract; CFE, cell-free extract after centrifugation and filtering of TE; FT, flow-through fraction; E, fraction following biotin elution. The band seen in the Coomassie gels migrating below the 130 kDa marker (red arrow) is a contaminant often seen in our Strep-tactin purifications of *S. cerevisiae* protein extracts, and corresponds to pyruvate carboxylase (PYC1) with theoretical mass 130.1 kDa.

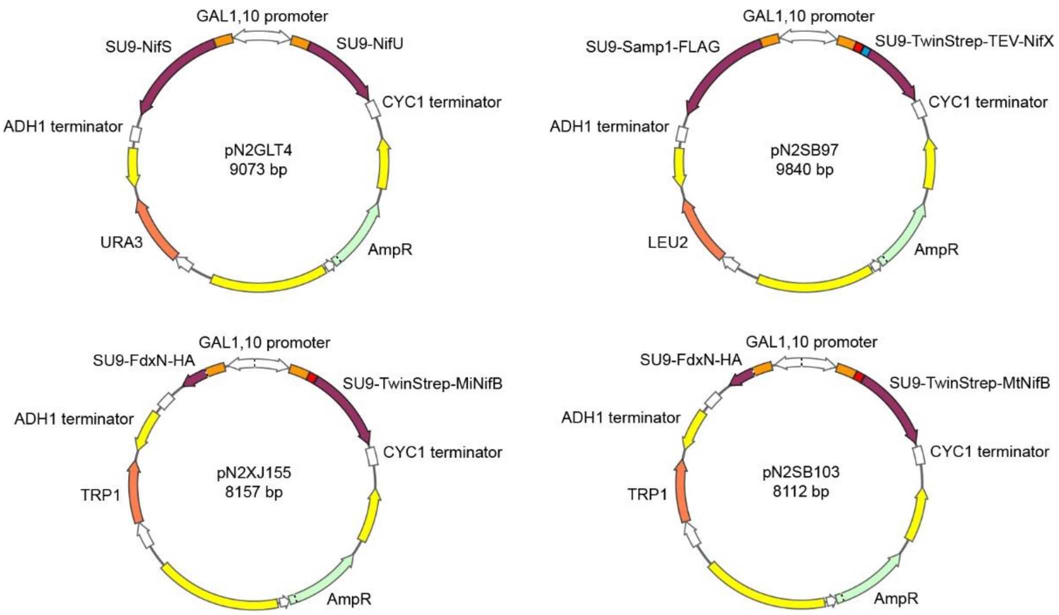

Fig. S22. Simplified DNA maps for yeast expression plasmids used in strain SB220Y and SB233Y.

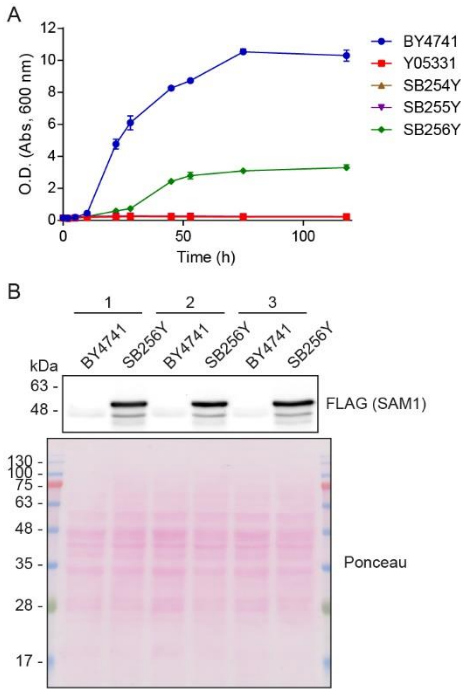

Fig. S23. Effect of SU9-Sam1p-FLAG on the growth of *sam5*Δ cells in YP media containing a non-fermentable carbon source (glycerol) and 0.1 μM β-estradiol (to mimic GAL induction using the pGEV-His plasmid). Wild-type BY4741 cells (BY4741), BY4741 cells with deleted *sam5* gene (Y05331), Y05331 cells transformed with pGEV-His plasmid (SB254Y), Y05331 cells transformed with pGEV-His and pN2SB106 plasmids (SB255Y, expressing SU9-TS-TEV-NifX and Sam1p-FLAG), and Y05331 cells transformed with pGEV-His and pN2SB97 plasmids (SB256Y, expressing SU9-TS-TEV-NifX and SU9-Sam1p-FLAG). (*B*) Immunoblot analysis of protein expression in total extracts of wild-type BY4741 cells (BY4741) and Y05331 cells transformed with pGEV-His and pN2SB97 plasmids (SB256Y, expressing SU9-TS-TEV-NifX and SU9-Sam1p-FLAG). Protein expression was analyzed at time-point 75 h.

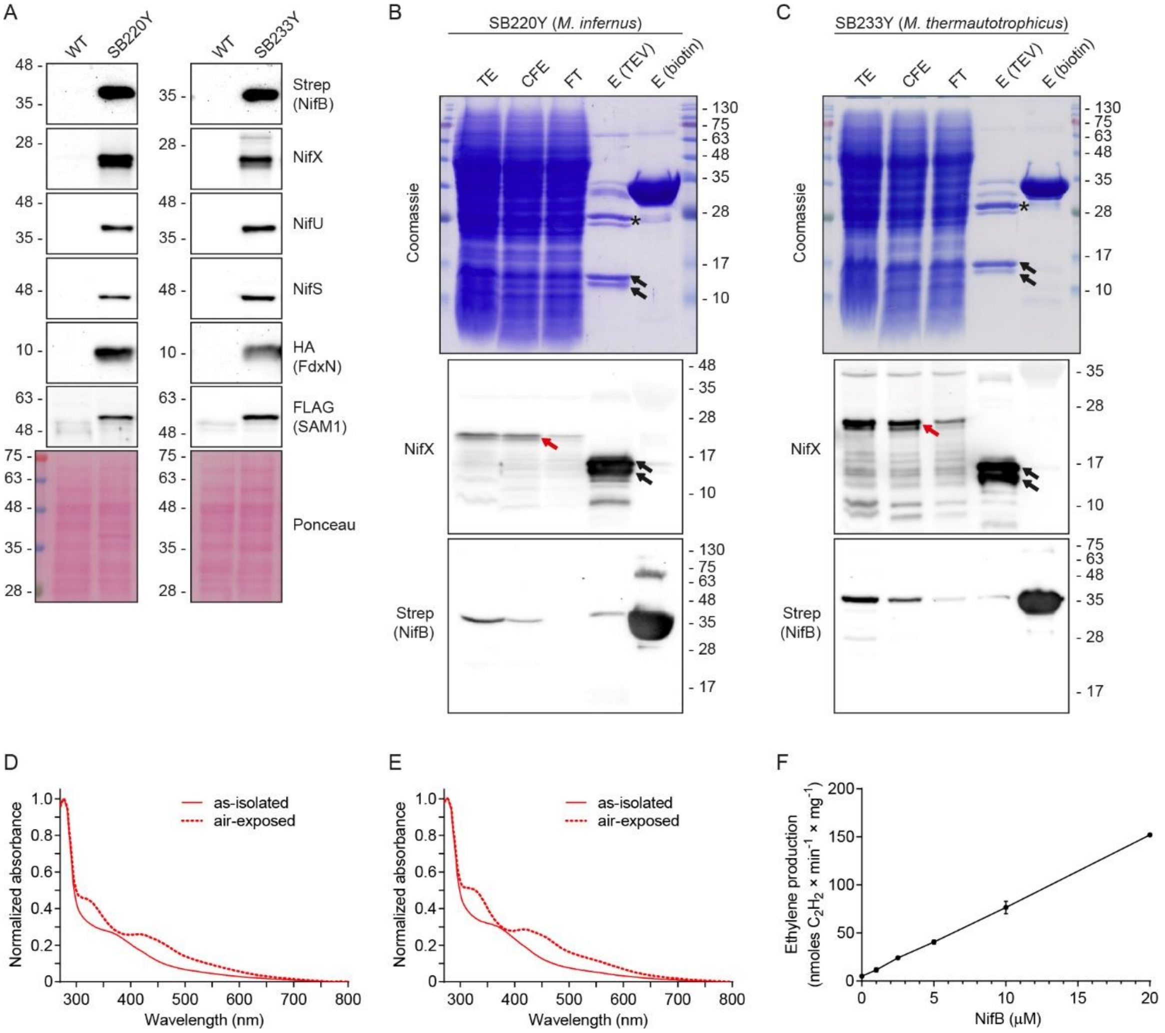

Fig. S24. (*A*) Immunoblot analysis of protein expression in total extracts of wild-type *S. cerevisiae* (WT) and *S. cerevisiae* strains used for NifX and NifB purifications (SB220Y, for isolation of NifX and *M. infernus* NifB$^{USF+SAM}$; SB233Y, for isolation of NifX and *M. thermautotrophicus* NifB$^{USF+SAM}$). (*B* and *C*) Coomassie staining (*B*) and Western blot analysis (*C*) of purification procedures to isolate NifX and *M. infernus* NifB$^{USF+SAM}$ (*B*), or NifX and *M. thermautotrophicus* NifB$^{USF+SAM}$ (*C*). TE, total extract; CFE, cell-free extract after centrifugation and filtering of TE; FT, flow-through fraction; E (TEV), fraction following TEV treatment; E (biotin), fraction following biotin elution. TEV is marked by black stars. Note the difference in size between TS-TEV-NifX (theoretical size 21.6 kDa, red arrow) and the TEV treated NifX (theoretical size 17.3 kDa, black arrows). (*D* and *E*) As-isolated and air-exposed UV-visible spectra of *M. infernus* NifB$^{USF+SAM}$ (*D*) and *M. thermautotrophicus* NifB$^{USF+SAM}$ (*E*). (*F*) Titration of *in vitro* FeMo-co synthesis and apo-NifDK reconstitution using purified proteins (see Materials and Methods for details) and as-isolated *M. thermautotrophicus* NifB$^{USF+SAM}$ protein. Activity is represented as nmol ethylene produced per min and mg NifDK. Error bars represent mean ± standard deviation (n=2, n=1 for 20 μM). Specific activities of holo-NifDK and NifB-co-dependent activated apo-

NifDK determined under the same reaction conditions were 1,148 and 417 nmol ethylene formed per min and mg NifDK protein, respectively.

Supplementary Tables

Table S1. Composition/design of parental library strains (PS).

PS	Gene 1	Gene 2	Gene 3	Gene 4
1	P1-SU9-NifS-T14	P24-SU9-NifU-T4	P7-SU9-GST-NifX-T12	P21-SU9-FdxN-T20
2	P1-SU9-NifS-T14	P24-SU9-NifU-T4	P9-SU9-GST-NifX-T19	P18-SU9-FdxN-T6
3	P1-SU9-NifS-T14	P24-SU9-NifU-T4	P22-SU9-GST-NifX-T7	P6-SU9-FdxN-T1
4	P1-SU9-NifS-T14	P24-SU9-NifU-T4	P21-SU9-GST-NifX-T20	P31-SU9-FdxN-T1
5	P1-SU9-NifS-T14	P24-SU9-NifU-T4	P18-SU9-GST-NifX-T6	P15-SU9-FdxN-T24
6	P1-SU9-NifS-T14	P24-SU9-NifU-T4	P6-SU9-GST-NifX-T1	P5-SU9-FdxN-T24

Table S2. Promoters and terminators used for parental library strains and NifB library. Promoters and terminators refer to previously published sequences (6).

Part Abbreviation	Function	Part name	Source organism
P1	Promoter	ACT1	*Saccharomyces cerevisiae*
P5	Promoter	CHO1	*Saccharomyces cerevisiae*
P6	Promoter	EFT2	*Saccharomyces cerevisiae*
P7	Promoter	FBA1	*Saccharomyces cerevisiae*
P9	Promoter	HHF2	*Saccharomyces cerevisiae*
P15	Promoter	PFY1	*Saccharomyces cerevisiae*
P17	Promoter	PXR1	*Saccharomyces cerevisiae*
P18	Promoter	RPL28	*Saccharomyces cerevisiae*
P21	Promoter	RPS9A	*Saccharomyces cerevisiae*
P22	Promoter	SbTDH3	*Saccharomyces bayanus*
P24	Promoter	SpTDH3	*Saccharomyces paradoxus*
P26	Promoter	TEF1	*Saccharomyces cerevisiae*
P31	Promoter	KlURA3	*Kluyveromyces lactis*
T1	Terminator	ADH1	*Saccharomyces cerevisiae*
T4	Terminator	CYC1	*Saccharomyces cerevisiae*
T6	Terminator	ECM10	*Saccharomyces cerevisiae*
T7	Terminator	EFM1	*Saccharomyces cerevisiae*
T12	Terminator	RPL15A	*Saccharomyces cerevisiae*
T14	Terminator	RPL41B	*Saccharomyces cerevisiae*
T19	Terminator	VMA2	*Saccharomyces cerevisiae*
T20	Terminator	YHI9	*Saccharomyces cerevisiae*
T22	Terminator	KlURA3	*Kluyveromyces lactis*
T24	Terminator	ADH2	*Saccharomyces cerevisiae*

Table S3. Summary of NifB library screening.

Domain	Phylum	Organism	NifX	Expr.	Sol.	Pur.
archaea	euryarch.	*M. infernus*	-	YES	YES	HIGH
archaea	euryarch.	*M. acetivorans*	-	YES	YES	-
archaea	euryarch.	*M. thermautotrophicus*	-	YES	YES	HIGH
bacteria	α-bact.	*R. leguminosarum bv. trifolii (WSM2304)*	-	YES	-	-
bacteria	α-bact.	*D. vulgaris*	-	YES	-	-
bacteria	α-bact.	*B. diazoefficiens*	YES	YES	-	-
bacteria	α-bact.	*R. leguminosarum bv. trifolii (WSM1325)*	YES	-	-	-
bacteria	α-bact.	*S. meliloti*	YES	-	-	-
bacteria	α-bact.	*G. diazotrophicus*	YES	-	-	-
bacteria	α-bact.	*R. palustris*	YES	-	-	-
bacteria	α-bact.	*R. capsulatus*	YES	YES	-	-
bacteria	chlor.	*C. tepidum*	YES	-	-	-
bacteria	cyano.	*Nostoc sp. (PCC 7120)*	YES	-	-	-
bacteria	cyano.	*Gloeothece sp. (KO68DGA)*	YES	YES	-	-
bacteria	cyano.	*Cyanothece sp. (PCC 8801)*	YES	-	-	-
bacteria	cyano.	*Cyanothece sp. (ATCC 51142)*	YES	-	-	-
bacteria	cyano.	*A. variabilis*	YES	YES	YES	NO
bacteria	cyano.	*Synechococcus sp. (JA-3-3Ab)*	YES	YES	YES	NO
bacteria	cyano.	*N. azollae*	YES	YES	-	-
bacteria	cyano.	*Cyanothece sp. (PCC 7425)*	YES	YES	-	-
bacteria	δ-bact.	*G. sulfurreducens*	-	-	-	-
bacteria	δ-bact.	*S. fumaroxidans*	-	YES	-	-
bacteria	δ-bact.	*G. metallireducens*	-	YES	-	-
bacteria	firmi.	*R. albus SY3*	-	YES	YES	LOW
bacteria	firmi.	*P. sabinae*	YES	-	-	-
bacteria	γ-bact.	*A. vinelandii*	YES	-	-	-
bacteria	γ-bact.	*P. stutzeri*	YES	-	-	-
bacteria	chlorofl.	*Roseiflexus sp. (RS-1)*	-	YES	-	-

Origin of the 28 *nifB* genes selected for the library screening (euryarch., euryarchaeota; α-bact., α-proteobacteria; chlor., chlorobi; cyano., cyanobacteria; δ-bact., δ-proteobacteria; firmi., firmicutes; γ-bact., γ-proteobacteria; chlorofl., chloroflexi). Multidomain NifB protein architecture (presence of the C-terminal NifX-like domain) is indicated as NifX. Expression and accumulation of soluble NifB, and yield when purified using GAL-regulated expression plasmids, are indicated.

Table S4. Yeast strains with expression vectors used in this work.

Strain	Plasmids	Expressed proteins	Promoter	Full-length (kDa)	Processed (kDa)
SB17Y	pN2GLT4	SU9-NifU	GAL1	40.8	33.6
		SU9-NifS	GAL10	51.3	44.0
	pN2SB44	SU9-TS-MiNifB	GAL1	45.9	38.6
		SU9-FdxN	GAL10	17.1	9.8
SB18Y	pN2GLT4	SU9-NifU	GAL1	40.8	33.6
		SU9-NifS	GAL10	51.3	44.0
	pN2SB45	SU9-S-MiNifB	GAL1	44.1	36.8
		SU9-FdxN	GAL10	17.1	9.8
SB19Y	pN2GLT4	SU9-NifU	GAL1	40.8	33.6
		SU9-NifS	GAL10	51.3	44.0
	pN2SB46	SU9-10xHis-MiNifB	GAL1	44.2	36.8
		SU9-FdxN	GAL10	17.1	9.8
SB30Y	pN2XJ154	SU9-TS-MiNifB (=NifB*)	GAL1	45.9	38.6
SB31Y	pN2GLT4	SU9-NifU	GAL1	40.8	33.6
		SU9-NifS	GAL10	51.3	44.0
	pN2XJ154	SU9-TS-MiNifB (=NifB^{US})	GAL1	45.9	38.6
SB32Y	pN2XJ155	SU9-TS-MiNifB (=NifB^{F})	GAL1	45.9	38.6
		SU9-FdxN-HA	GAL10	18.2	10.9
SB33Y	pN2GLT4	SU9-NifU	GAL1	40.8	33.6
		SU9-NifS	GAL10	51.3	44.0
	pN2XJ155	SU9-TS-MiNifB (=NifB^{USF})	GAL1	45.9	38.6
		SU9-FdxN-HA	GAL10	18.2	10.9
SB220Y	pN2GLT4	SU9-NifU	GAL1	40.8	33.6
		SU9-NifS	GAL10	51.3	44.0
	pN2XJ155	SU9-TS-MiNifB (=NifB$^{USF+SAM}$)	GAL1	45.9	38.6
		SU9-FdxN-HA	GAL10	18.2	10.9
	pN2SB97	SU9-TS-TEV-NifX	GAL1	29.0	21.6 (17.3)
		SU9-Sam1p-FLAG	GAL10	50.9	43.4
SB222Y	pN2GLT4	SU9-NifU	GAL1	40.8	33.6
		SU9-NifS	GAL10	51.3	44.0
	pN2SB99	SU9-TS-AvNifB	GAL1	64.4	57.2
		SU9-FdxN-HA	GAL10	18.2	10.9
SB223Y	pN2GLT4	SU9-NifU	GAL1	40.8	33.6
		SU9-NifS	GAL10	51.3	44.0
	pN2SB100	SU9-TS-RaNifB	GAL1	43.0	35.7
		SU9-FdxN-HA	GAL10	18.2	10.9
SB224Y	pN2GLT4	SU9-NifU	GAL1	40.8	33.6
		SU9-NifS	GAL10	51.3	44.0
	pN2SB101	SU9-TS-MiNifB	GAL1	45.5	38.2
		SU9-FdxN-HA	GAL10	18.2	10.9
SB225Y	pN2GLT4	SU9-NifU	GAL1	40.8	33.6
		SU9-NifS	GAL10	51.3	44.0
	pN2SB102	SU9-TS-MaNifB	GAL1	46.7	39.4
		SU9-FdxN-HA	GAL10	18.2	10.9

SB226Y	pN2GLT4	SU9-NifU	GAL1	40.8	33.6
		SU9-NifS	GAL10	51.3	44.0
	pN2SB103	SU9-TS-MtNifB	GAL1	42.9	35.7
		SU9-FdxN-HA	GAL10	18.2	10.9
SB227Y	pN2GLT4	SU9-NifU	GAL1	40.8	33.6
		SU9-NifS	GAL10	51.3	44.0
	pN2SB104	SU9-TS-SsNifB	GAL1	63.9	56.6
		SU9-FdxN-HA	GAL10	18.2	10.9
SB233Y	pN2GLT4	SU9-NifU	GAL1	40.8	33.6
		SU9-NifS	GAL10	51.3	44.0
	pN2SB103	SU9-TS-MtNifB	GAL1	42.9	35.7
		SU9-FdxN-HA	GAL10	18.2	10.9
	pN2SB97	SU9-TS-TEV-NifX	GAL1	29.0	21.6 (17.3)
		SU9-Sam1p-FLAG	GAL10	50.9	43.4
SB254Y	pGEV-His	vector encoding the β-estradiol inducible activator (2)			
SB255Y	pGEV-His	vector encoding the β-estradiol inducible activator (2)			
	pN2SB106	SU9-TS-TEV-NifX	GAL1	29.0	21.6 (17.3)
		Sam1p-FLAG	GAL10	43.4	-
SB256Y	pGEV-His	vector encoding the β-estradiol inducible activator (2)			
	pN2SB97	SU9-TS-TEV-NifX	GAL1	29.0	21.6 (17.3)
		SU9-Sam1p-FLAG	GAL10	50.9	43.4

Processed forms refer to molecular mass following SU9 cleavage (RAY-SSAW, *SI Appendix*, Fig. S13*B*). For SU9-TS-TEV-NifX two values are reported in the "Processed" column where the smaller represent size after TEV protease treatment.

Table S5. Summary of yeast *M. infernus* NifB purifications.

Strain	Expr. NifB	Pur.	Yield (mg NifB per 100 g cells)	Fe (per NifB monomer)	$\mathbf{Yield_{av}}$ (mg NifB per 100 g cells)	$\mathbf{Fe_{av}}$ (per NifB monomer)
SB30Y	NifB*	1	4.67	2.10	6.38 ± 1.49	2.01 ± 0.13
		6	7.42	1.86		
		7	7.05	2.07		
SB31Y	$NifB^{US}$	2	5.68	4.95	6.16 ± 0.59	5.02 ± 0.12
		3	5.69	4.93		
		8	7.02	5.02		
		9	5.95	5.19		
SB32Y	$NifB^{F}$	4	7.17	2.34	6.52 ± 0.84	2.22 ± 0.30
		5	6.82	2.43		
		11	5.57	1.87		
SB33Y	$NifB^{USF}$	10	7.43	6.43	6.26 ± 1.46	6.11 ± 0.89
		12	4.37	4.87		
		13	7.38	6.98		
		14	5.87	6.16		

M. infernus NifB expressing strains and eventual coexpressed NifB accessory proteins, together with the yield of NifB per 100 g cells and Fe per NifB monomer, are indicated. Data for average values represent mean ± standard deviation.

Supplementary Dataset

Dataset S1. Origin, sequence and protein characteristics for the 28 NifB proteins tested in this study. Isoelectric point and instability index were calculated using the ProtParam online tool (11).

Supplementary References

1. Buren S, Jiang X, Lopez-Torrejon G, Echavarri-Erasun C, Rubio LM (2017) Purification and In Vitro Activity of Mitochondria Targeted Nitrogenase Cofactor Maturase NifB. *Front Plant Sci* 8(September):1567.
2. Gao CY, Pinkham JL (2000) Tightly regulated, beta-estradiol dose-dependent expression system for yeast. *Biotechniques* 29(6):1226–1231.
3. von der Haar T (2007) Optimized protein extraction for quantitative proteomics of yeasts. *PLoS One* 2(10):e1078.
4. Diekert K, de Kroon AI, Kispal G, Lill R (2001) Isolation and subfractionation of mitochondria from the yeast Saccharomyces cerevisiae. *Methods Cell Biol* 65:37–51.
5. Lopez-Torrejon G, et al. (2016) Expression of a functional oxygen-labile nitrogenase component in the mitochondrial matrix of aerobically grown yeast. *Nat Commun* 7:11426.
6. Burén S, et al. (2017) Formation of Nitrogenase NifDK Tetramers in the Mitochondria of Saccharomyces cerevisiae. *ACS Synth Biol* 6(6):1043–1055.
7. Marobbio CMT, Agrimi G, Lasorsa FM, Palmieri F (2003) Identification and functional reconstitution of yeast mitochondrial carrier for S-adenosylmethionine. *EMBO J* 22(22):5975–5982.
8. Fish WW (1988) Rapid colorimetric micromethod for the quantitation of complexed iron in biological samples. *Methods Enzymol* 158:357–364.
9. Hill HD, Straka JG (1988) Protein determination using bicinchoninic acid in the presence of sulfhydryl reagents. *Anal Biochem* 170(1):203–208.
10. Wilcoxen J, et al. (2016) Electron Paramagnetic Resonance Characterization of Three Iron–Sulfur Clusters Present in the Nitrogenase Cofactor Maturase NifB from Methanocaldococcus infernus. *J Am Chem Soc* 138(24):7468–7471.
11. Gasteiger E, et al. (2005) Protein Identification and Analysis Tools on the ExPASy Server. *The Proteomics Protocols Handbook*, ed Walker JM (Humana Press, Totowa, NJ), pp 571–607.
